# Supplementary material for: Testing the Domino Theory of Gene Loss in Buchnera aphidicola: The Relevance of Epistatic Interactions
Source: Life (Basel). 2018 May 29;8(2):17. doi: 10.3390/life8020017 (PMC6027505; doi:10.3390/life8020017)
Supplement: Supplementary file 1 [file life-08-00017-s001.zip › Martinez-Cano-etal-SuppFiles/Supplementary_material_1.pdf]

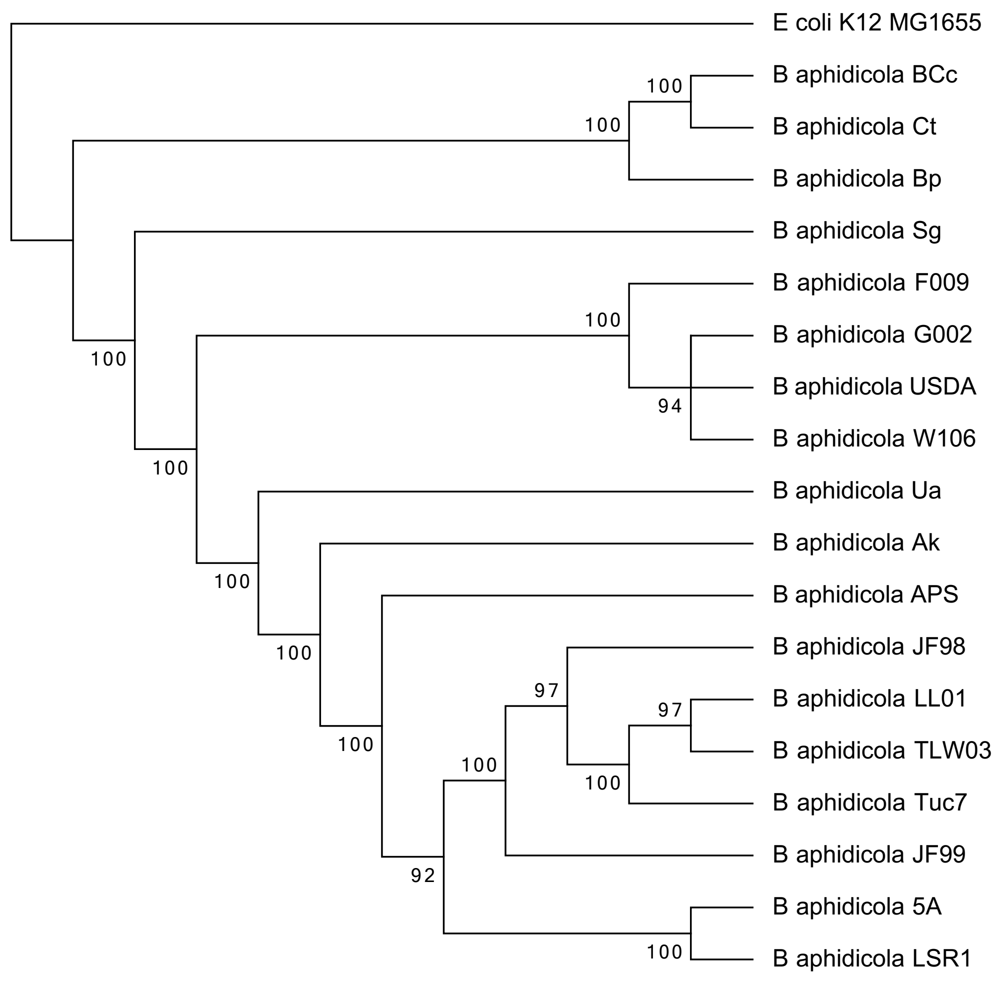

Phylogenetic tree of the *Buchnera*. The tree was reconstructed with MrBayes (see methods). Node numbers indicate posterior probabilities.

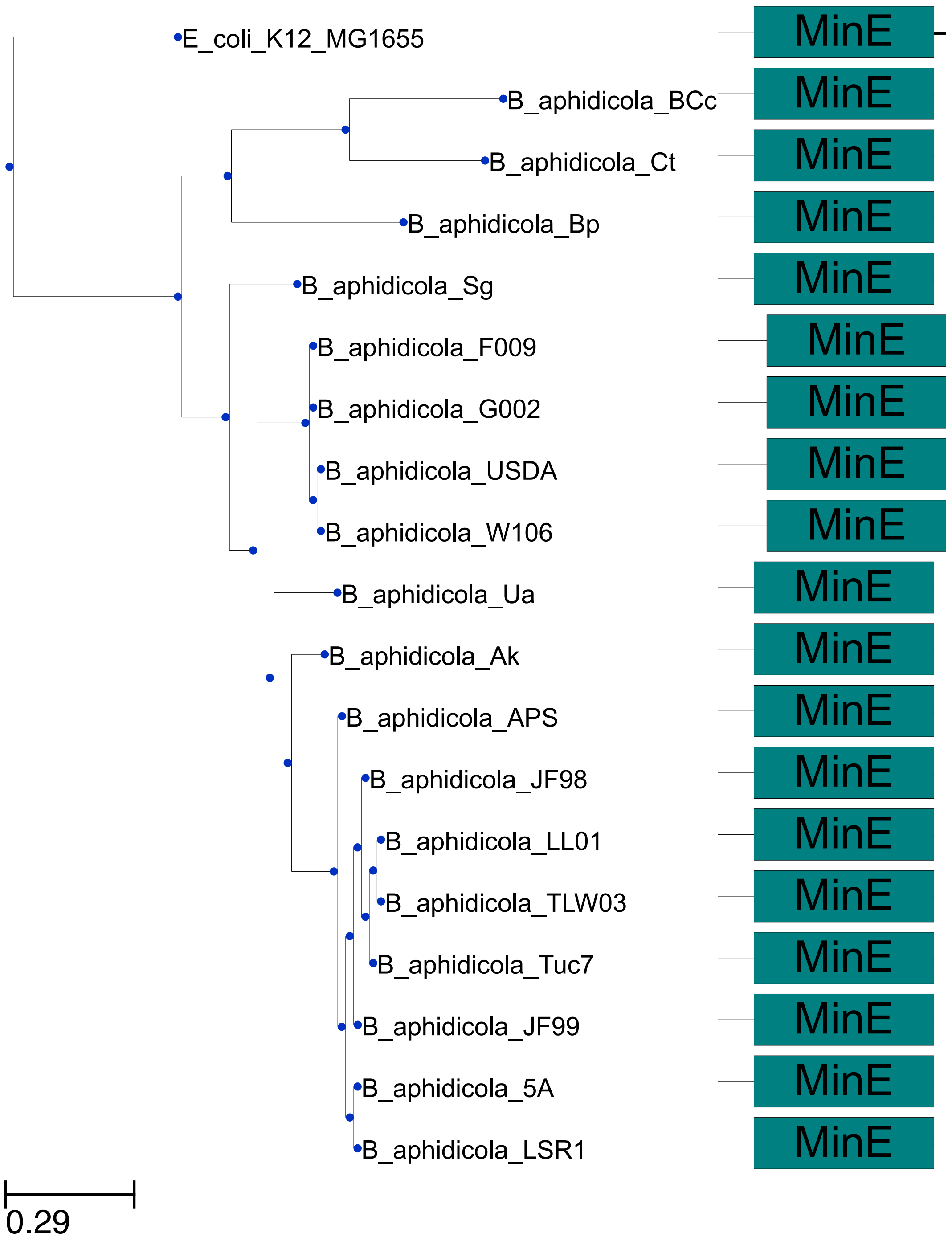

minE
NP_415692.1
cell division topological specificity factor.

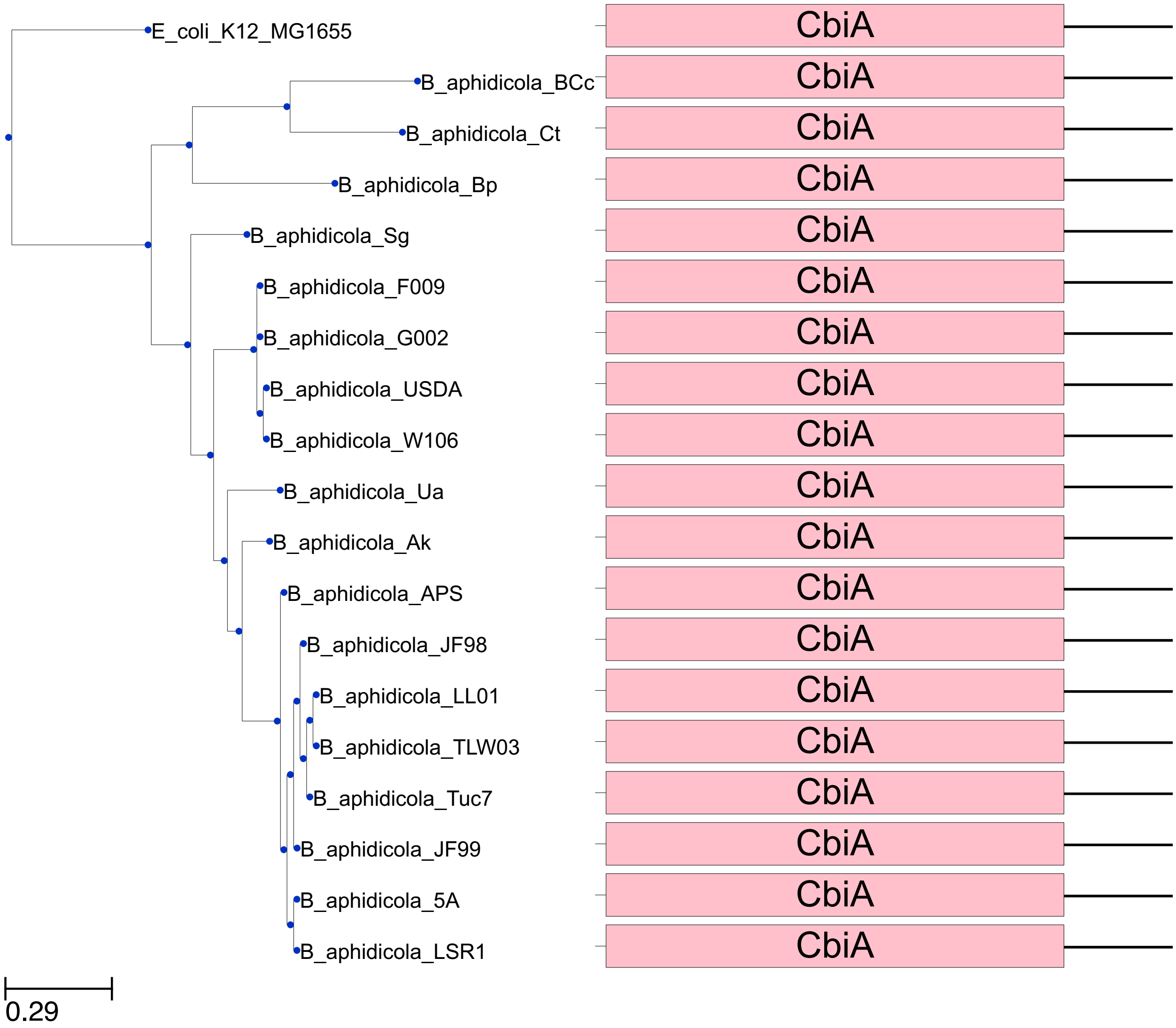

minD

NP_415693.1

inhibitor of FtsZ ring polymerization; chromosome-membrane tethering protein; membrane ATPase of the MinCDEE system

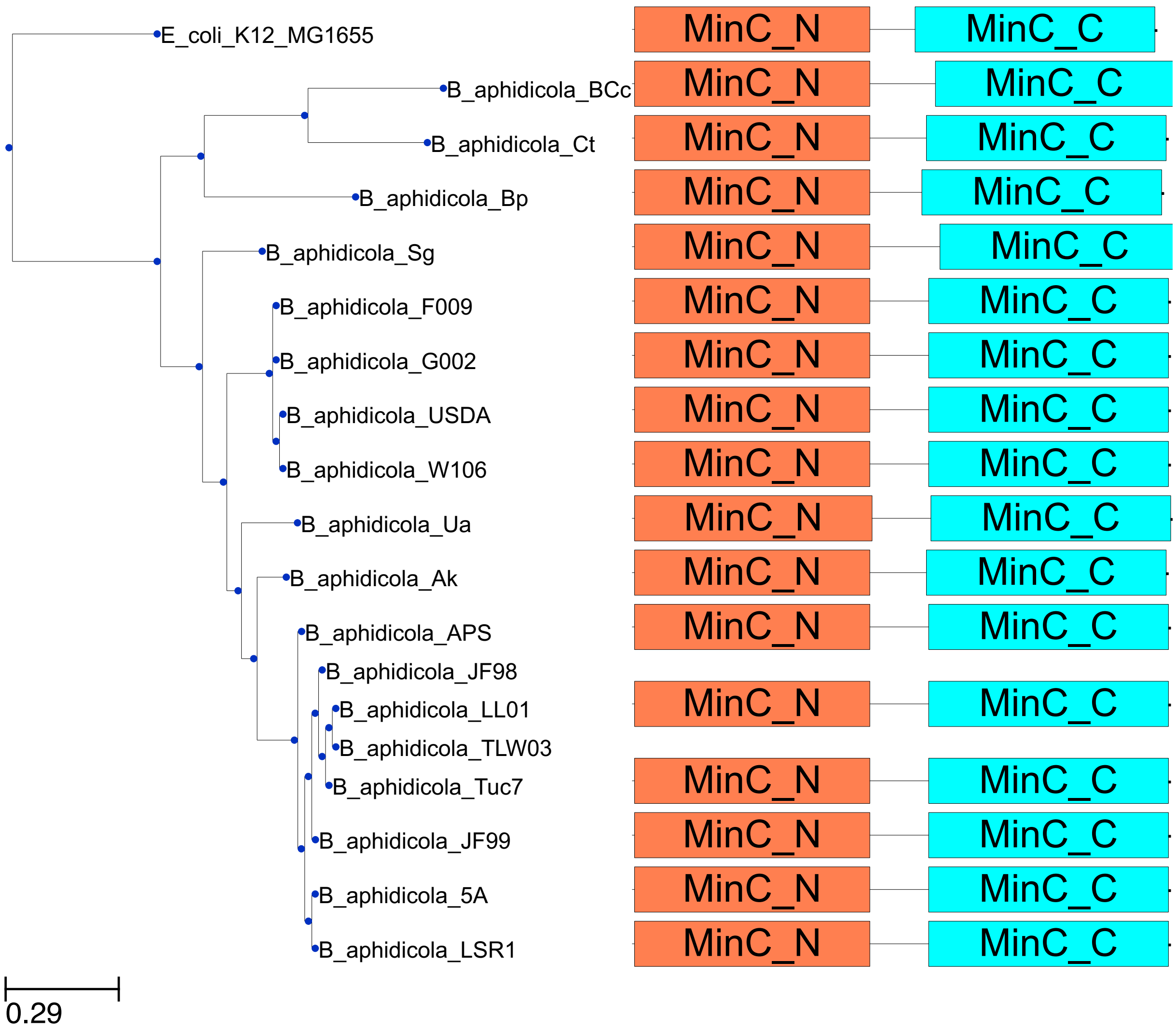

minC
NP_415694.1
inhibitor of FtsZ ring polymerization

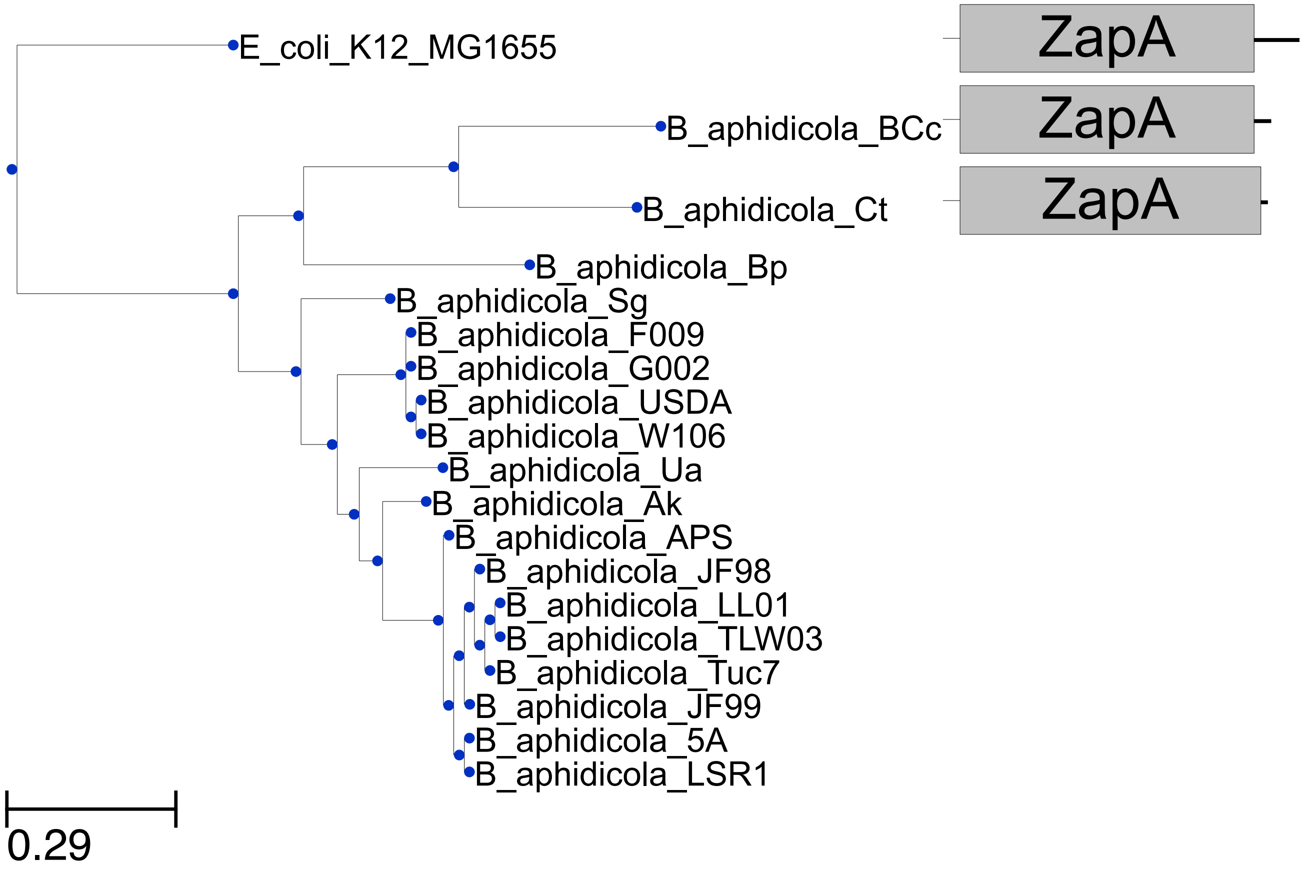

zapA
NP_417386.1
FtsZ stabilizer

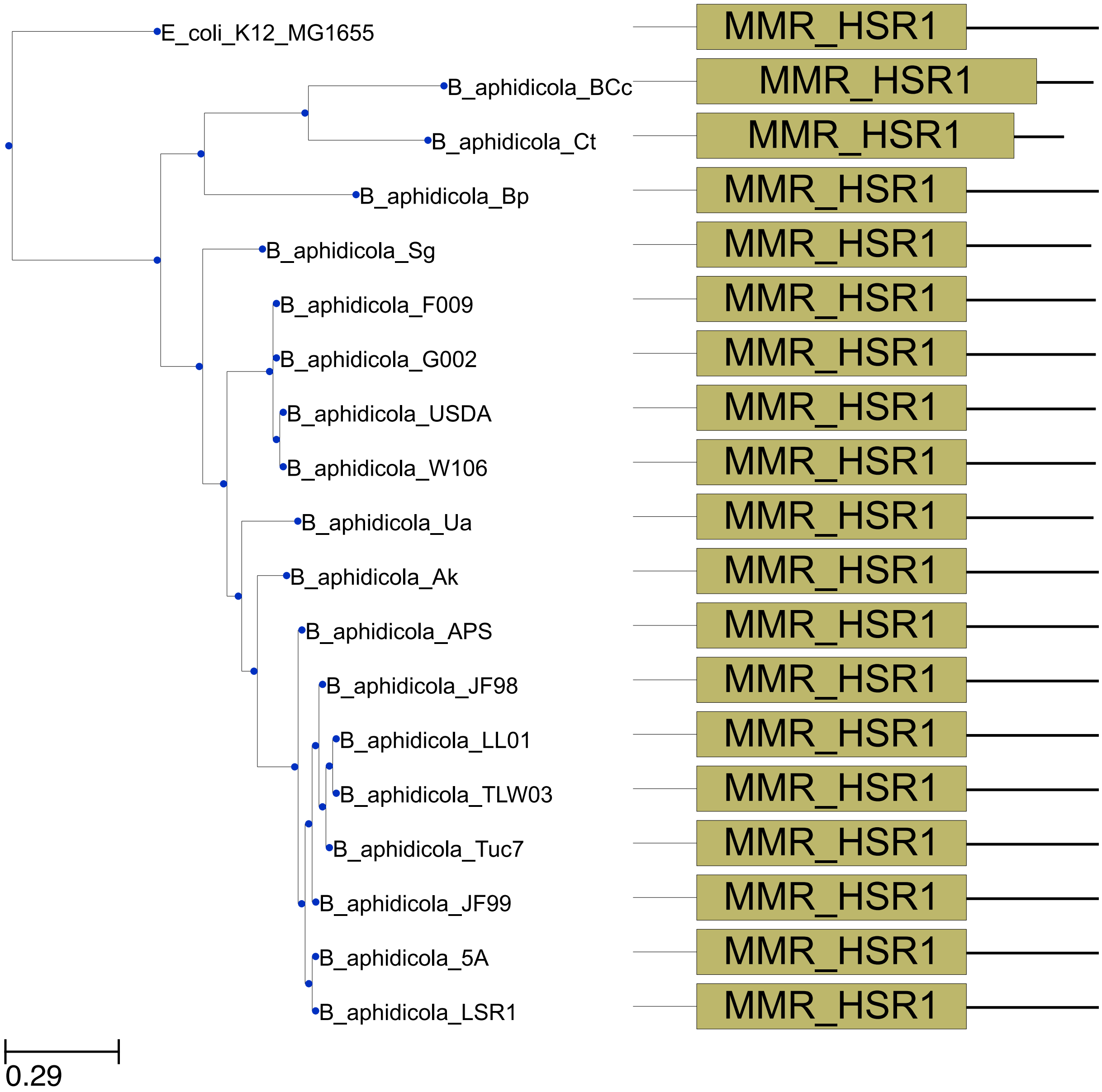

yihA
NP_418301.3
cell division GTP-binding protein

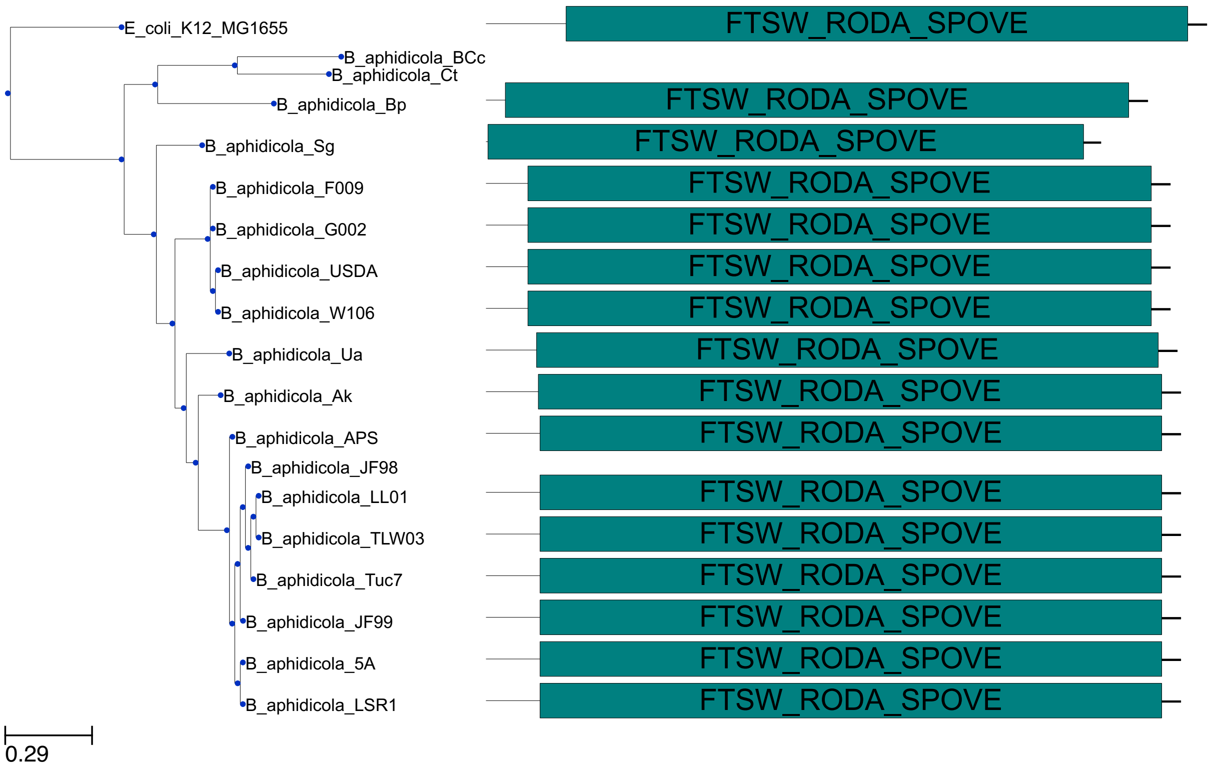

ftsW

NP_414631.1

putative lipid II flippase; integral membrane protein; FtsZ ring stabilizer

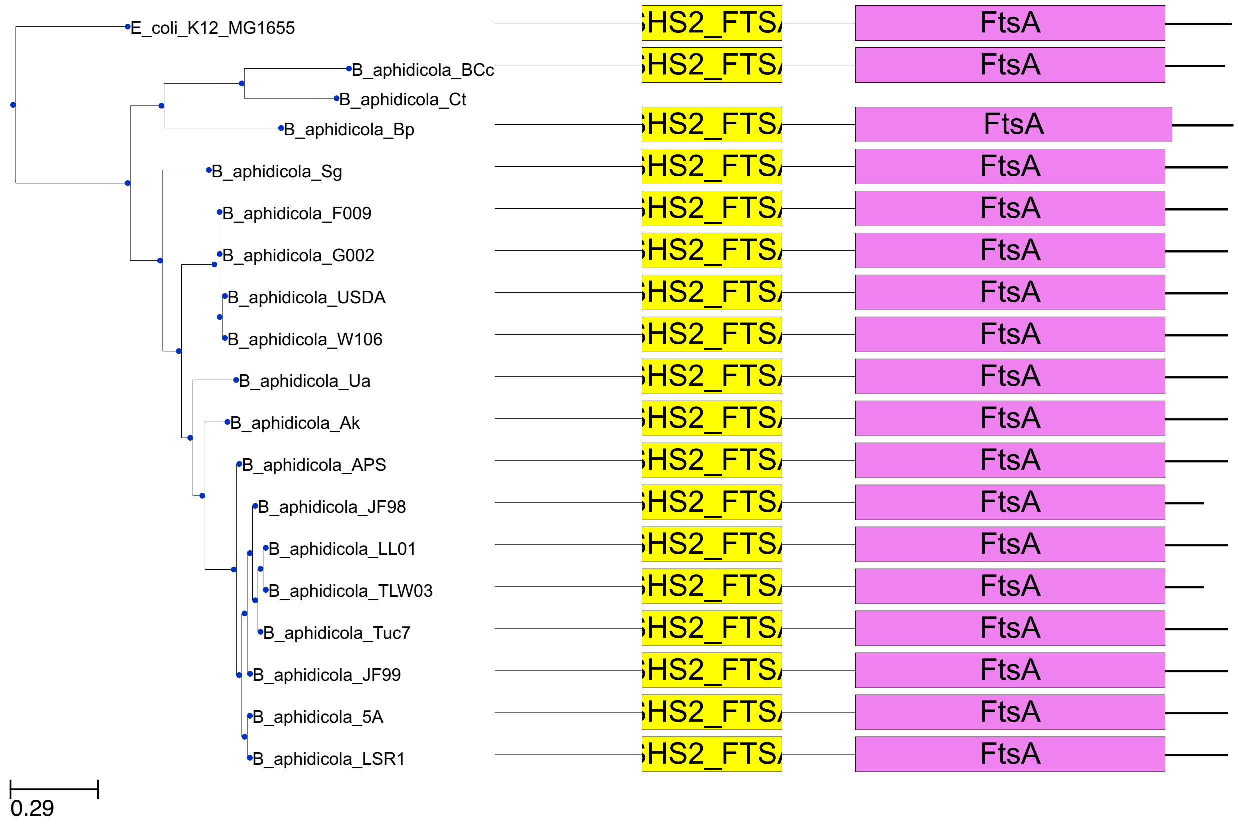

ftsA

NP_414636.1

ATP-binding cell division FtsK recruitment protein

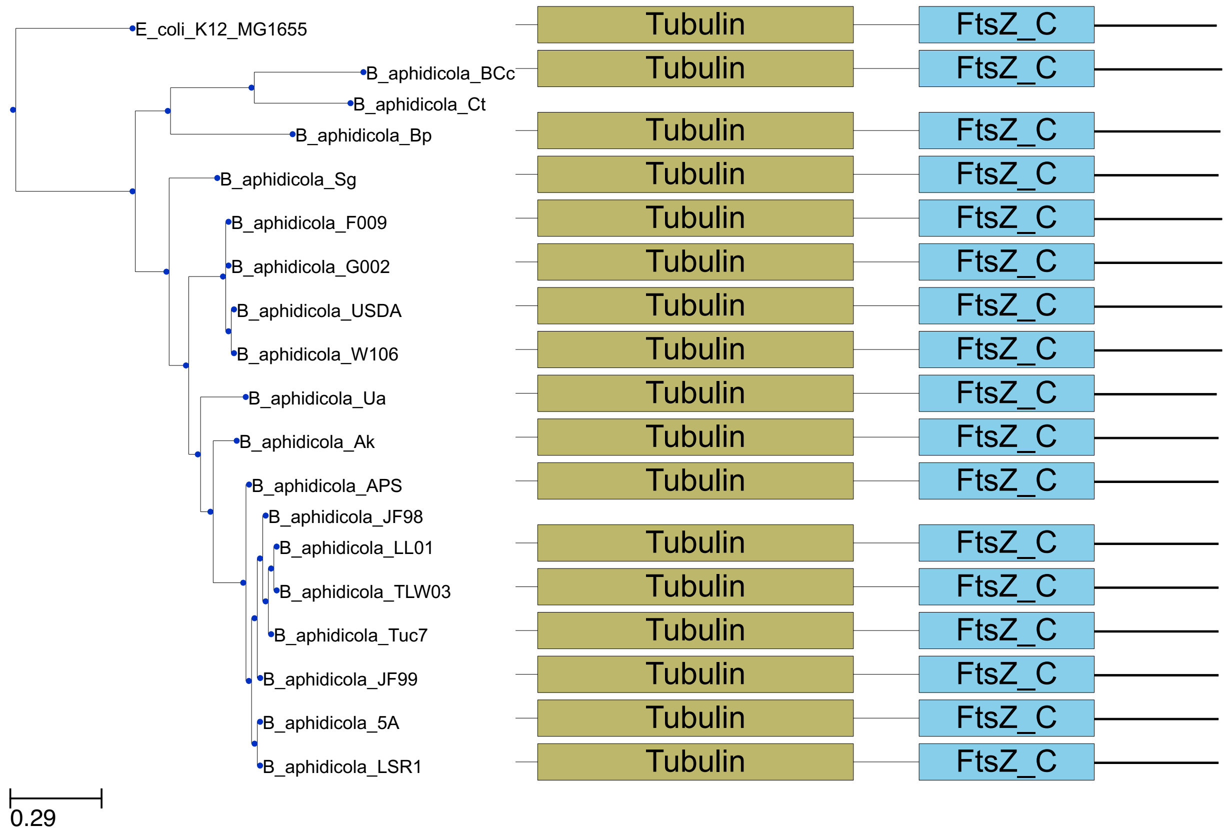

ftsZ
NP_414637.1
GTP-binding tubulin-like cell division protein

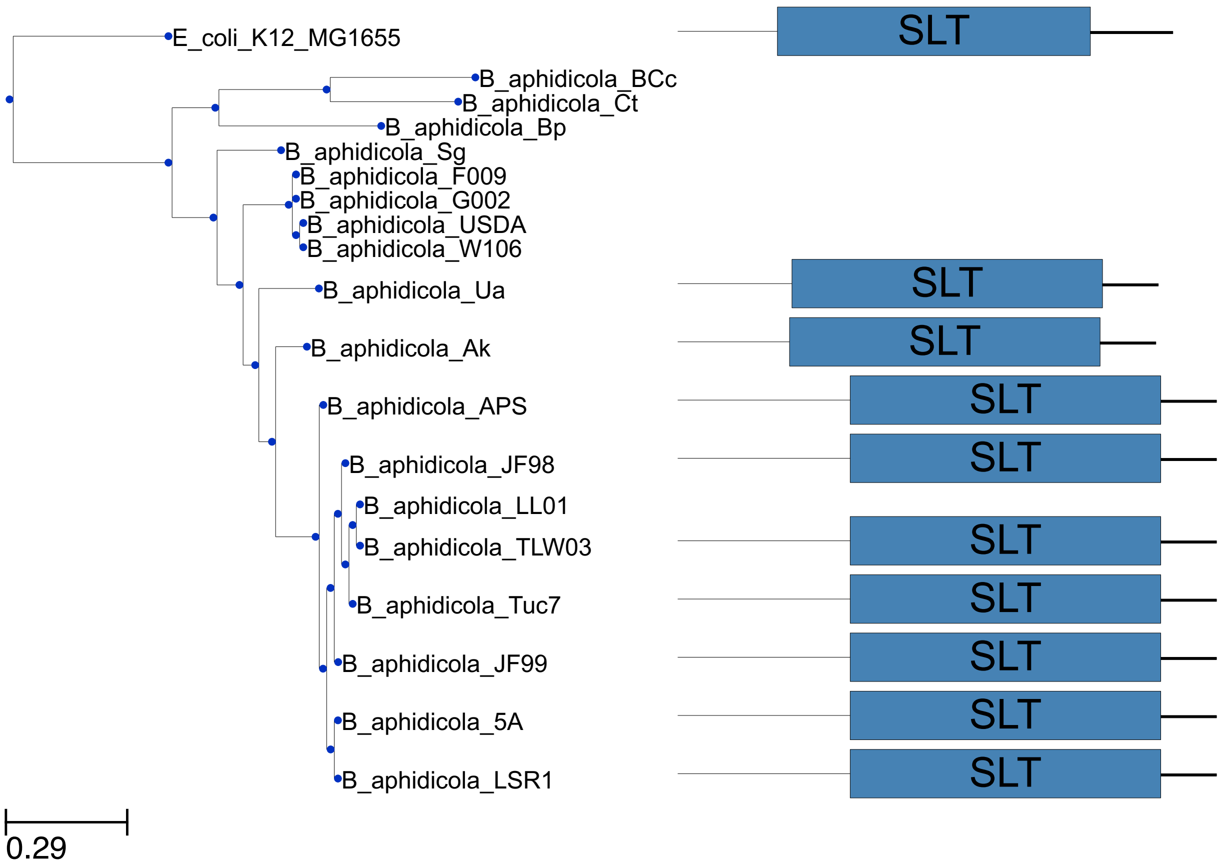

emtA

NP_415711.2

lytic murein endotransglycosylase E

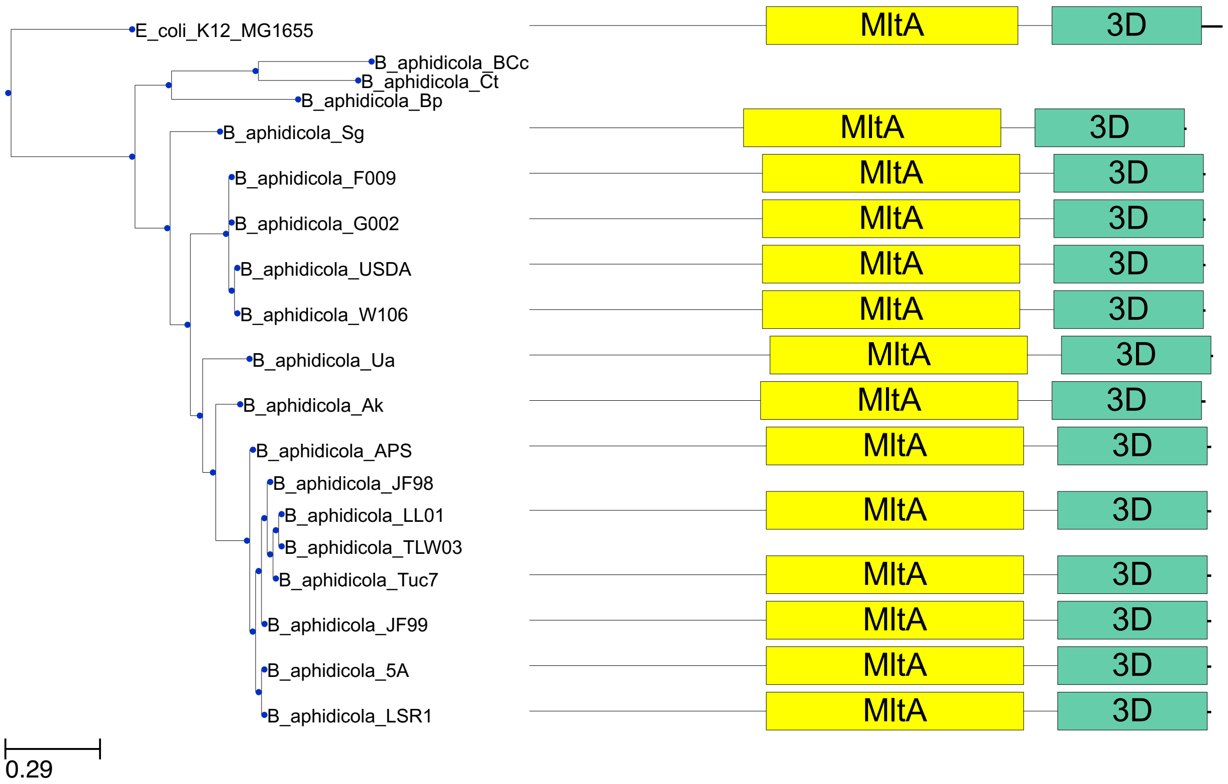

mltA
NP_417293.1
membrane-bound lytic murein transglycosylase A

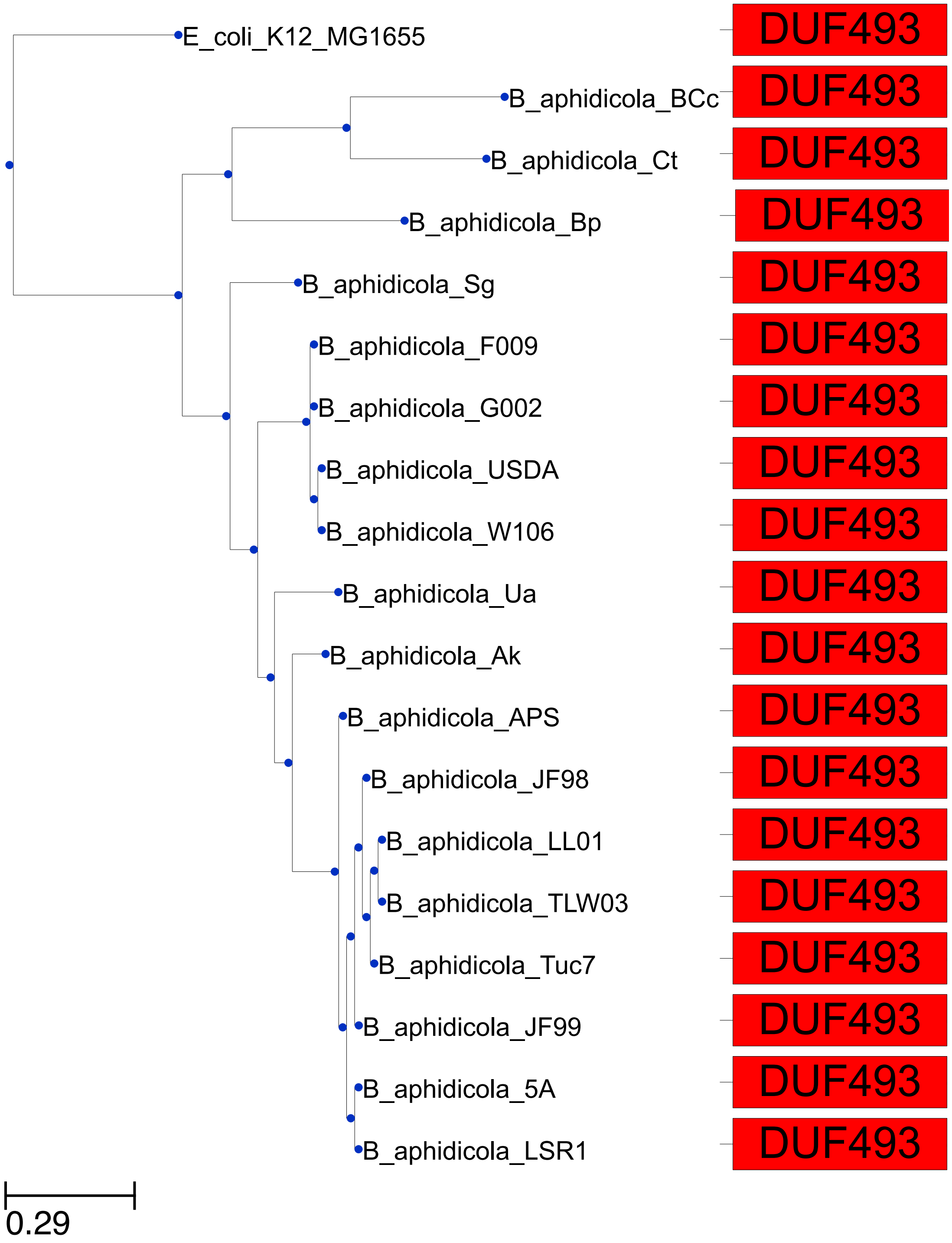

ybeD
NP_415164.1
UPF0250 family protein

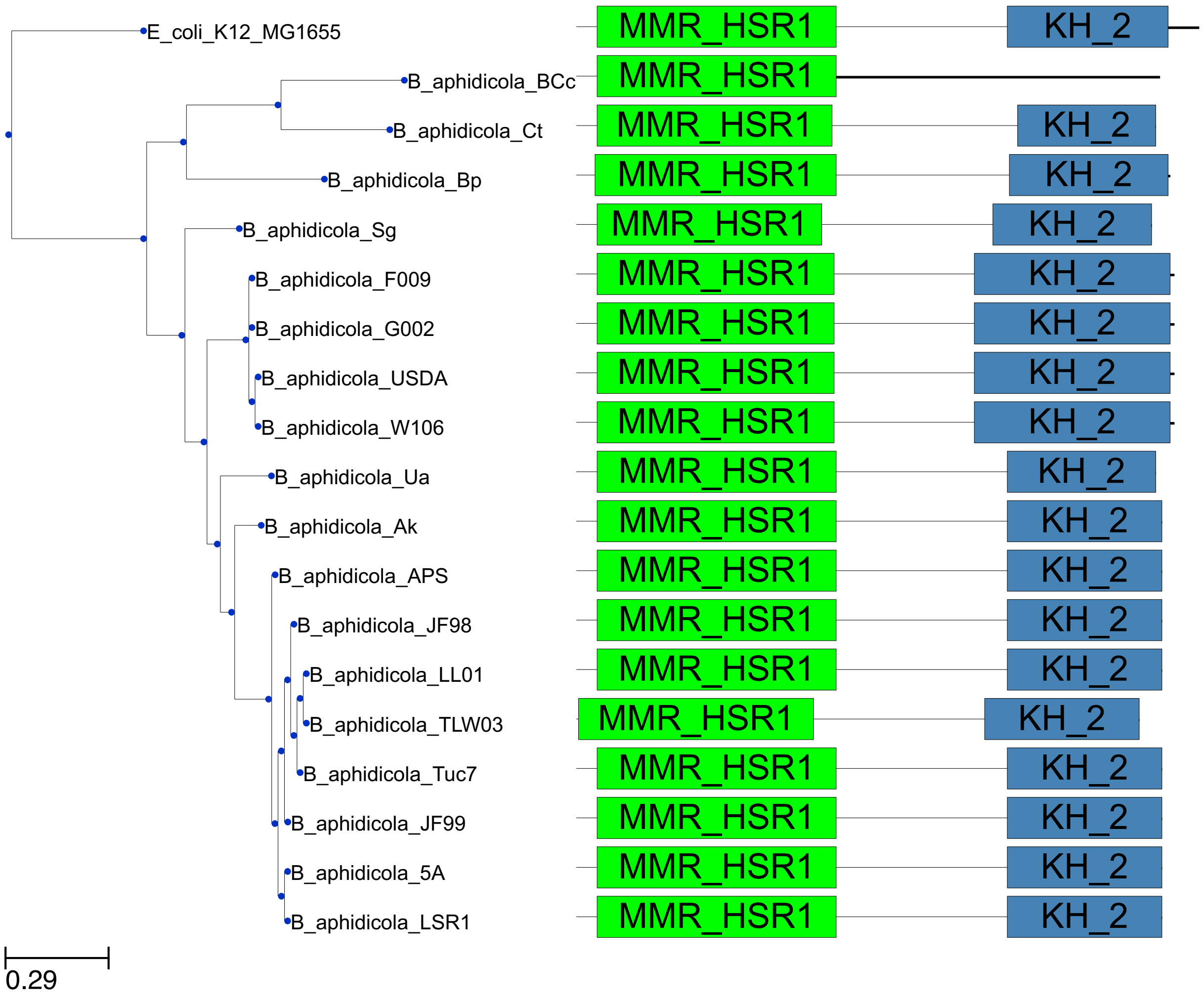

era
NP_417061.1
membrane-associated, 16S rRNA-binding GTPase

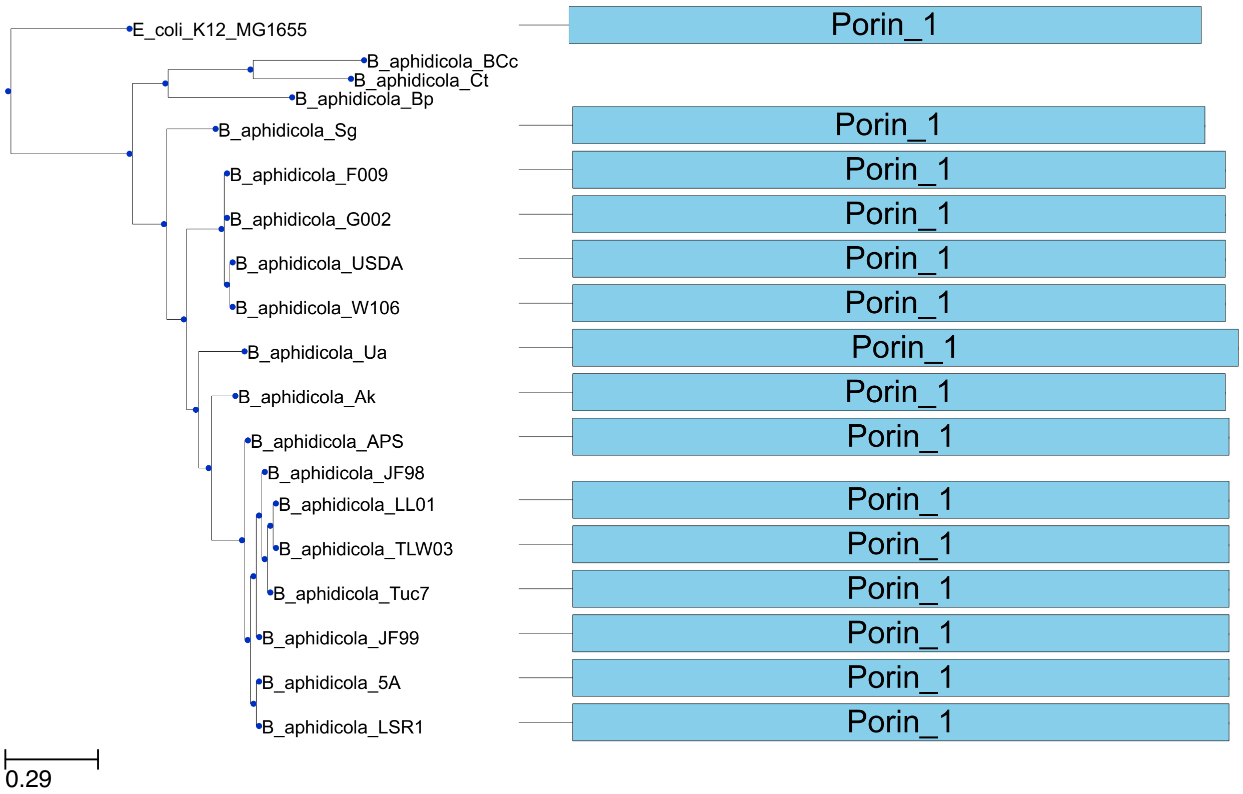

phoE
NP_414776.1
outer membrane phosphoporin protein E

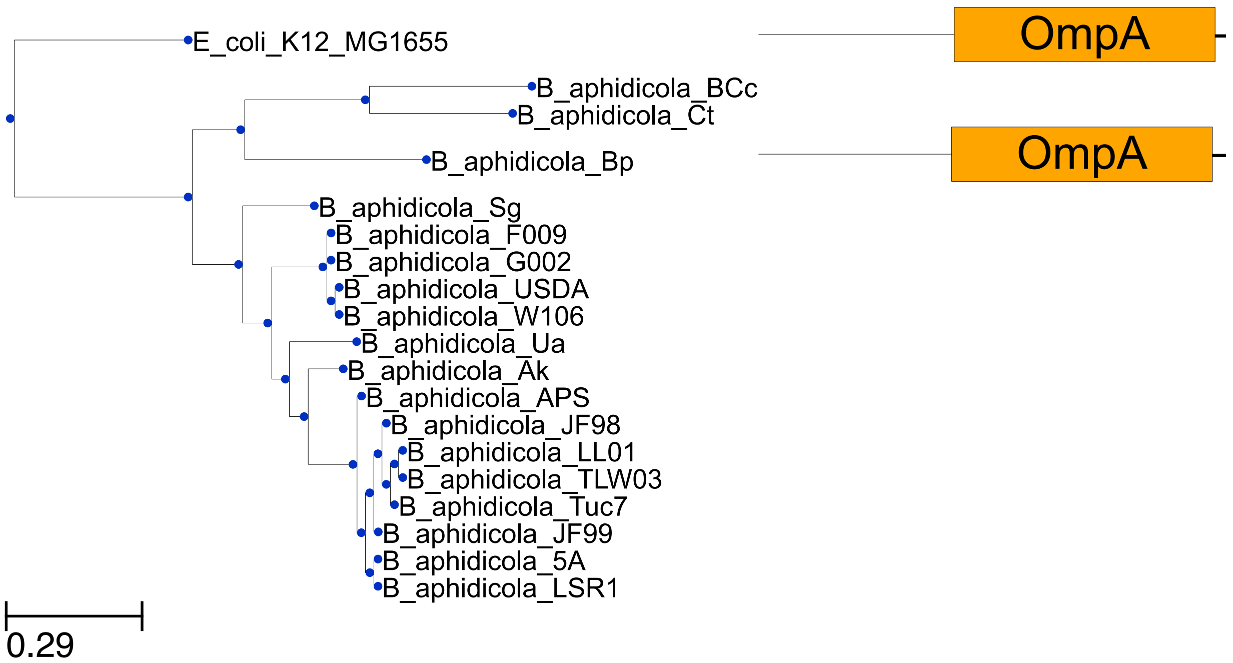

pal
NP_415269.1
peptidoglycan-associated outer membrane lipoprotein

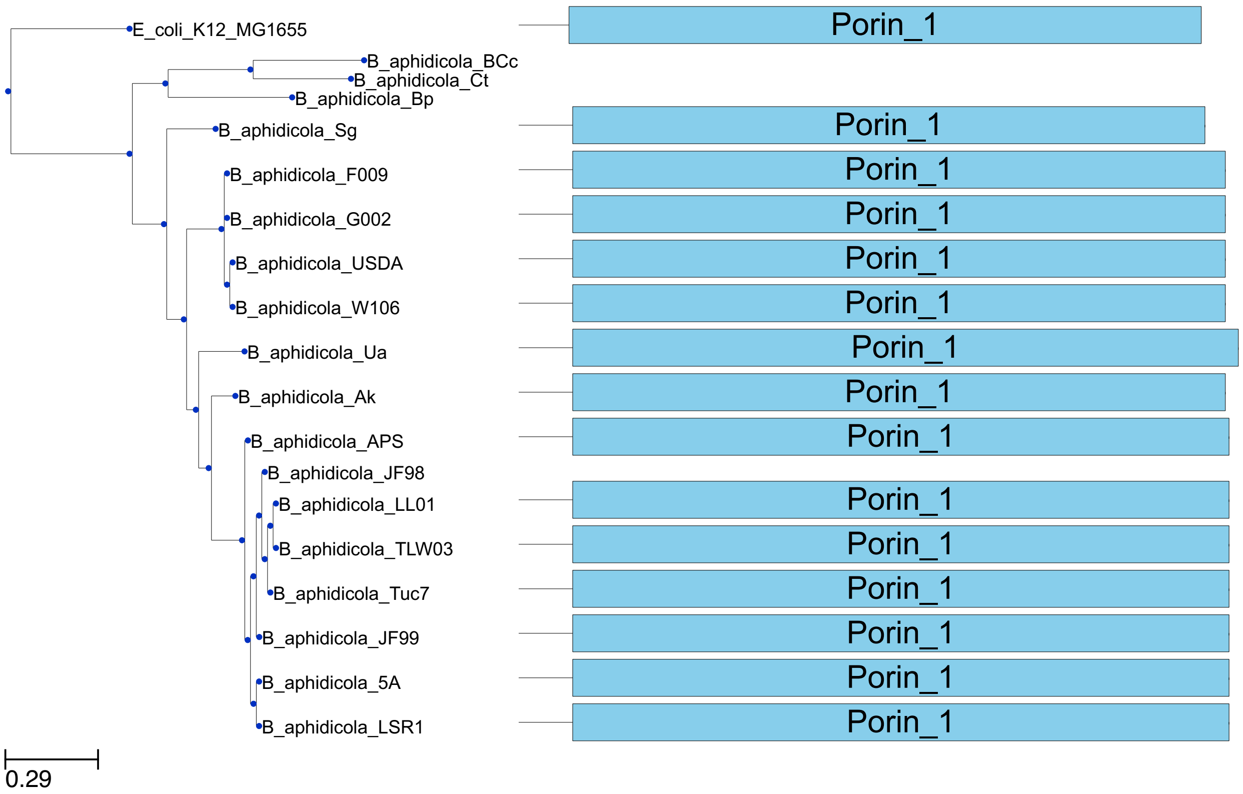

ompF
NP_415449.1
outer membrane porin 1a (Ia;b;F)

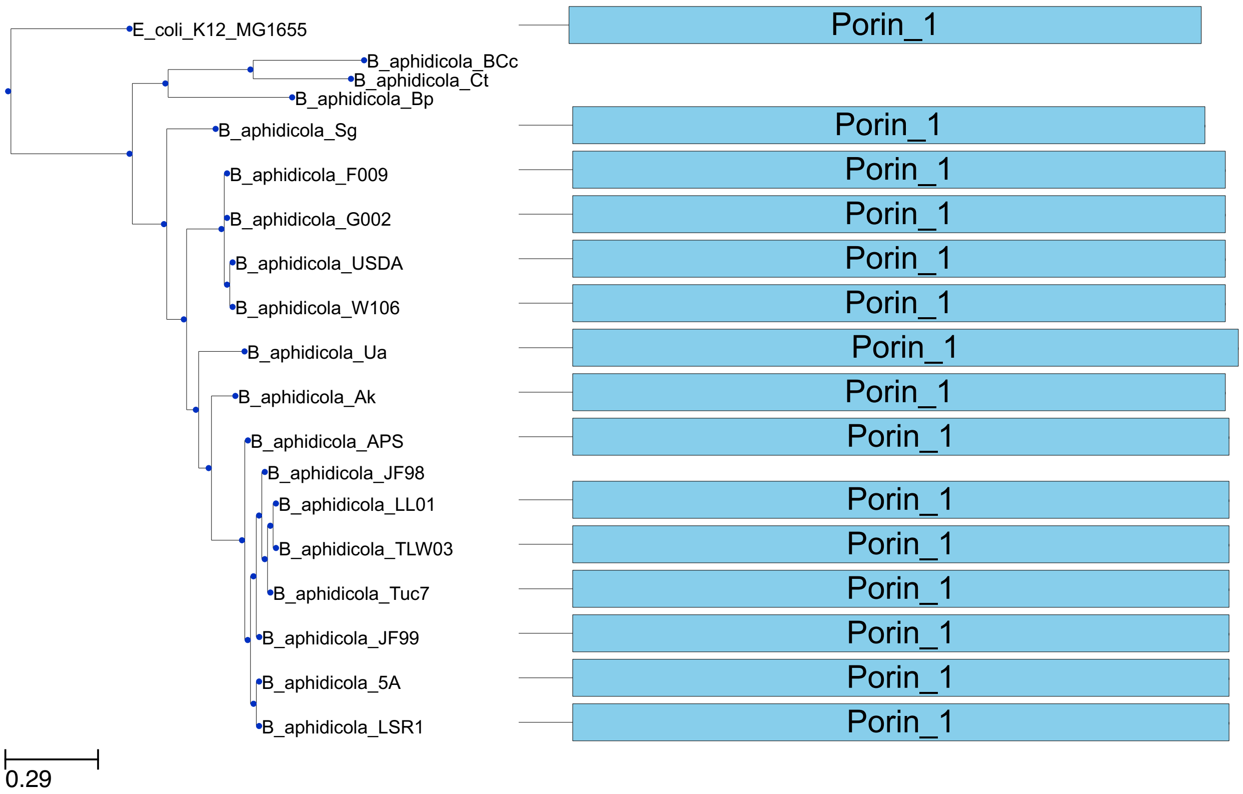

ompC
NP_416719.1
outer membrane porin protein C

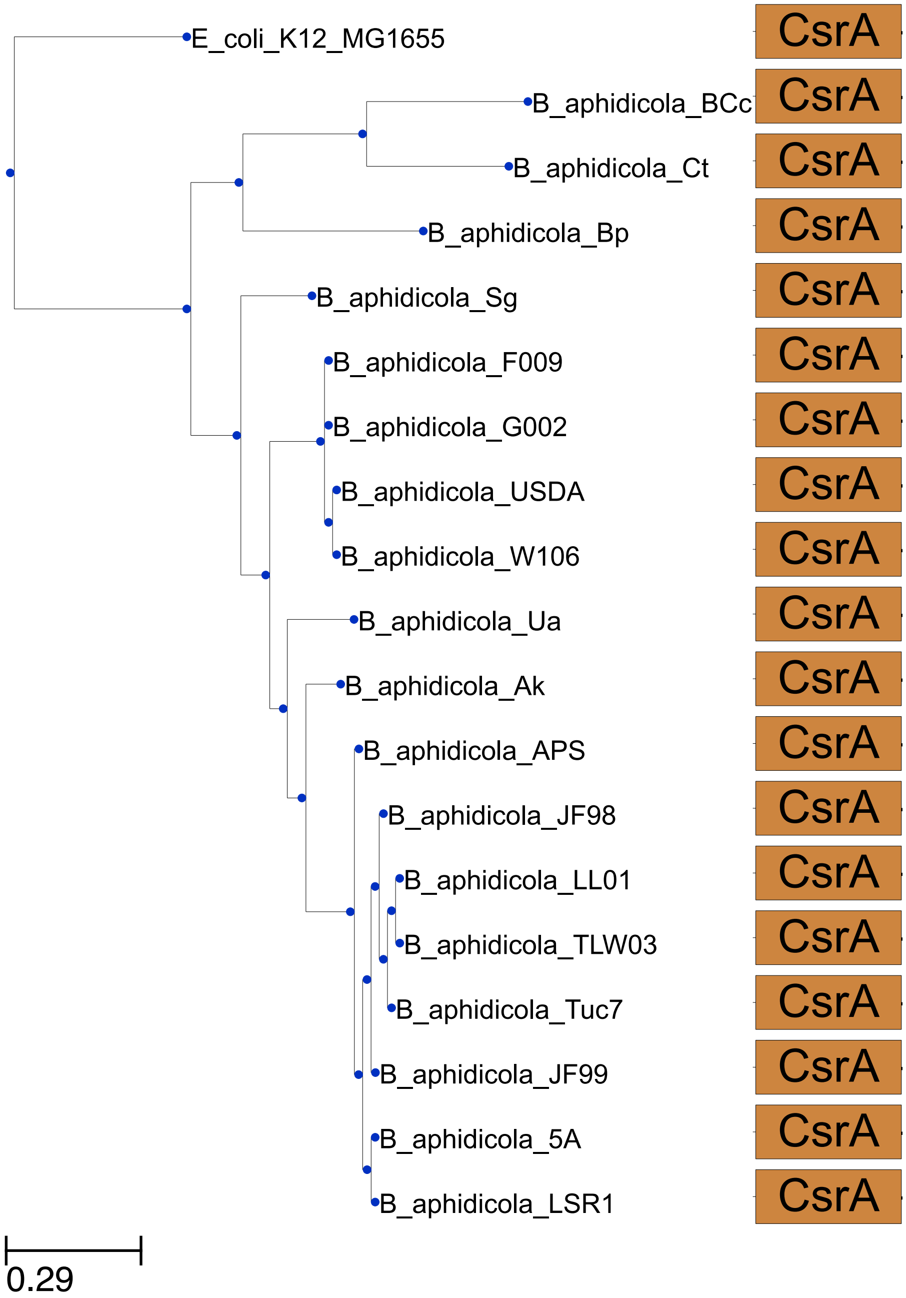

csrA
NP_417176.1
pleiotropic regulatory protein for carbon source metabolism

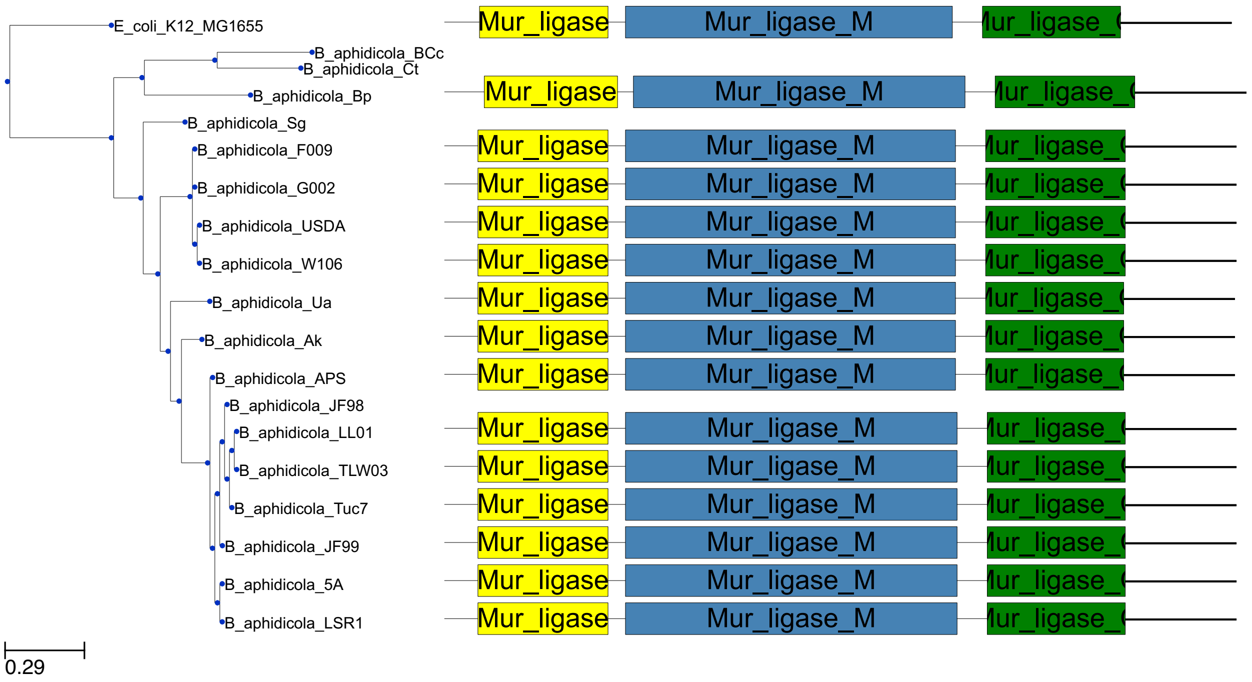

murE

NP_414627.1

UDP-N-acetylmuramoyl-L-alanyl-D-glutamate:meso- diaminopimelate ligase

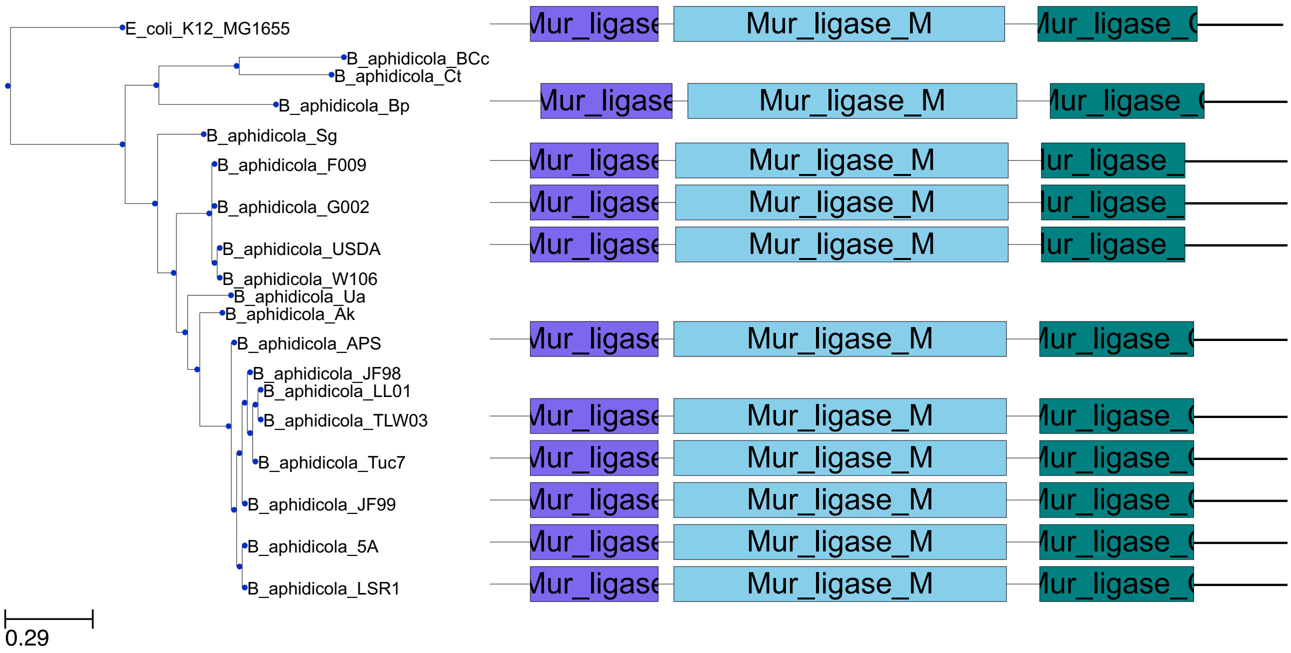

murF
NP_414628.1
UDP-N-acetylmuramoyl-tripeptide:D-alanyl-D- alanine ligase

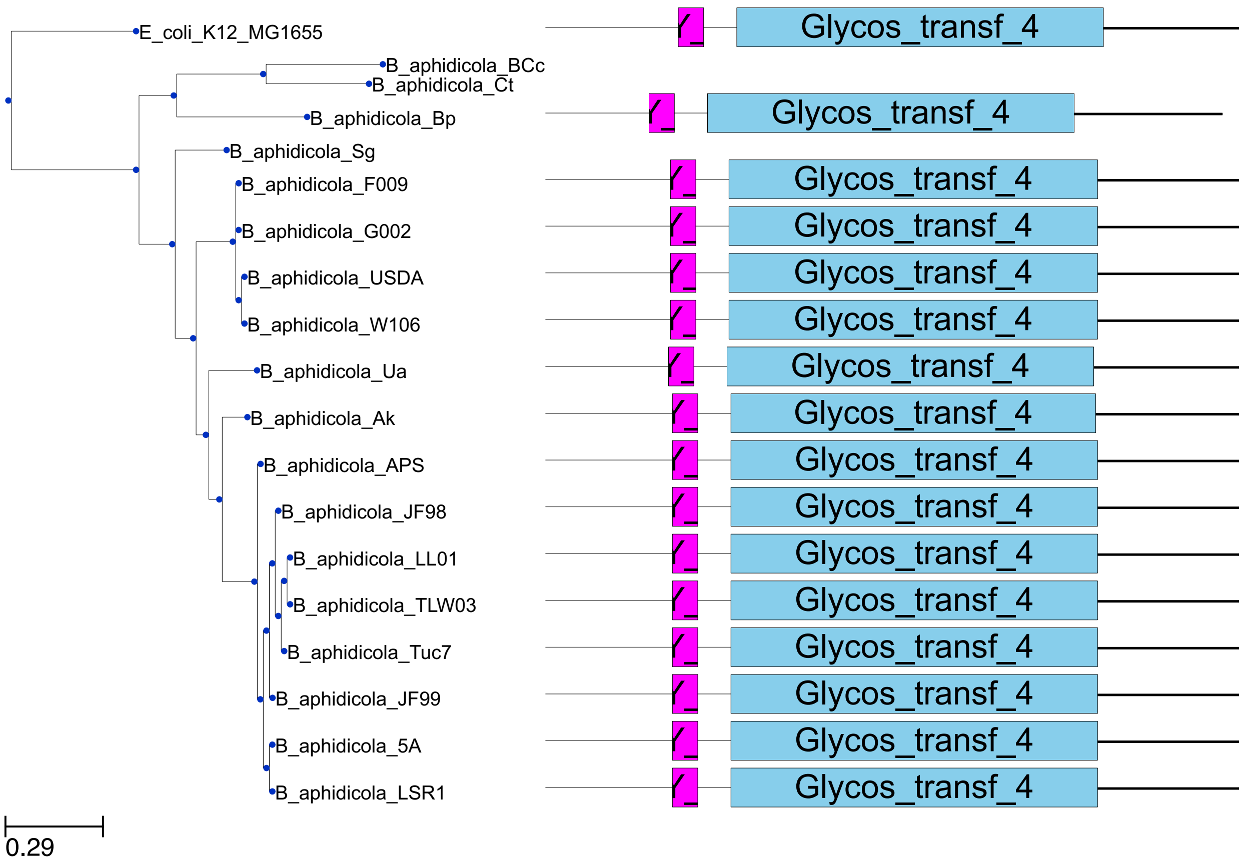

mraY

NP_414629.1

phospho-N-acetylmuramoyl-pentapeptide transferase

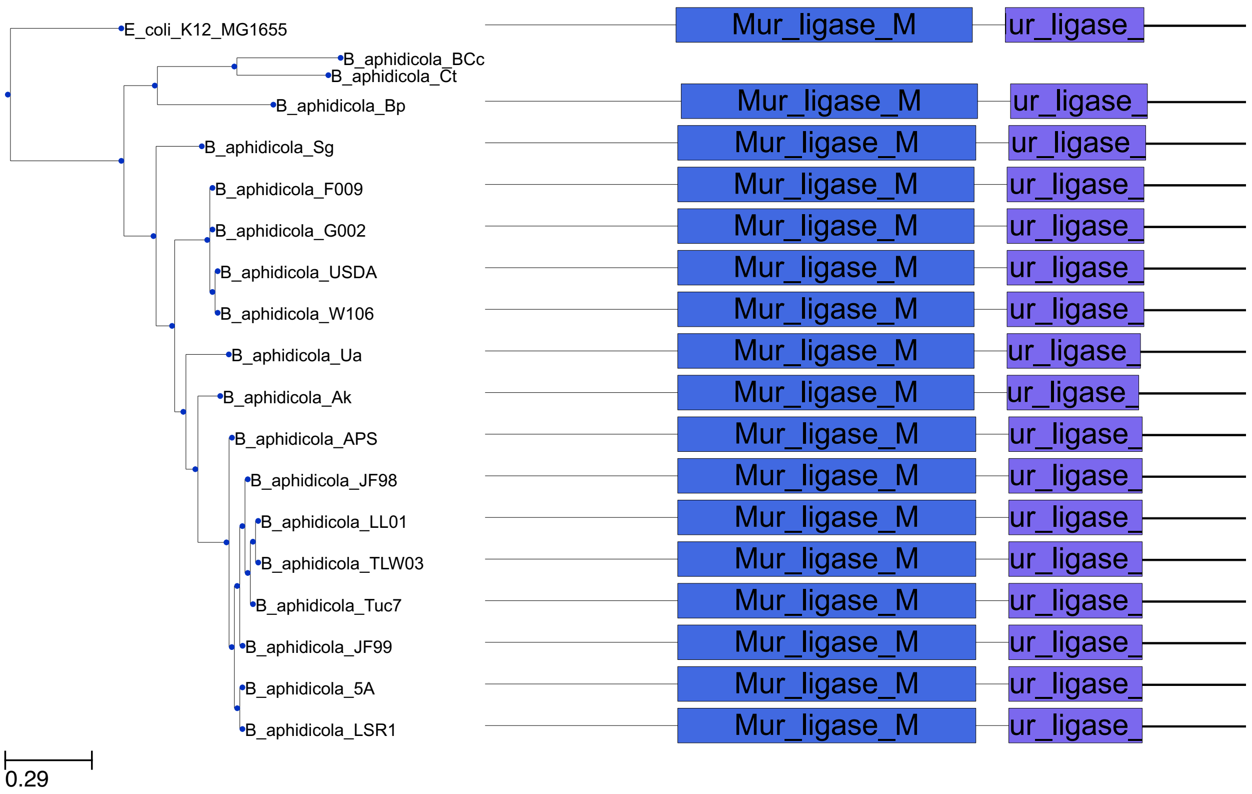

murD

NP_414630.1

UDP-N-acetylmuramoyl-L-alanine:D-glutamate ligase

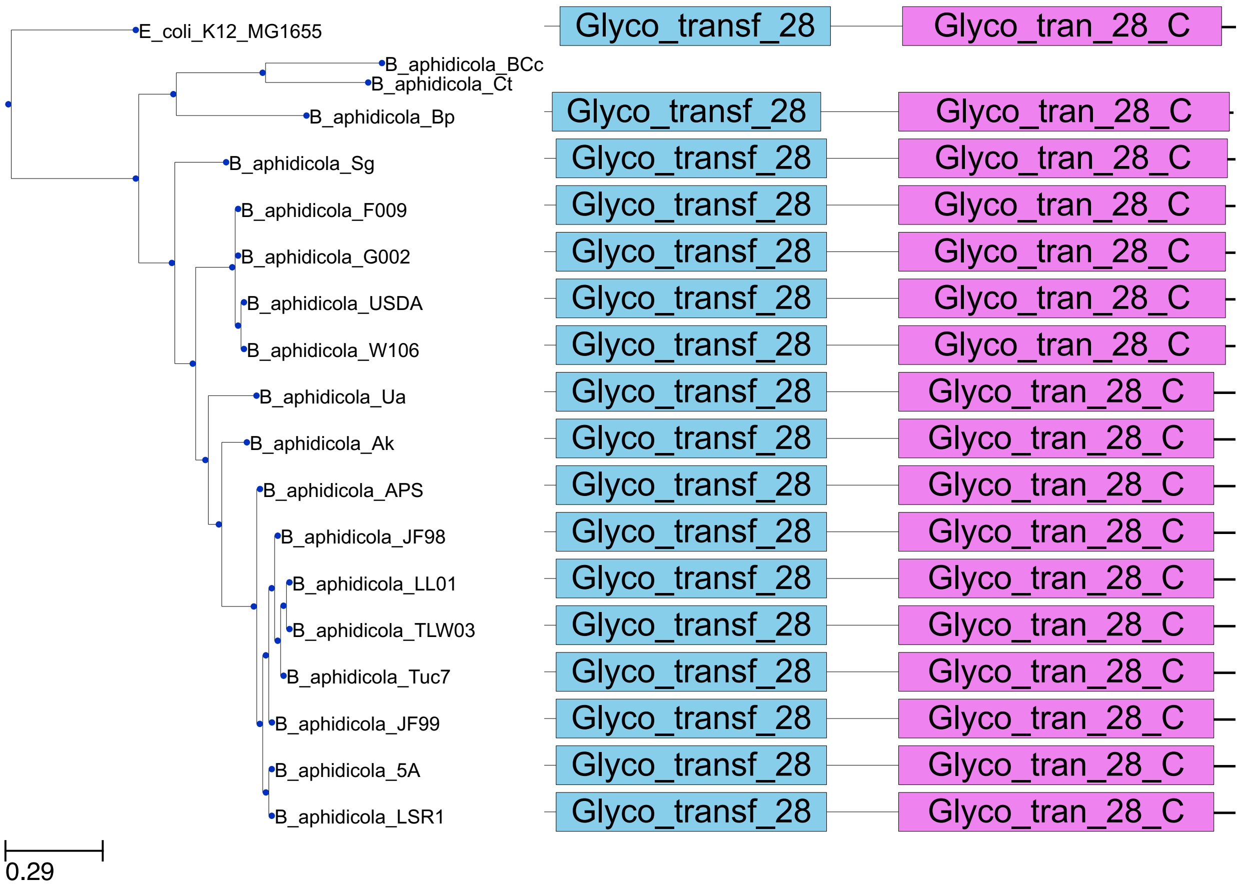

murG
NP_414632.1
N-acetylglucosaminyl transferase

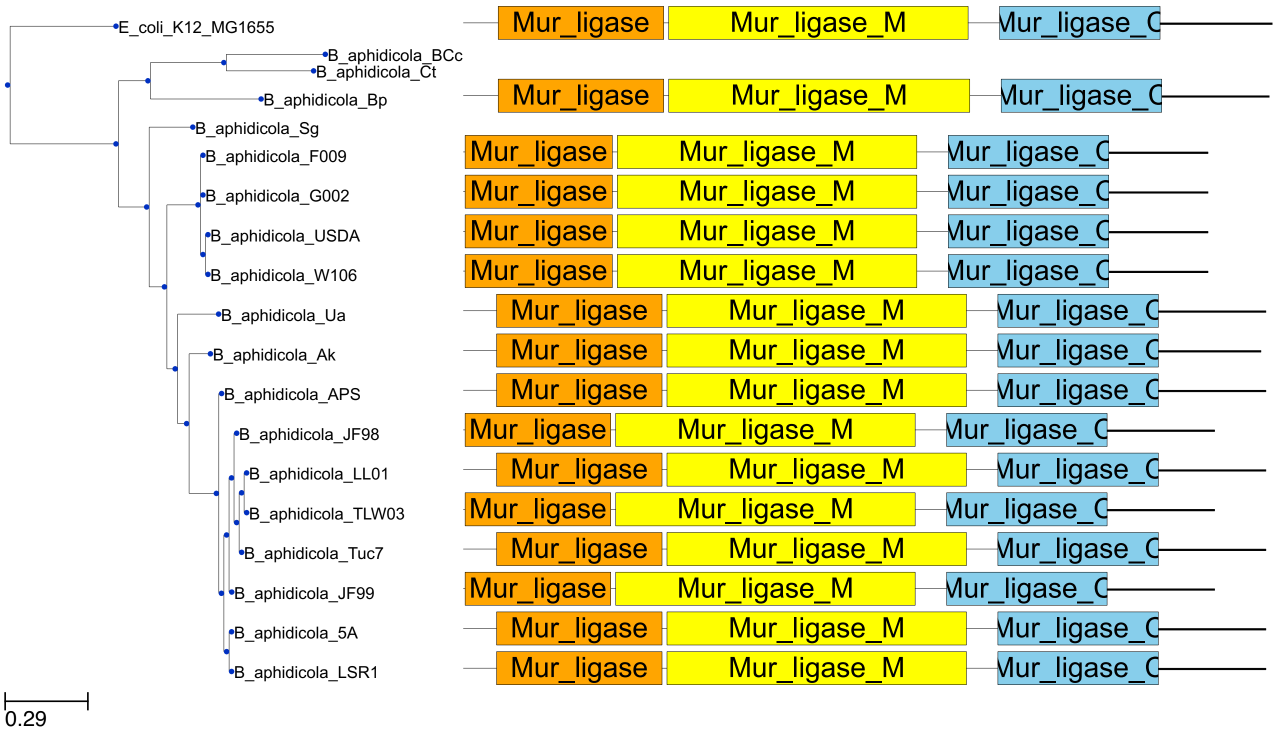

murC
NP_414633.1
UDP-N-acetylmuramate:L-alanine ligase

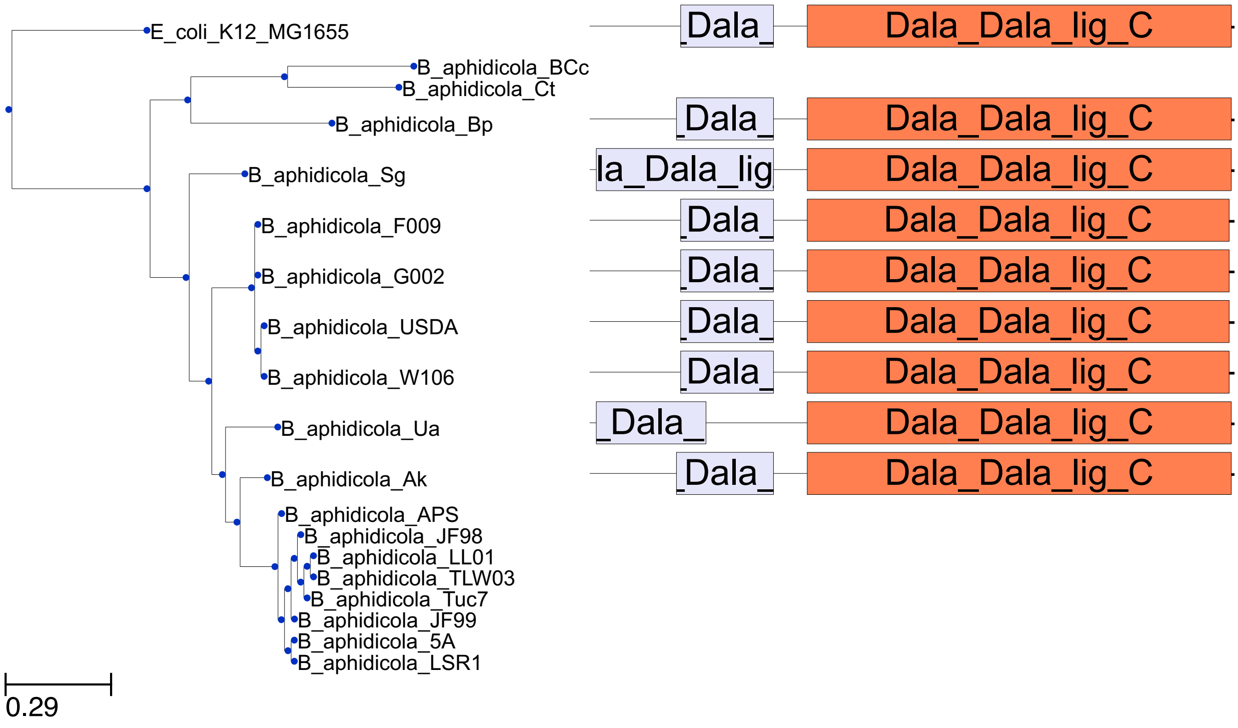

ddlB
NP_414634.1
D-alanine:D-alanine ligase

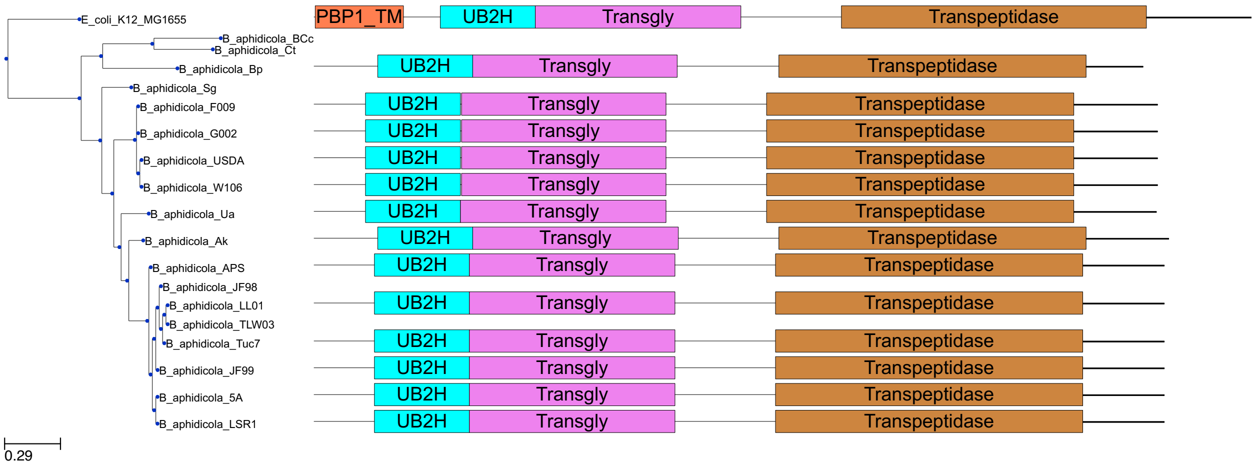

mrcB

NP_414691.1

fused glycosyl transferase and transpeptidase

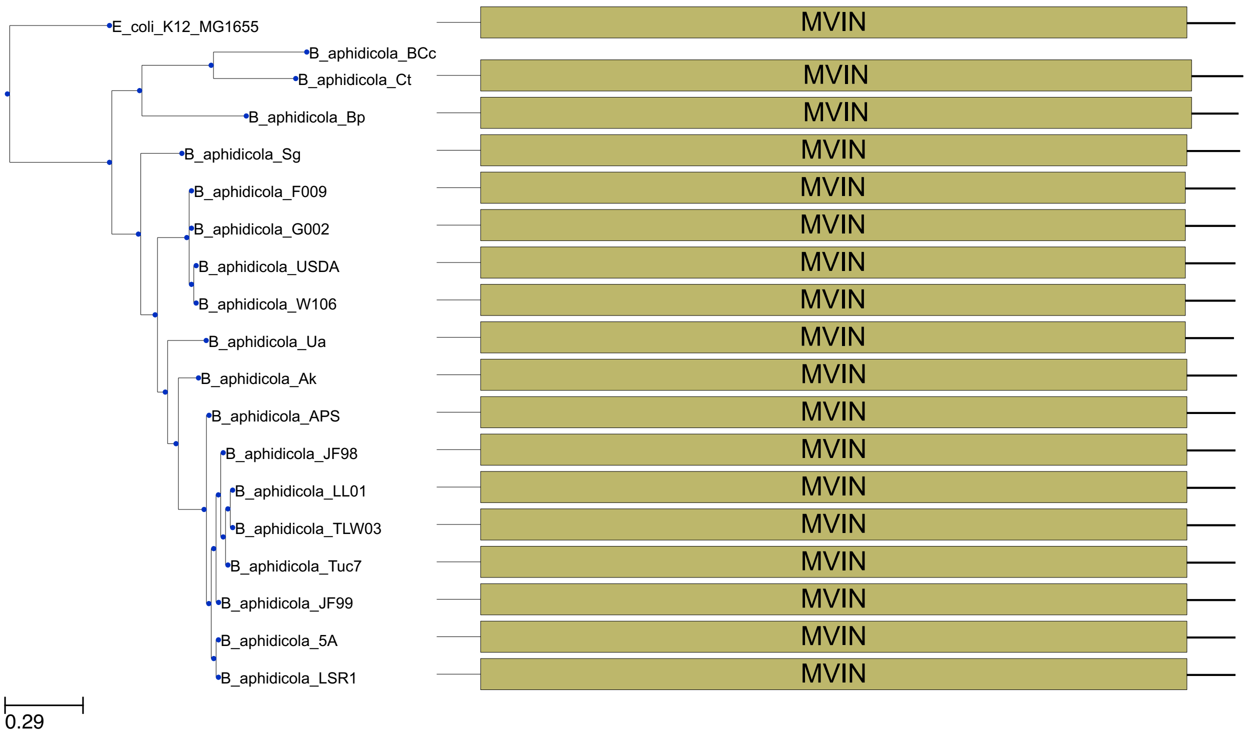

mviN

NP_415587.1

putative lipid II flippase

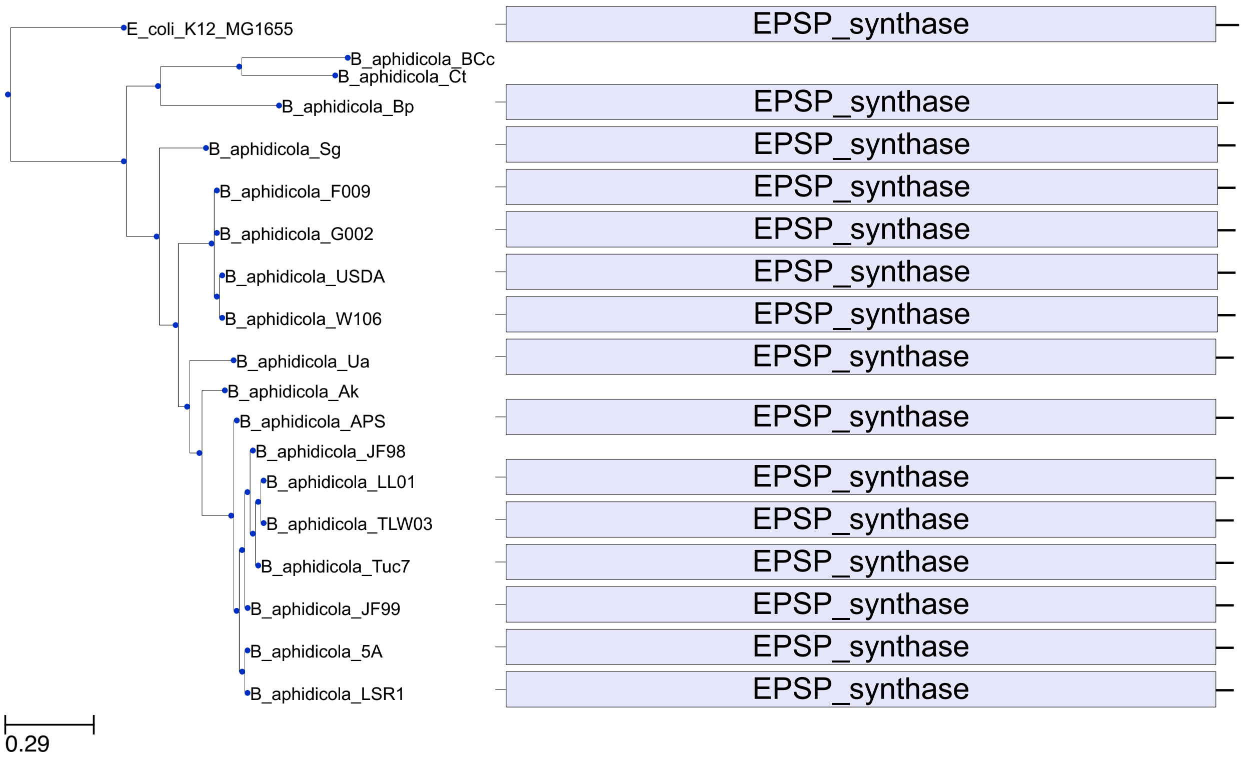

murA

NP_417656.1

UDP-N-acetylglucosamine 1-carboxyvinyltransferase

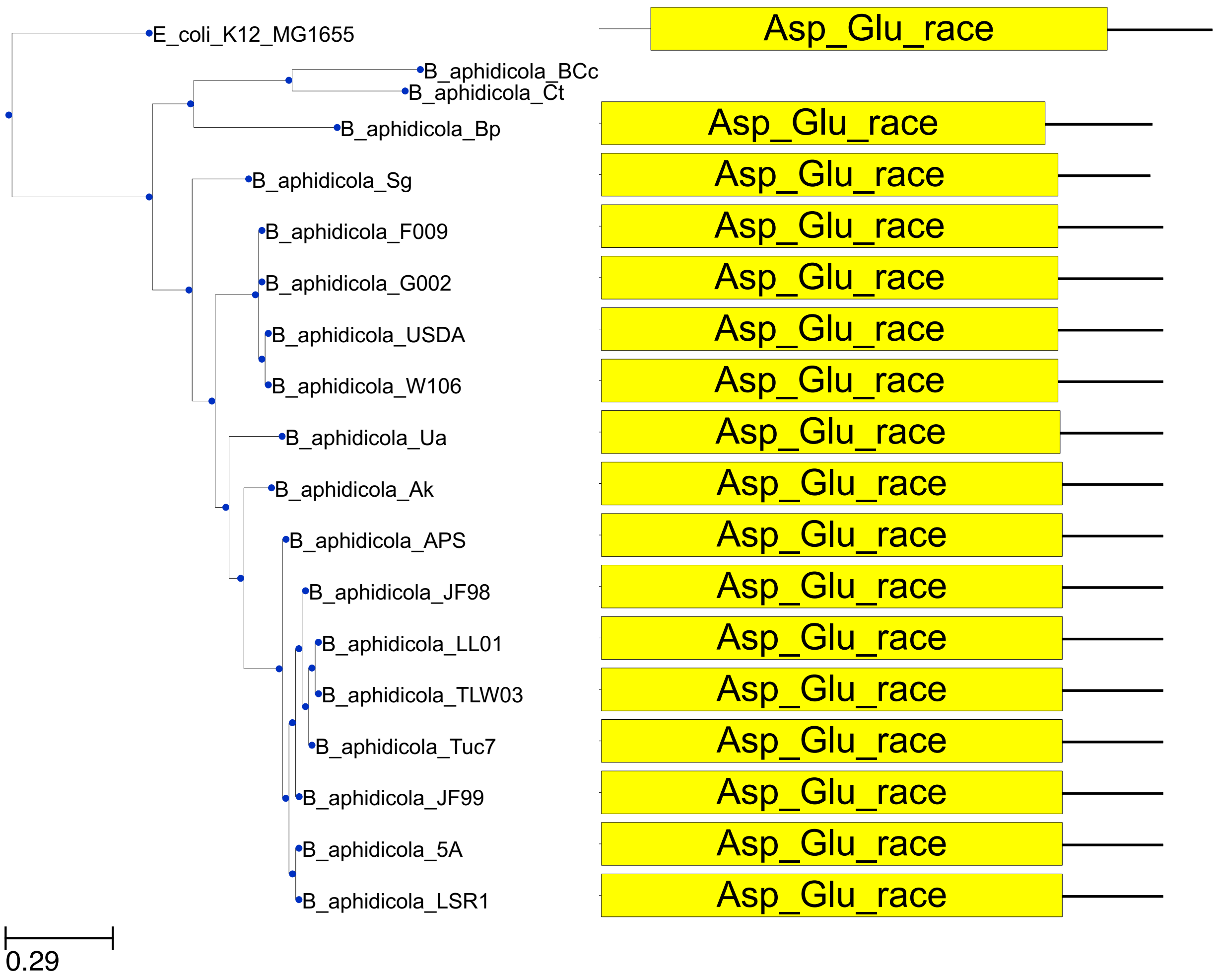

murI
NP_418402.2
glutamate racemase

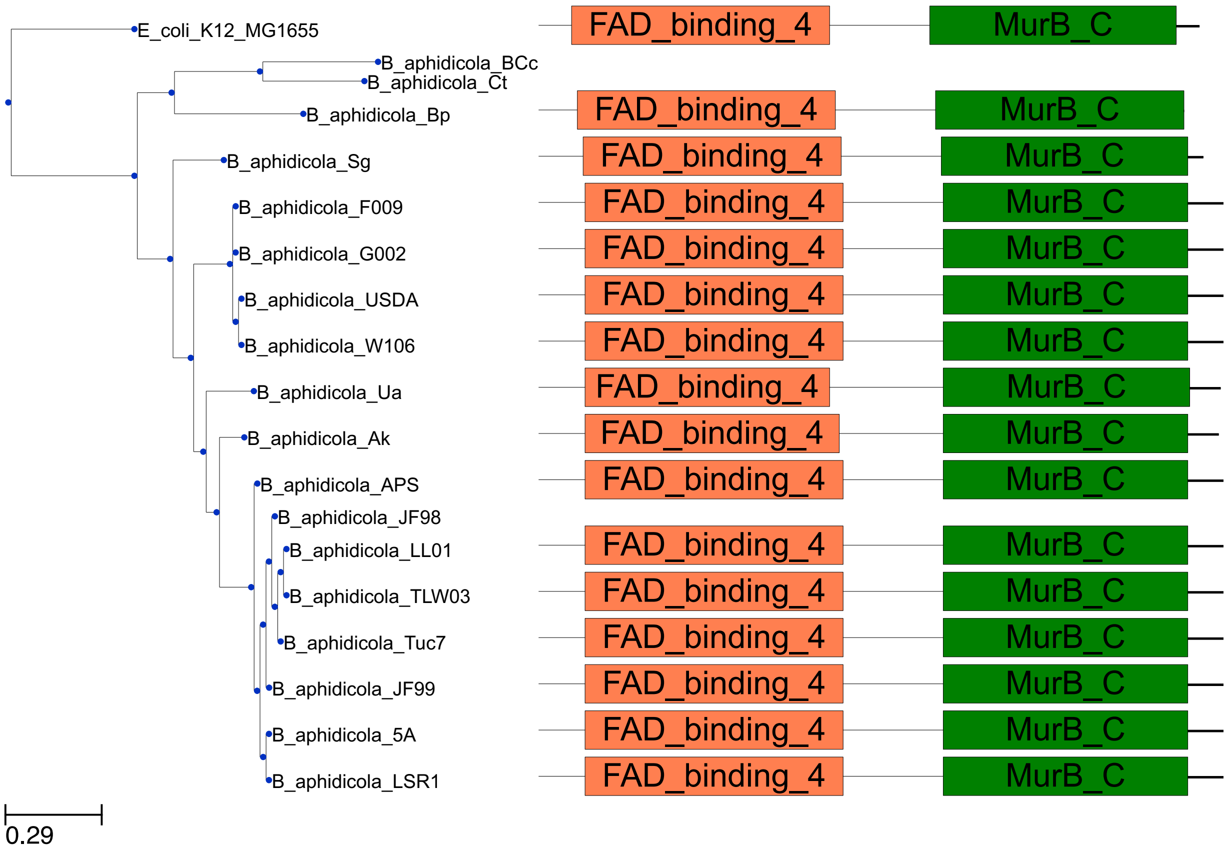

murB

NP_418403.1

"UDP-N-acetylenolpyruvoylglucosamine reductase, FAD-binding"

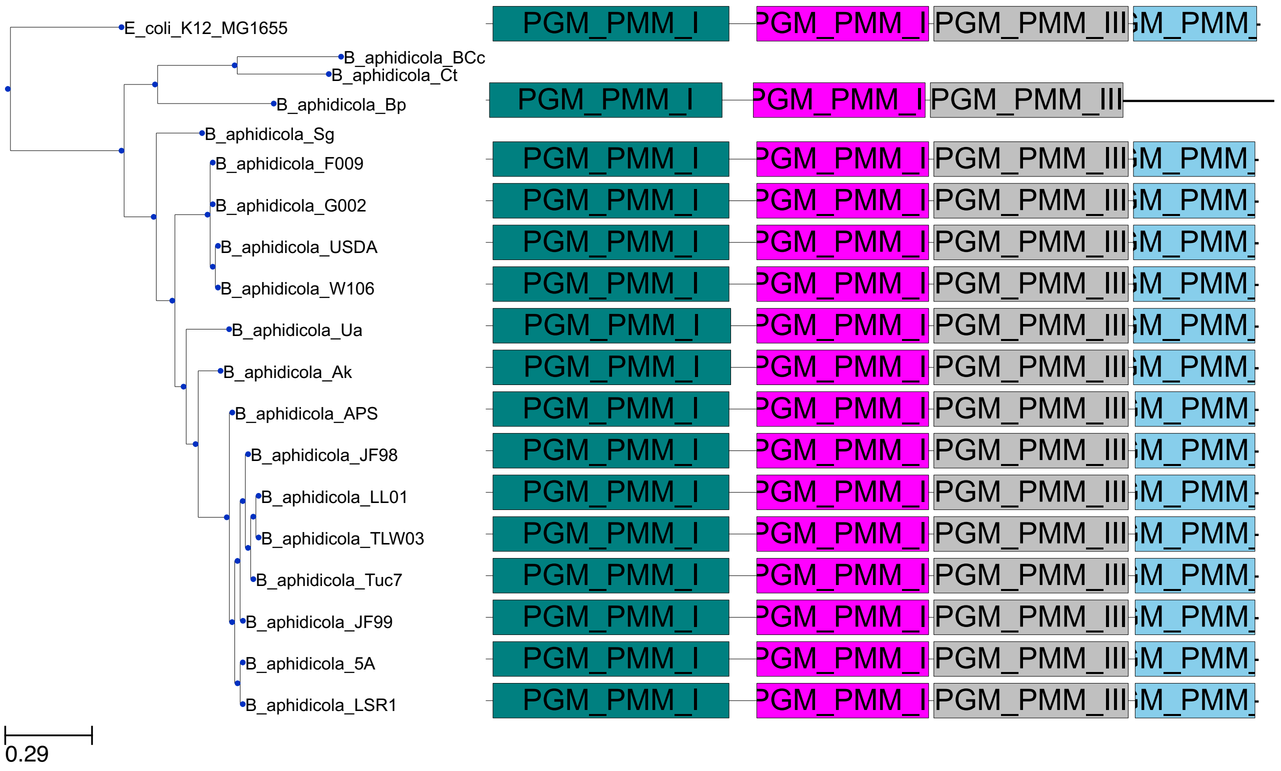

glmM
NP_417643.1
phosphoglucosamine mutase

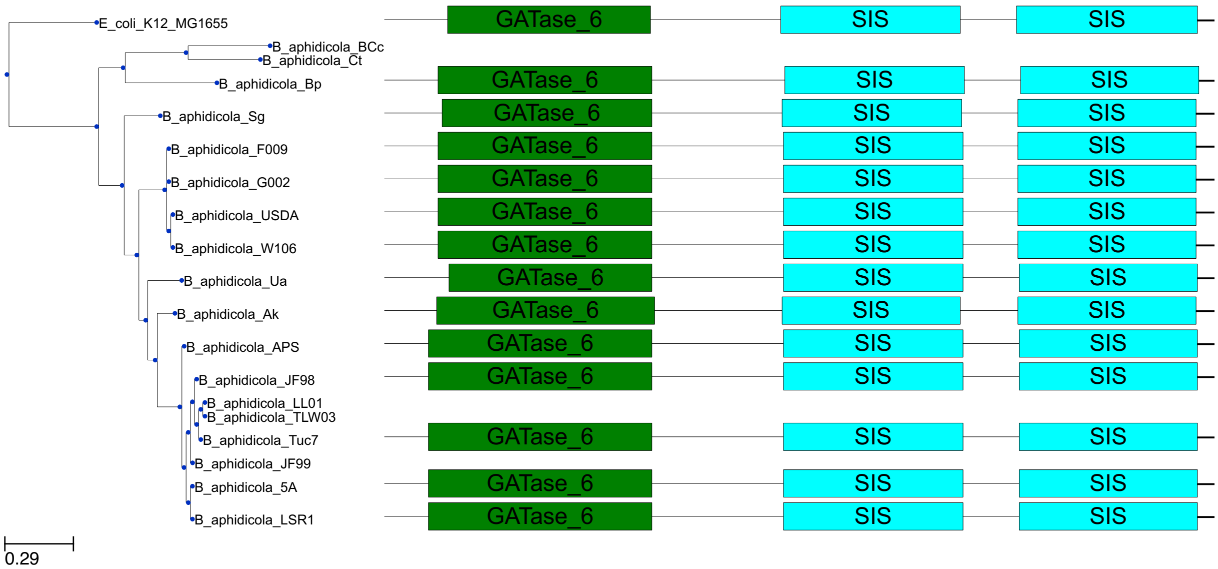

glmS

NP_418185.1

L-glutamine:D-fructose-6-phosphate aminotransferase

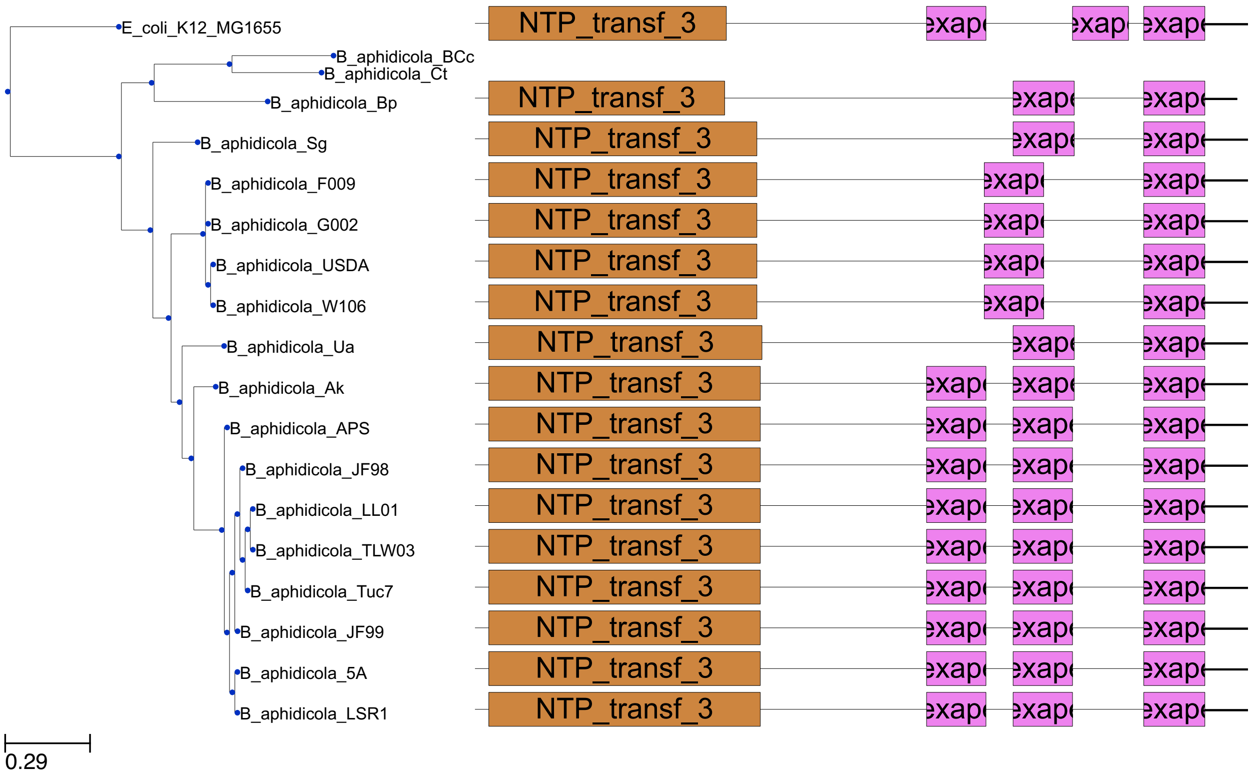

glmU

NP_418186.1

fused N-acetyl glucosamine-1-phosphate uridyltransferase/glucosamine-1-phosphate acetyl transferase

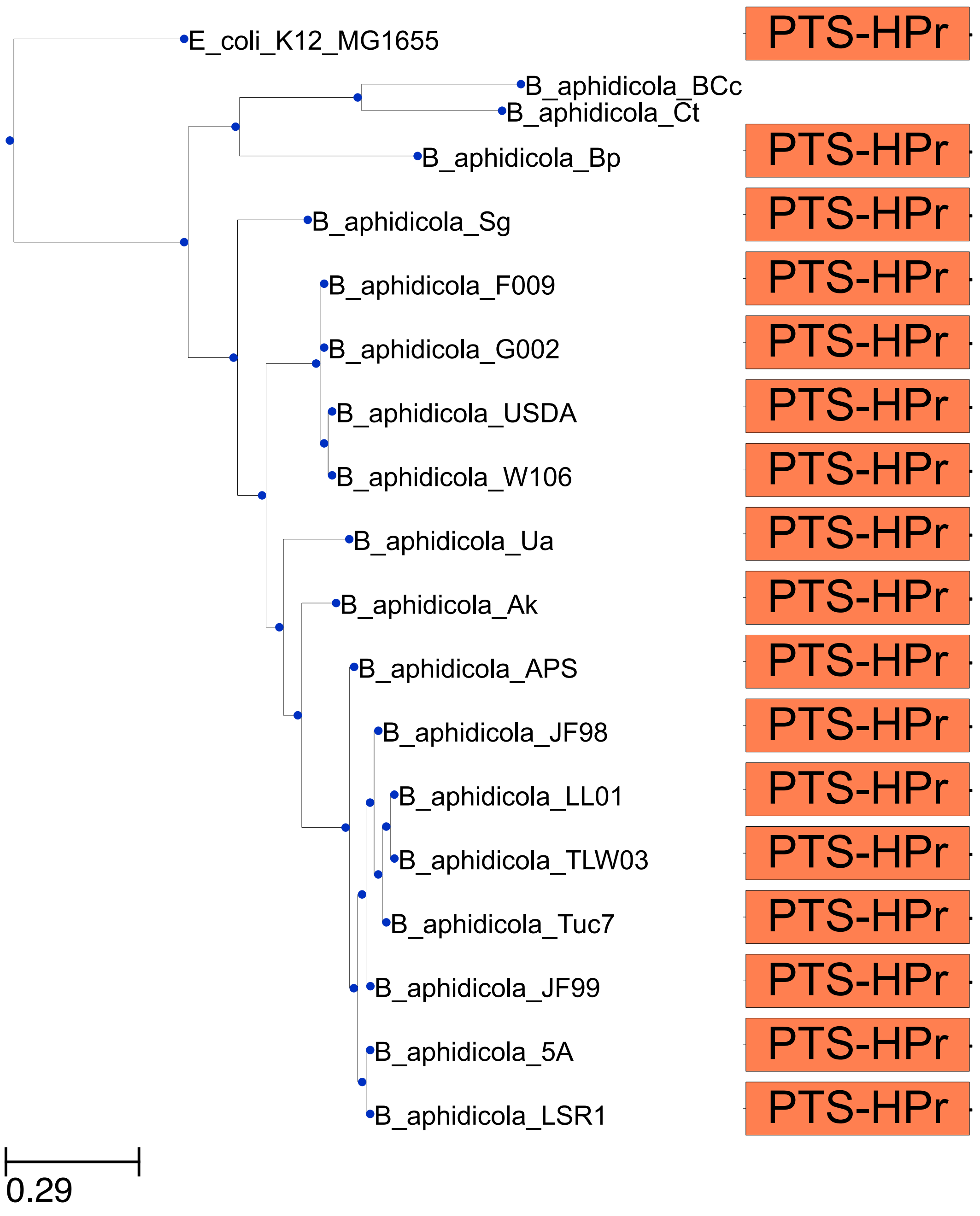

ptsH
NP_416910.1
phosphohistidinoprotein-hexose phosphotransferase component of PTS system (Hpr)

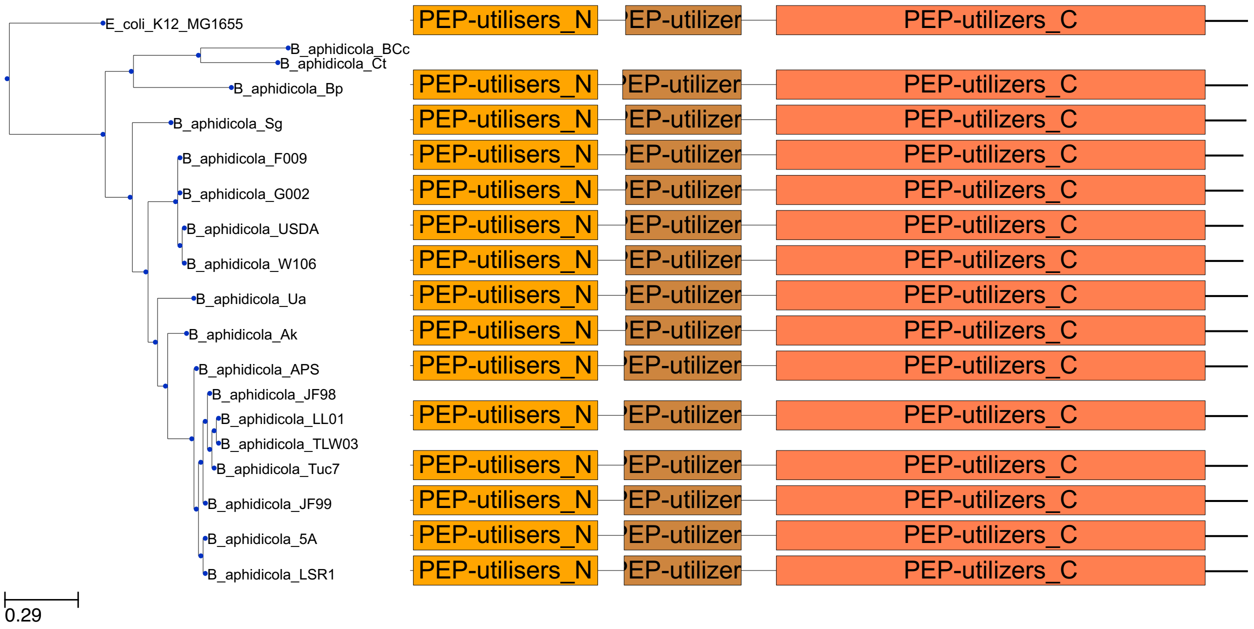

ptsI
NP_416911.1
PEP-protein phosphotransferase of PTS system (enzyme I)

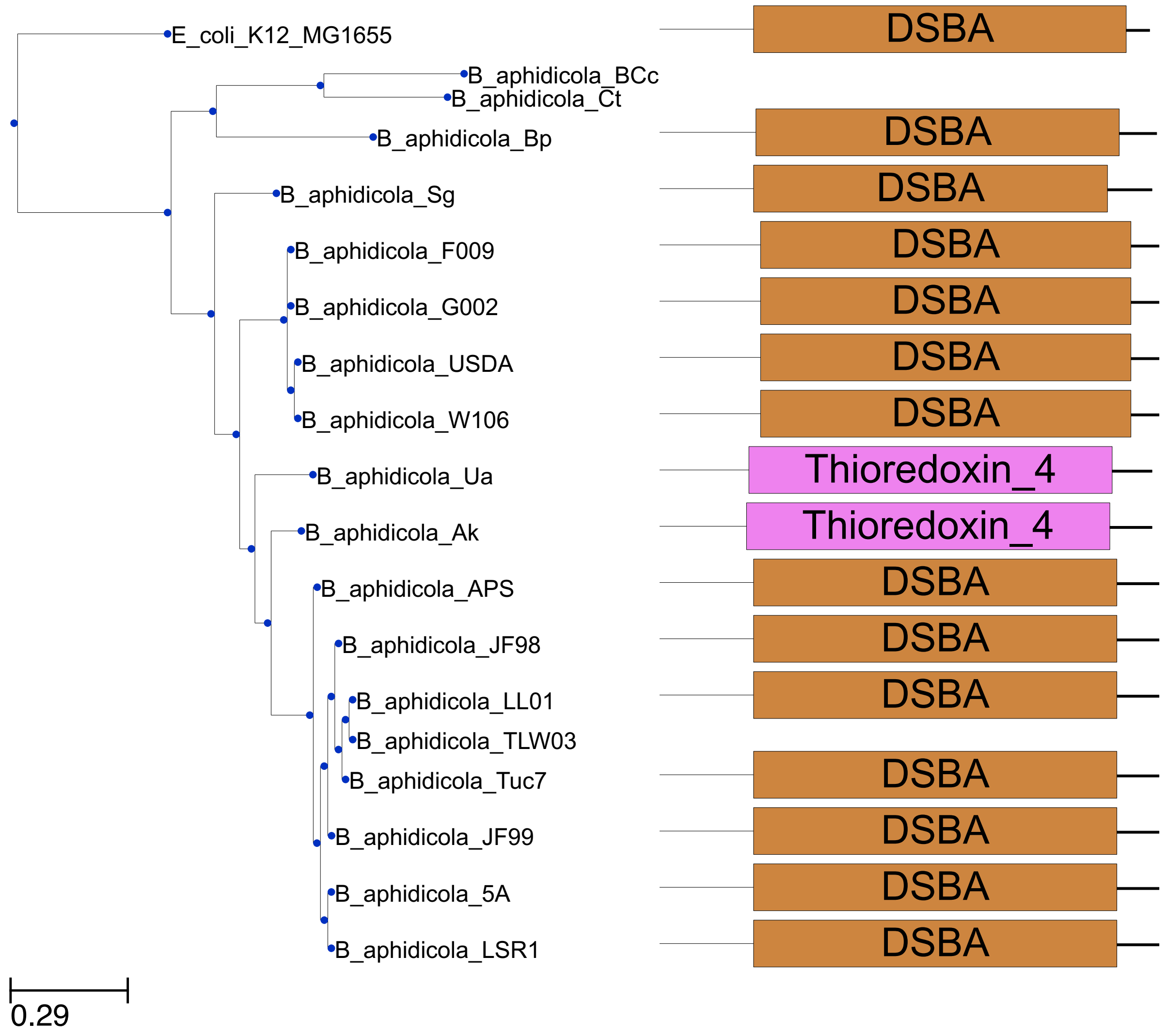

dsbA
NP_418297.1
periplasmic protein disulfide isomerase I

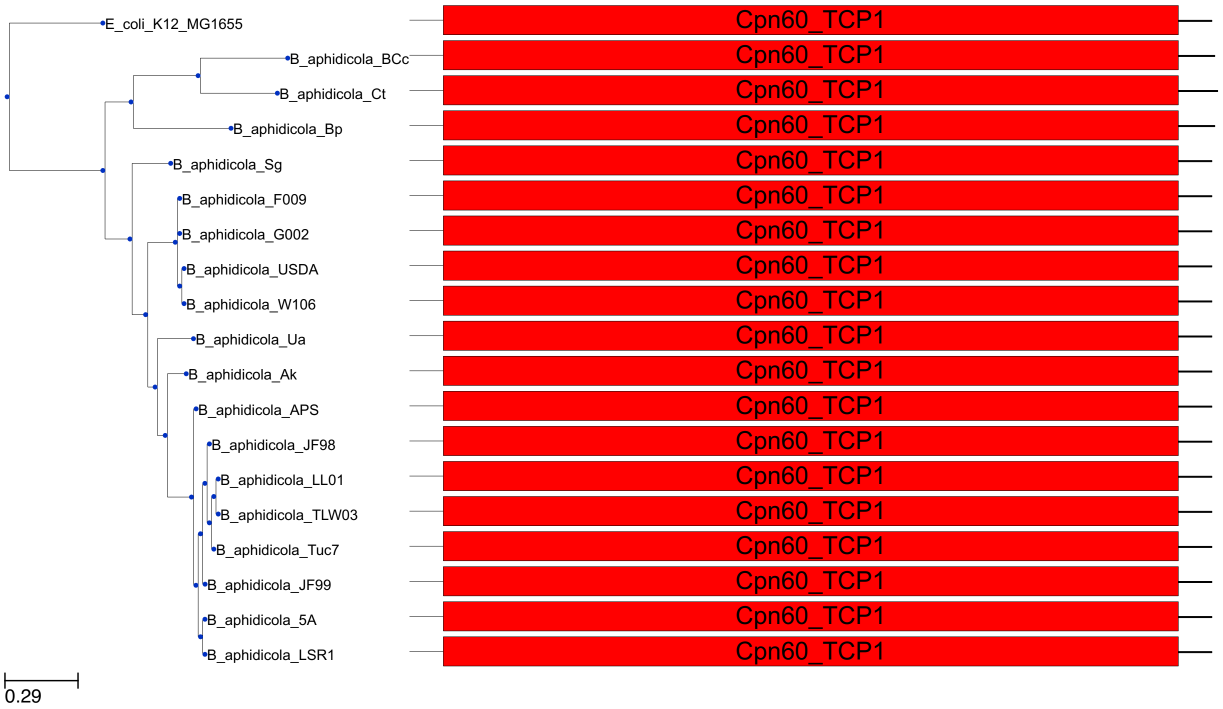

groL
NP_418567.1
"Cpn60 chaperonin GroEL, large subunit of GroESL"

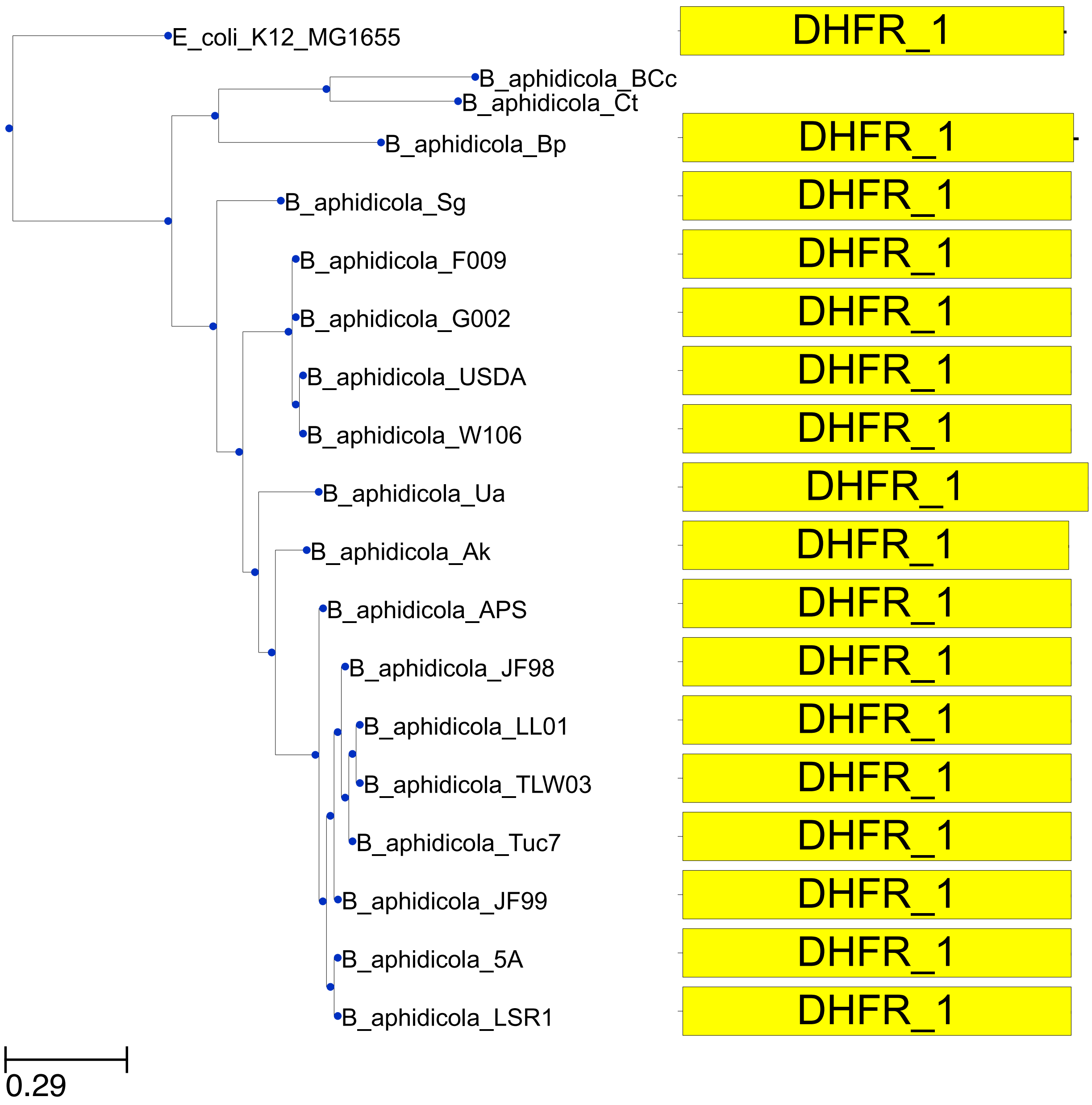

folA
NP_414590.1
dihydrofolate reductase

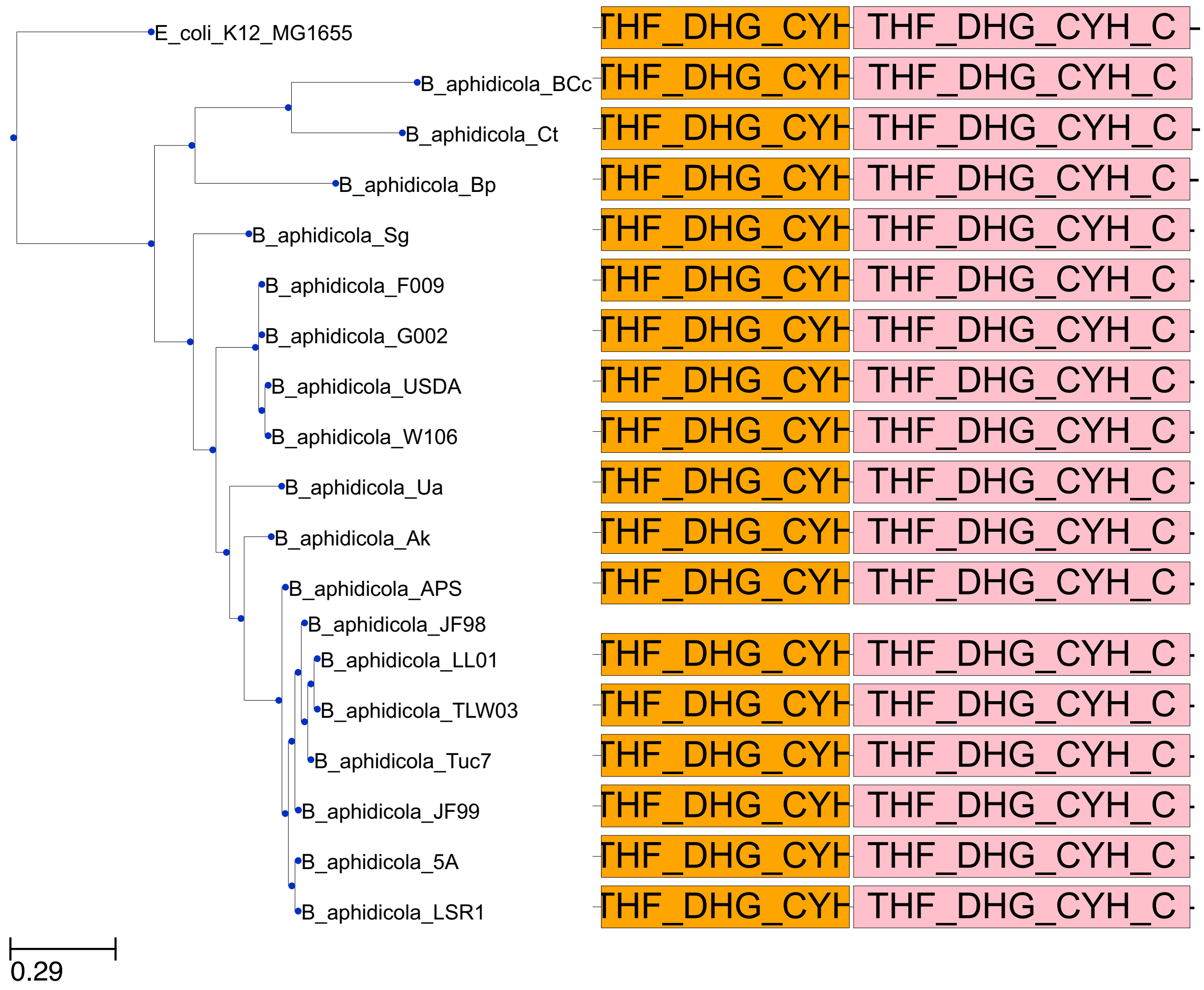

folD

NP_415062.1

"bifunctional 5,10-methylene-tetrahydrofolate dehydrogenase/ 5,10-methylene-tetrahydrofolate cyclohydrolase"

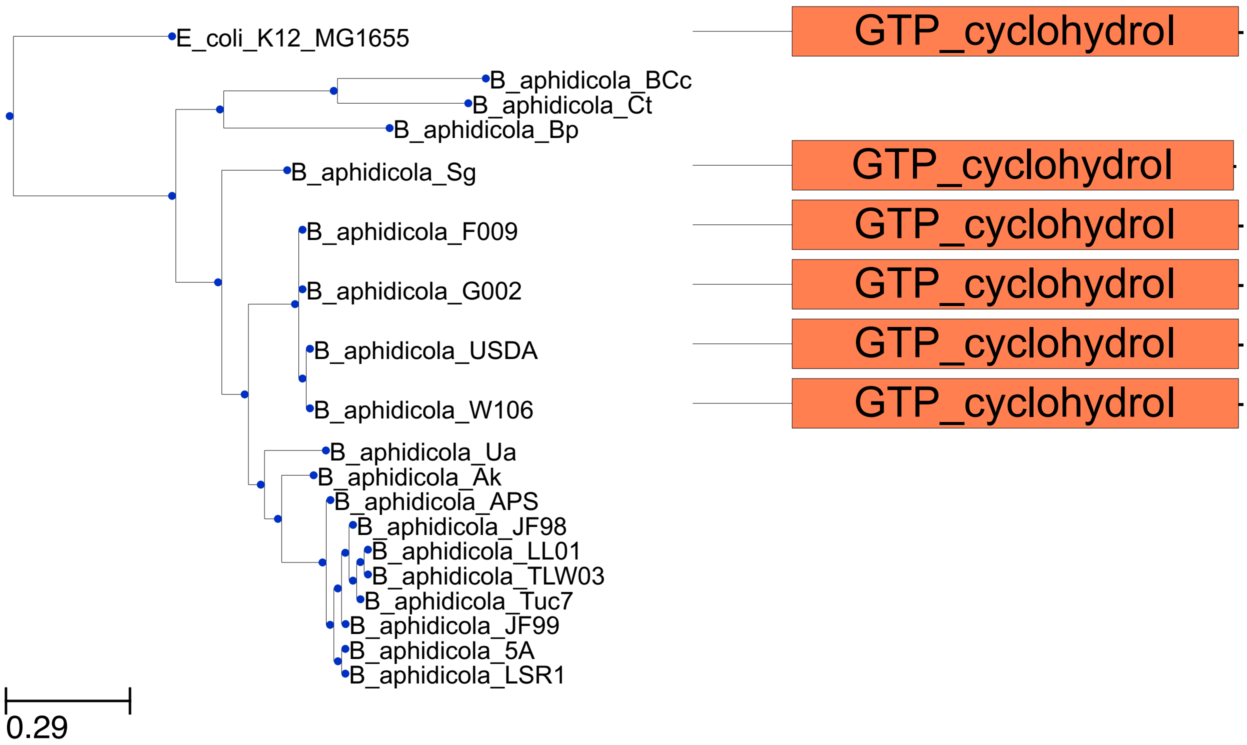

folE
NP_416658.1
GTP cyclohydrolase I

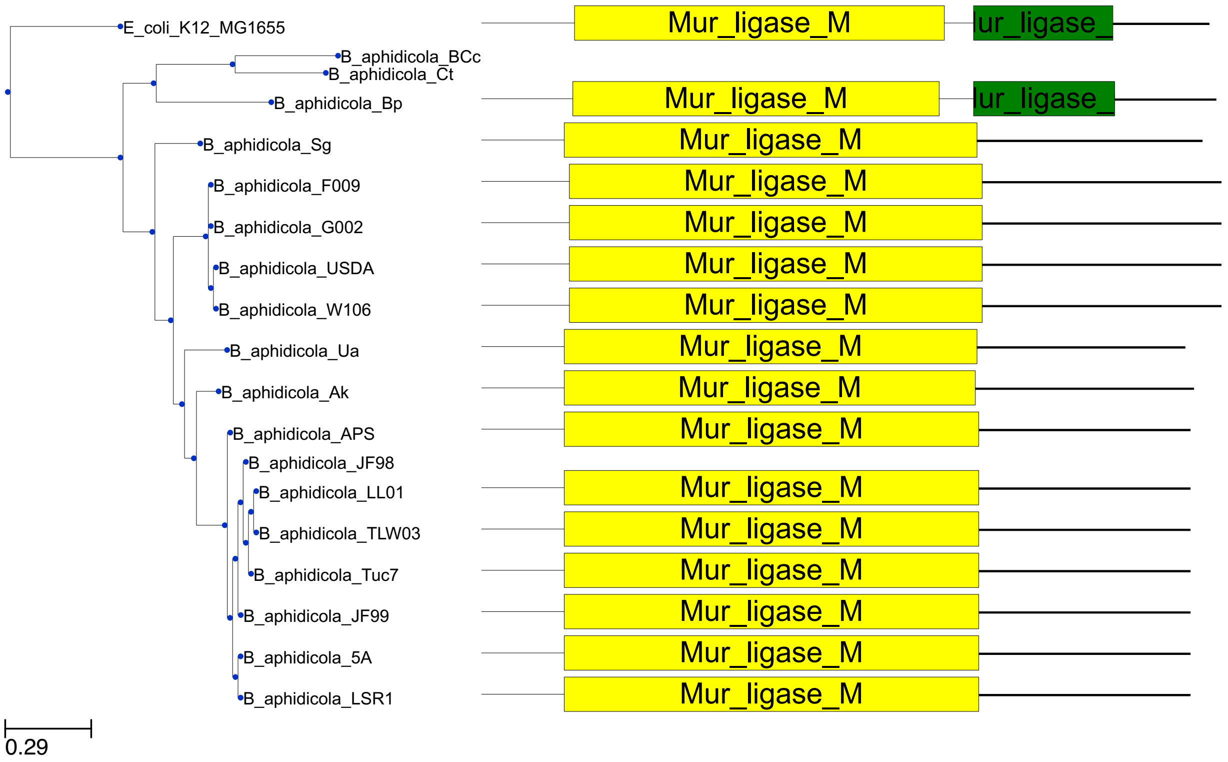

folC
NP_416818.1
bifunctional folylpolyglutamate synthase/ dihydrofolate synthase

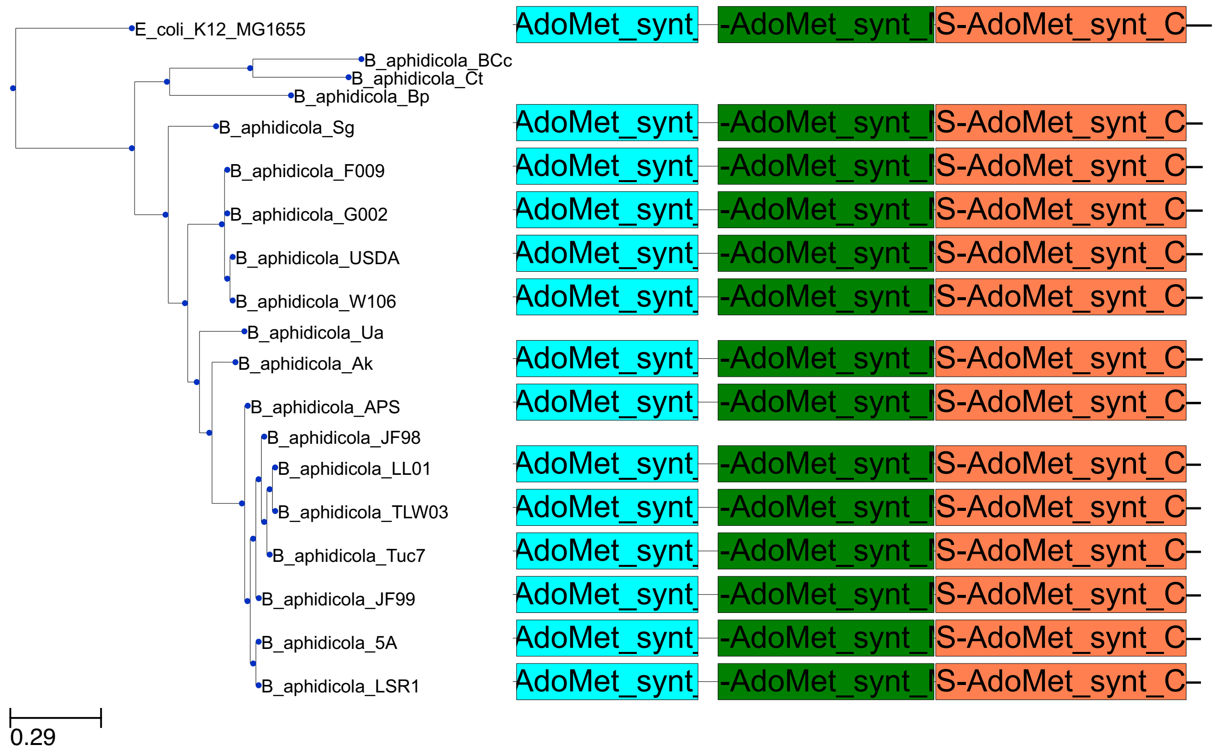

metK

NP_417417.1

S-adenosylmethionine synthetase

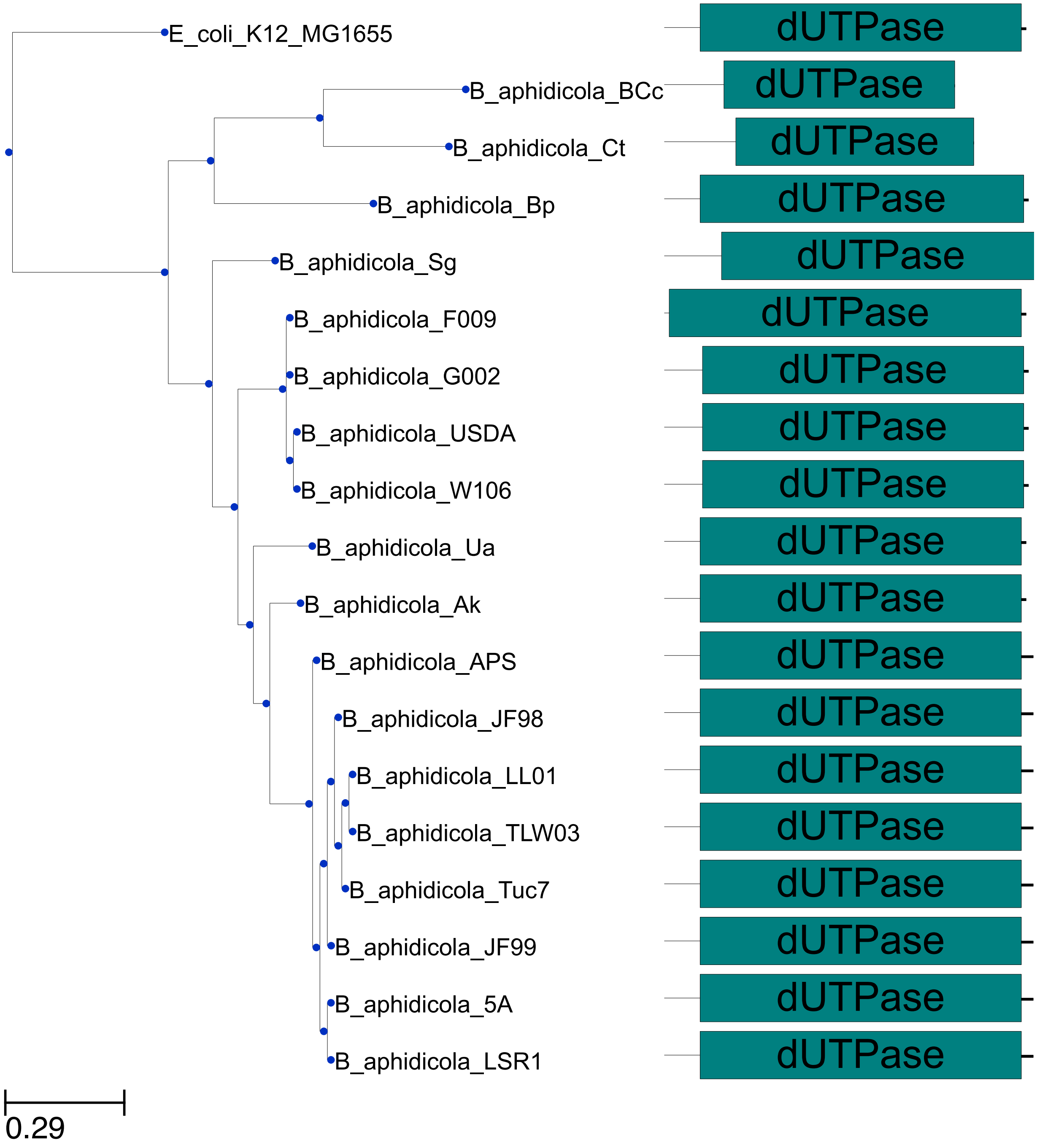

dut
NP_418097.1
deoxyuridinetriphosphatase

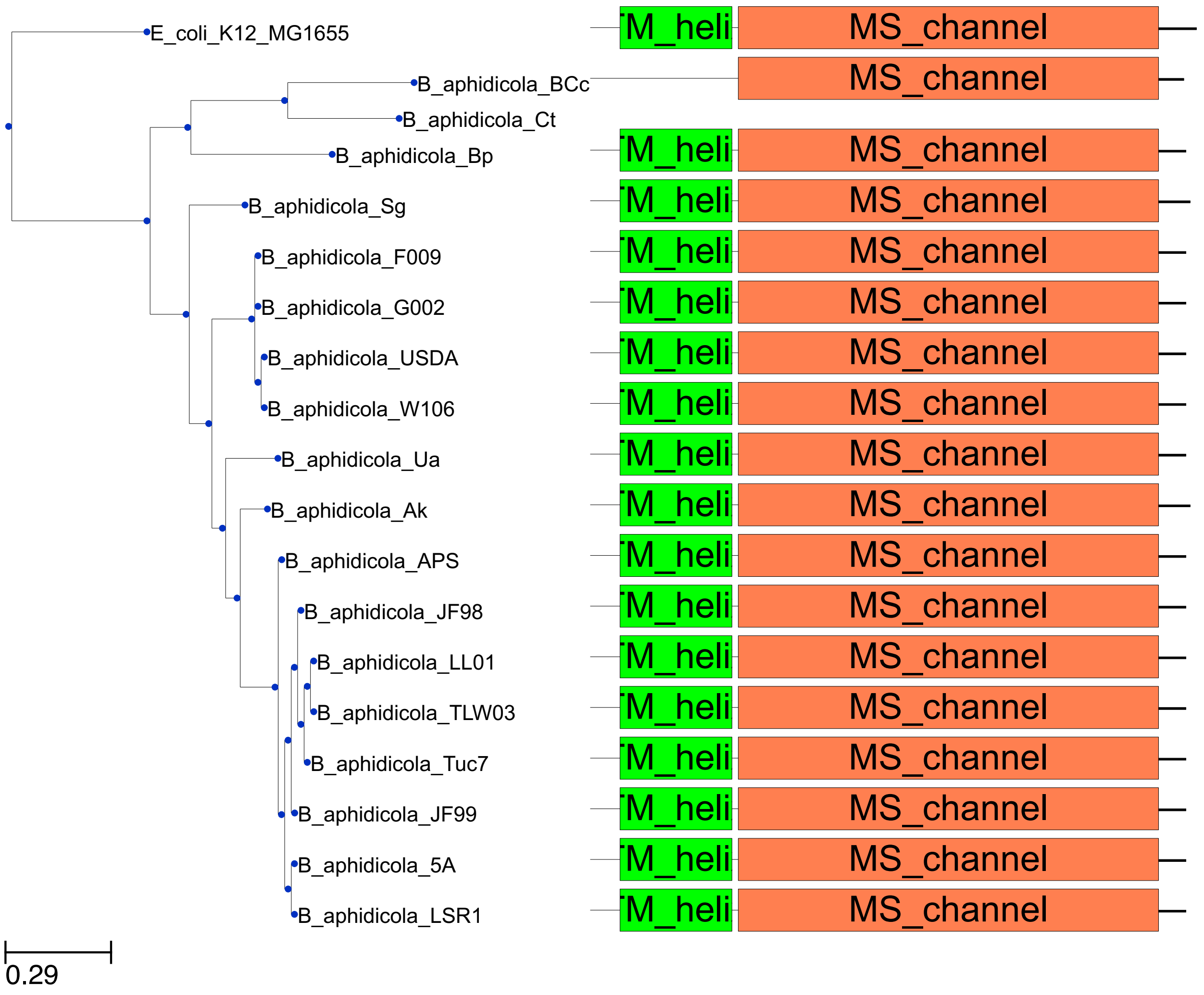

mscS

NP_417399.1

"mechanosensitive channel protein, small conductance"

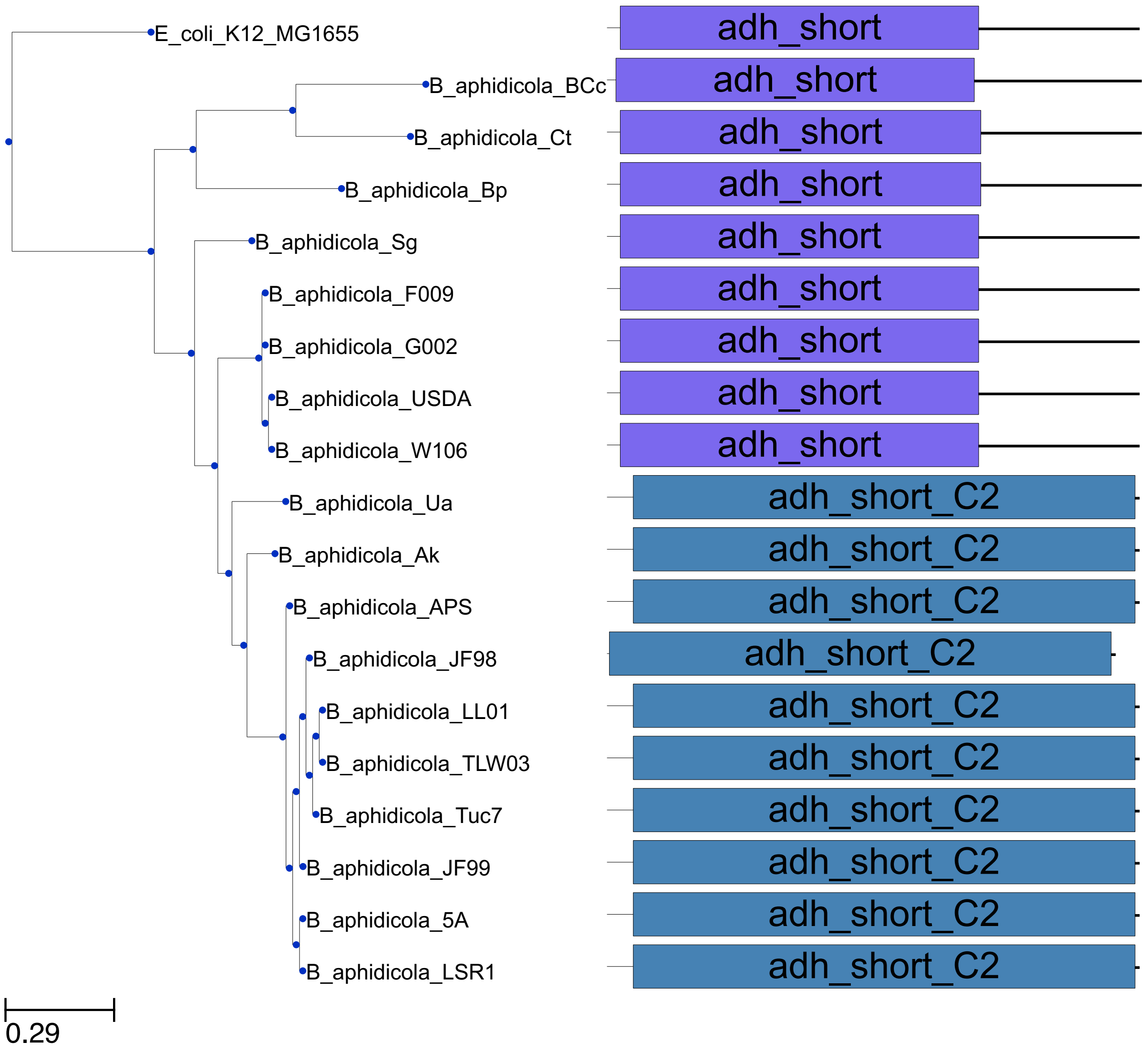

fabG
NP_415611.1
3-oxoacyl-[acyl-carrier-protein] reductase

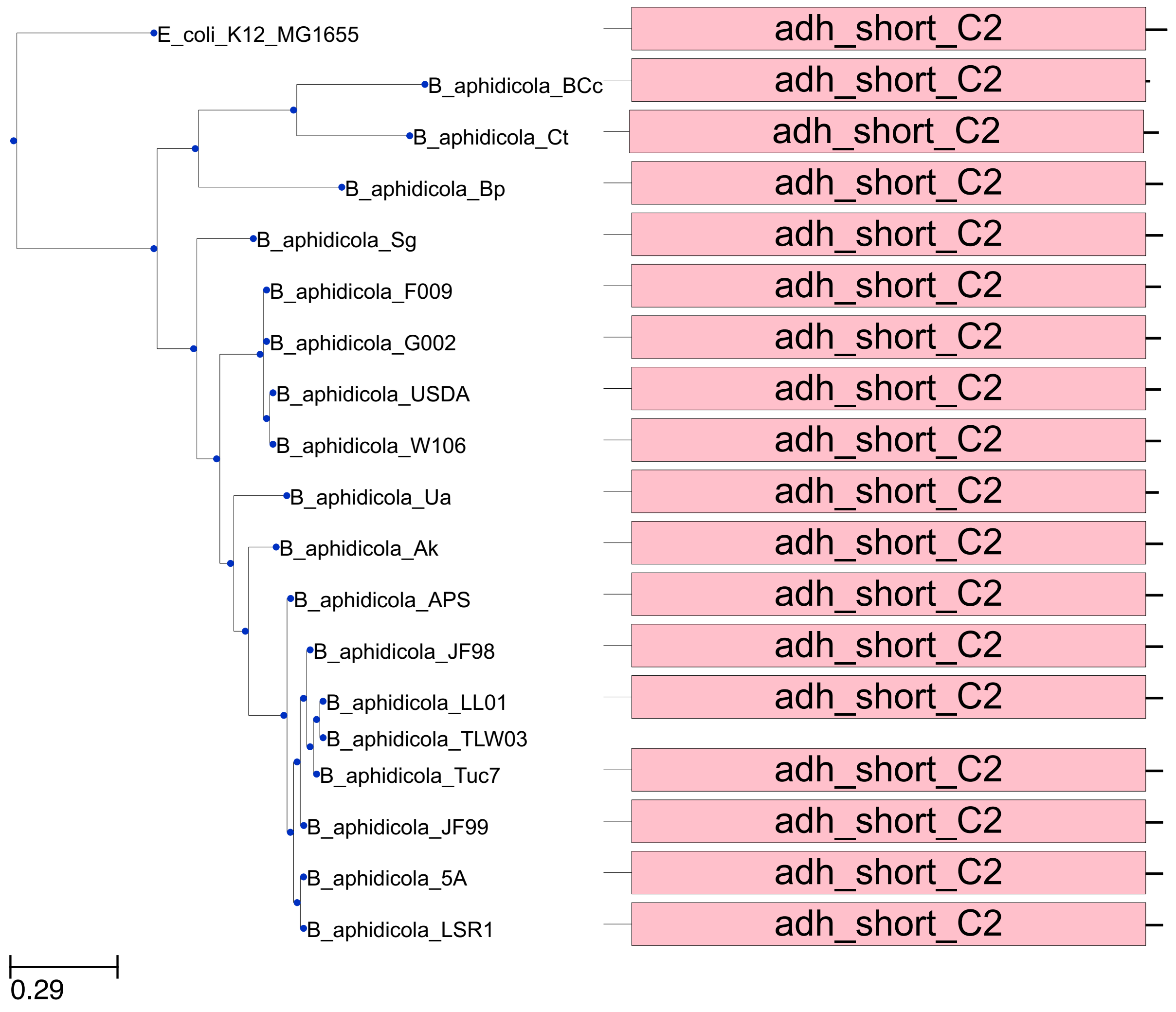

fabI
NP_415804.1
"enoyl-[acyl-carrier-protein] reductase, NADH-dependent"

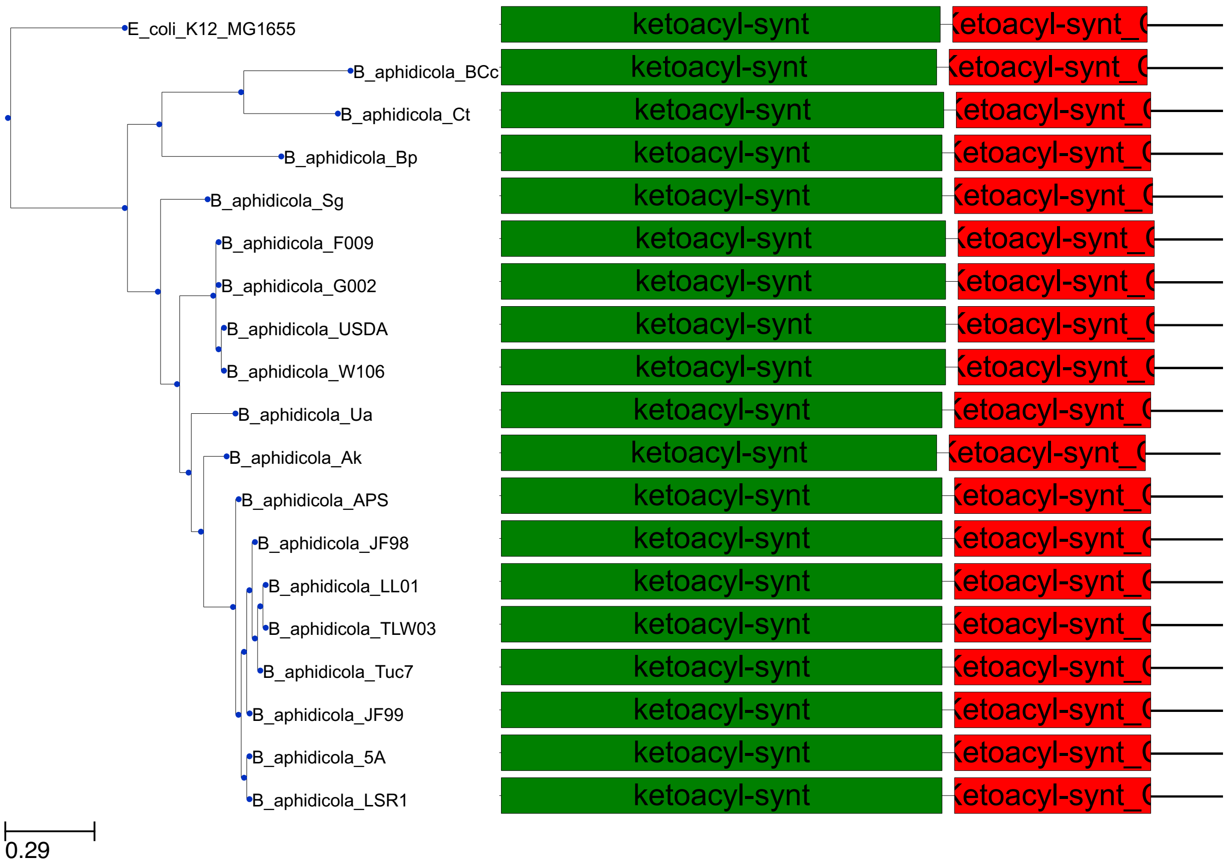

fabB
NP_416826.1
3-oxoacyl-[acyl-carrier-protein] synthase I

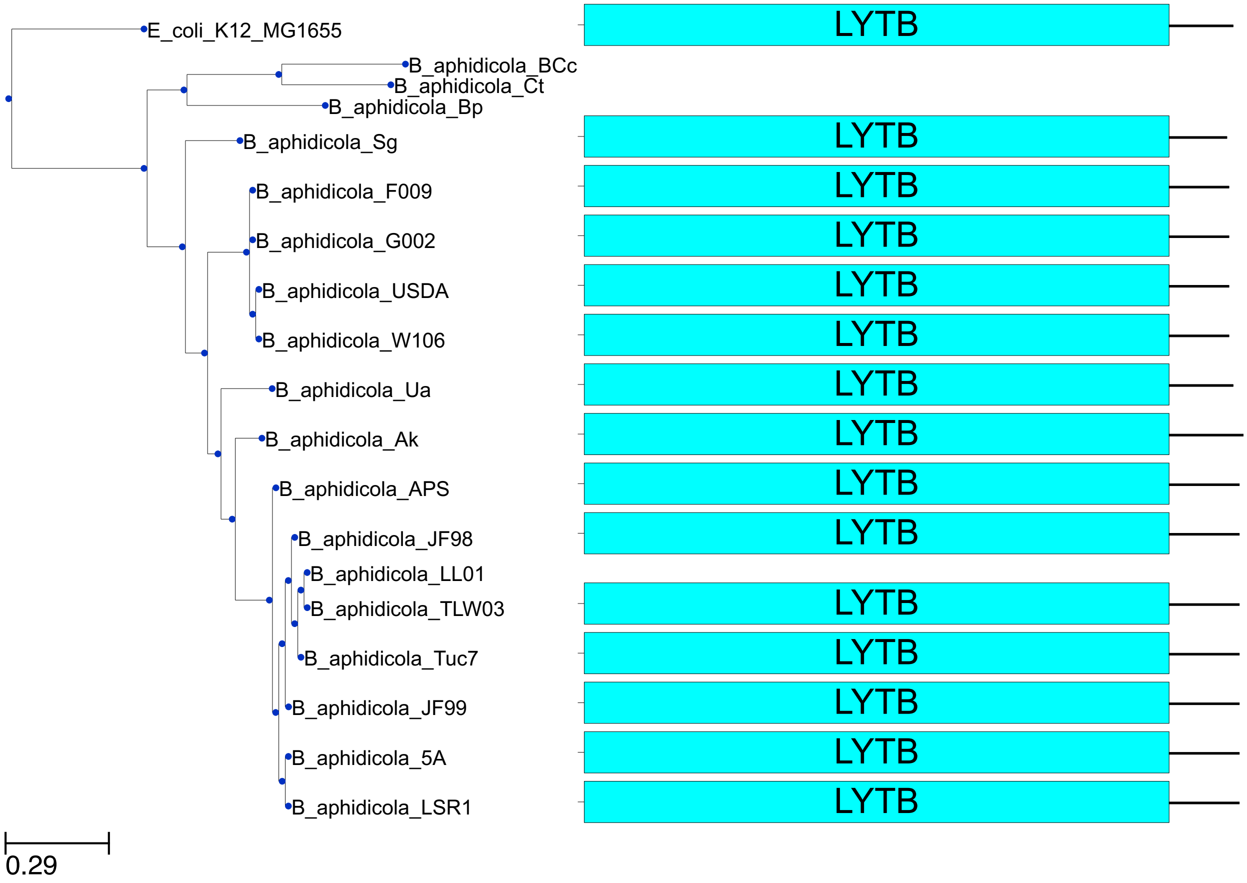

ispH
NP_414570.1
"4-hydroxy-3-methylbut-2-enyl diphosphate reductase, 4Fe-4S protein"

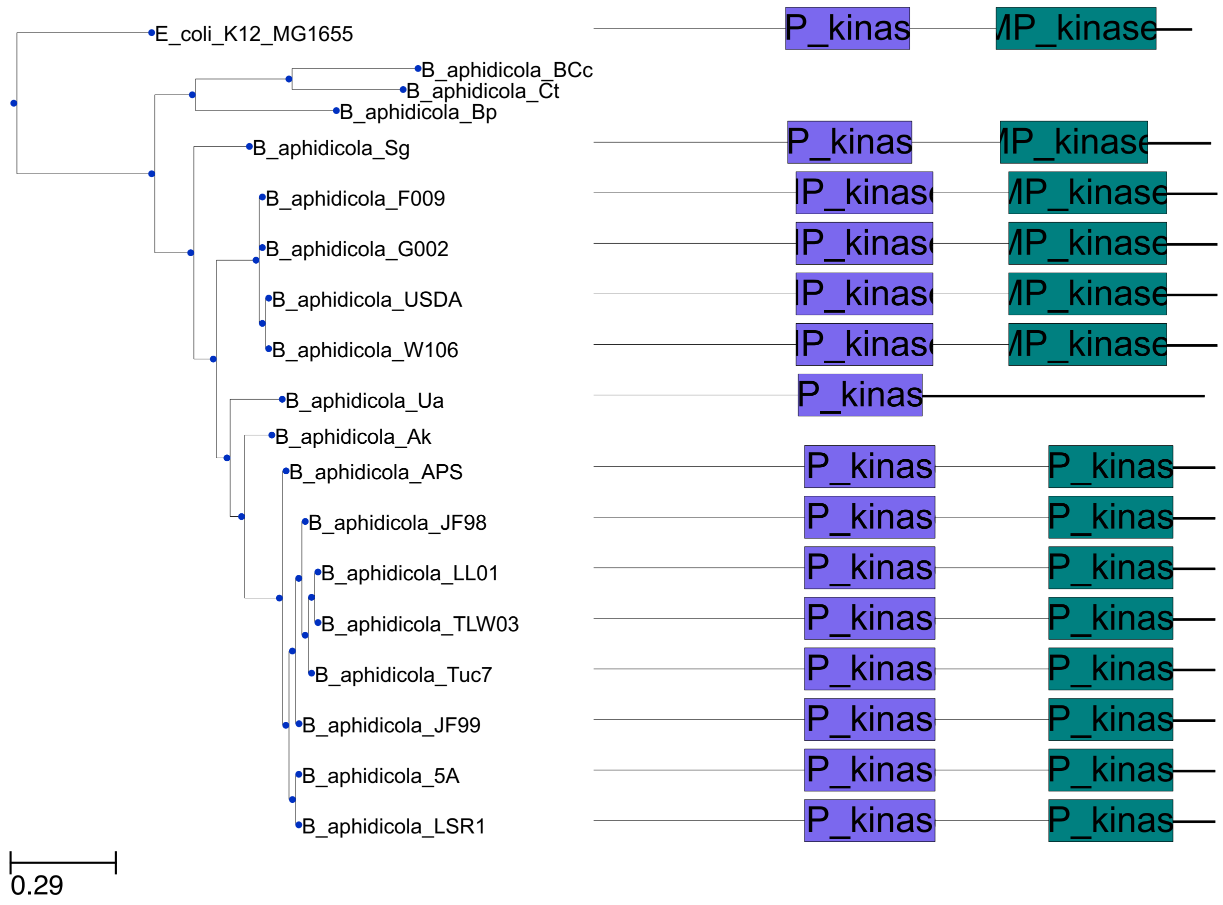

ispE

NP_415726.1

4-diphosphocytidyl-2-C-methylerythritol kinase

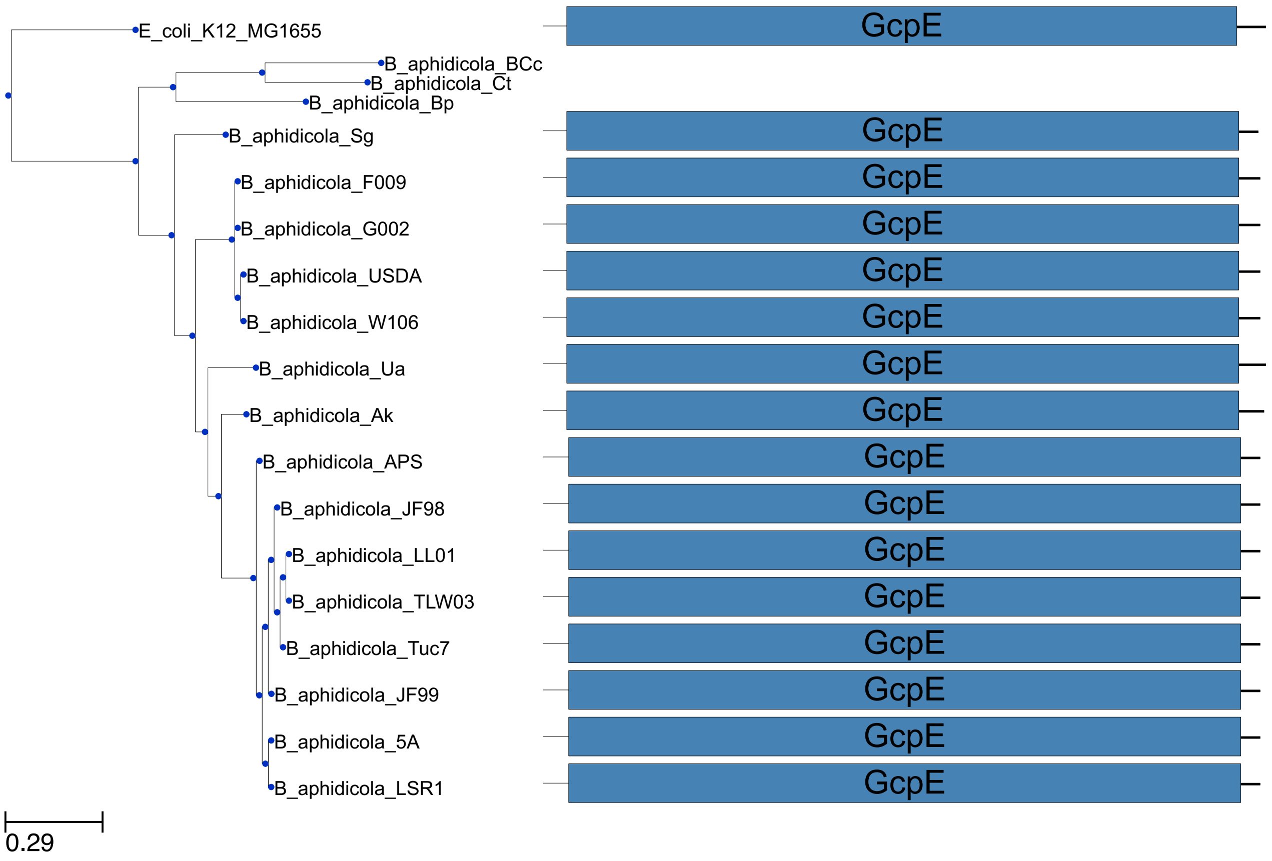

ispG

NP_417010.1

1-hydroxy-2-methyl-2-(E)-butenyl 4-diphosphate synthase

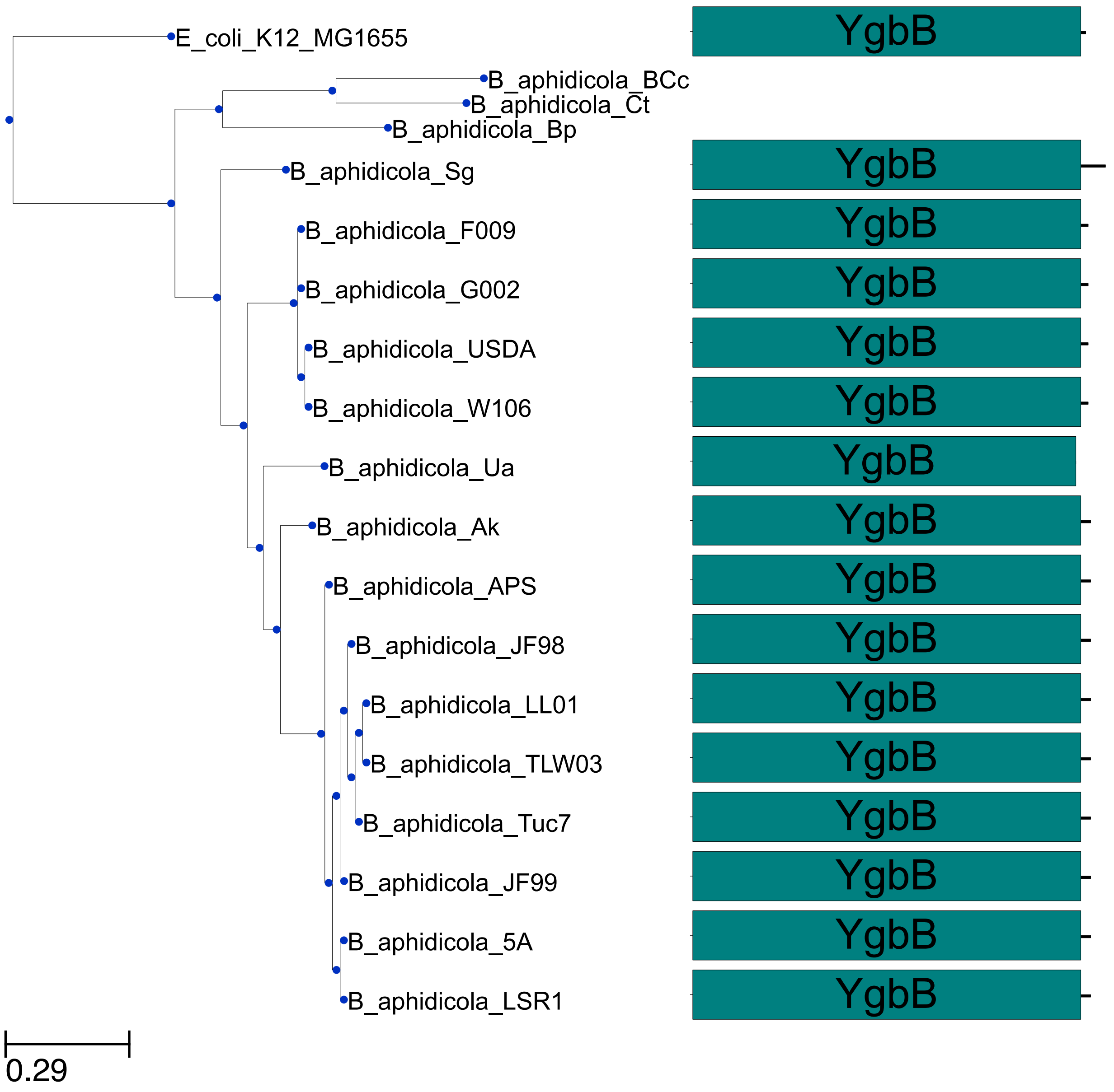

ispF
NP_417226.1
"2C-methyl-D-erythritol 2,4-cyclodiphosphate synthase"

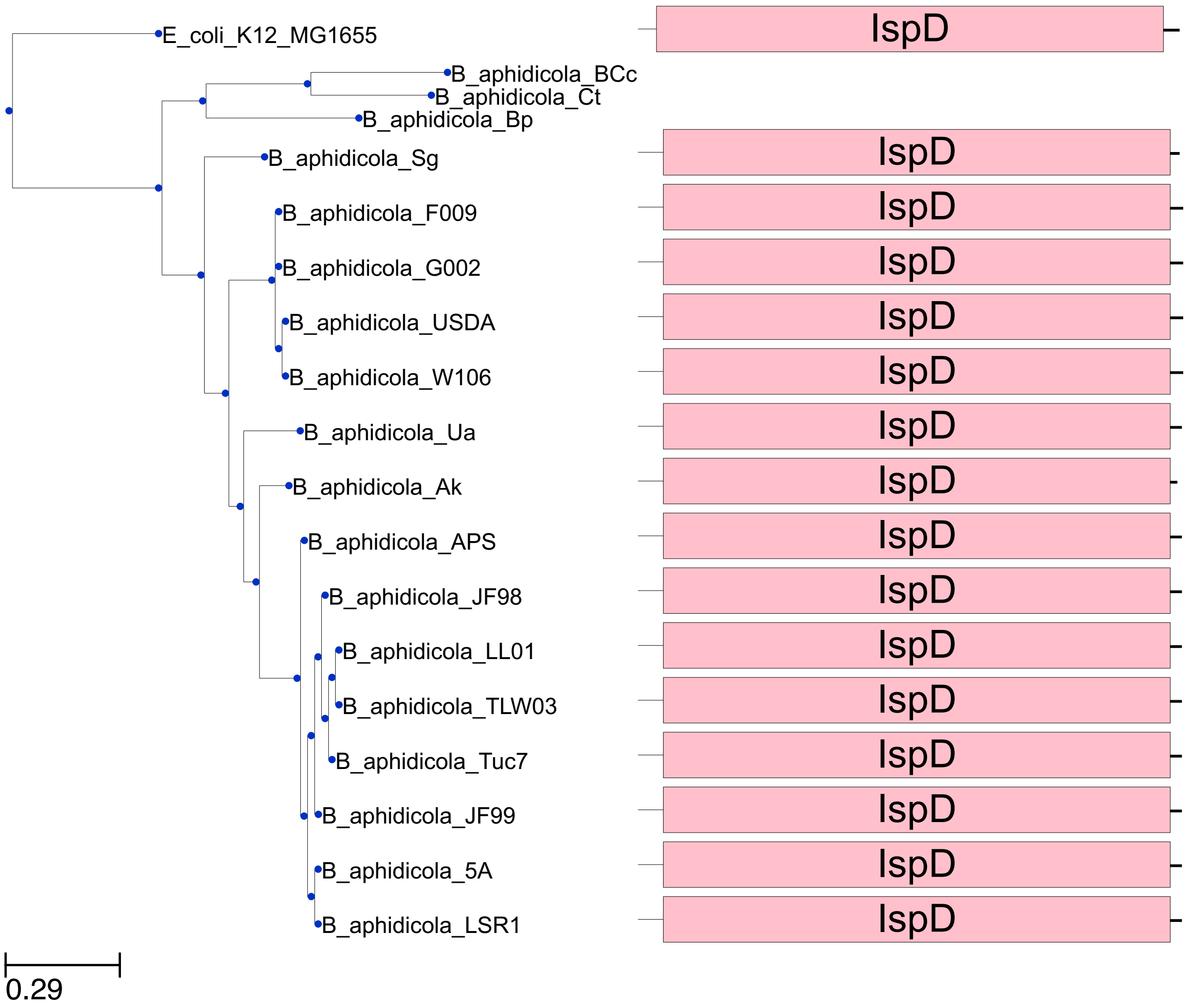

ispD
NP_417227.1
4-diphosphocytidyl-2C-methyl-D-erythritol synthase

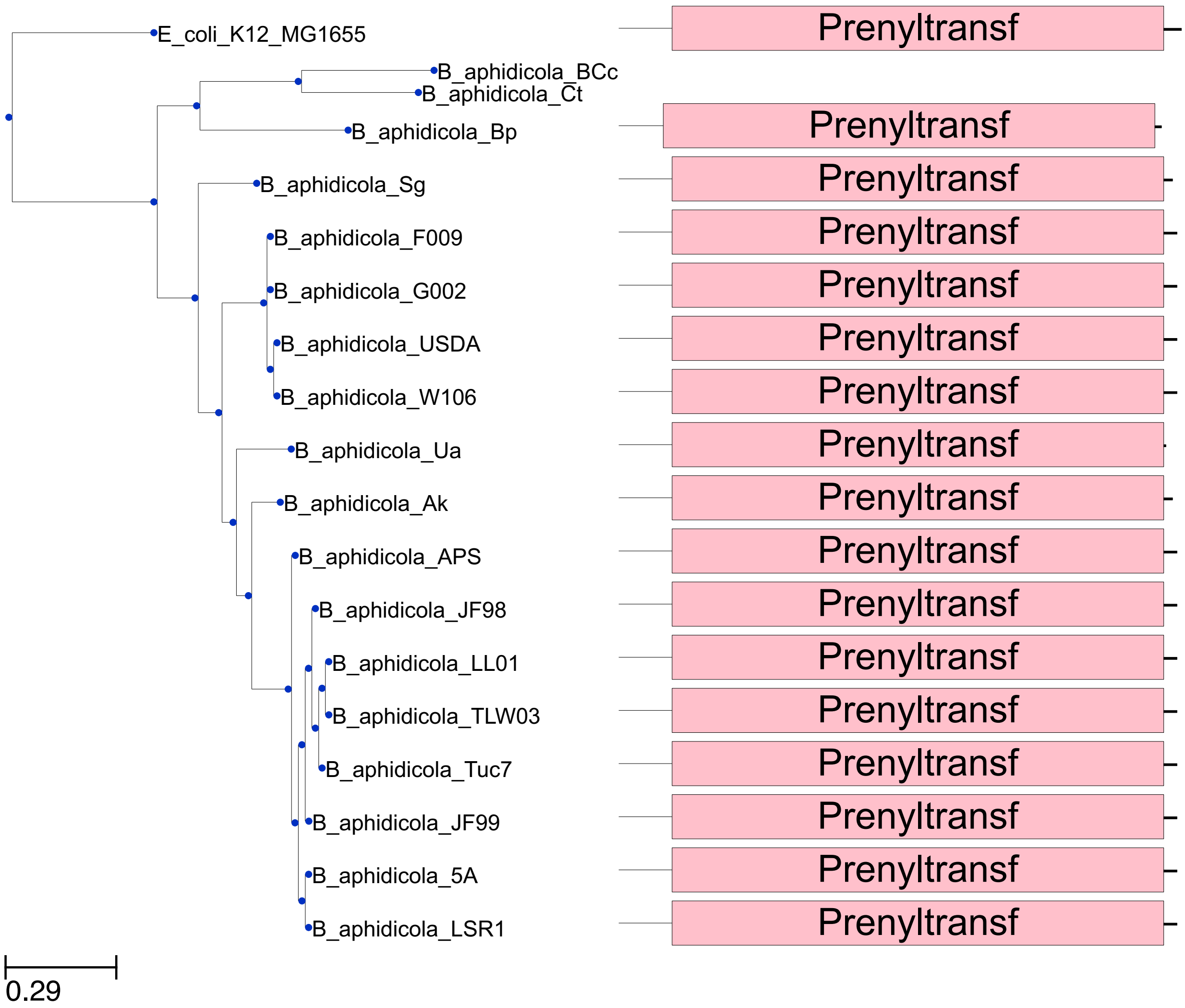

ispU
NP_414716.1
undecaprenyl pyrophosphate synthase

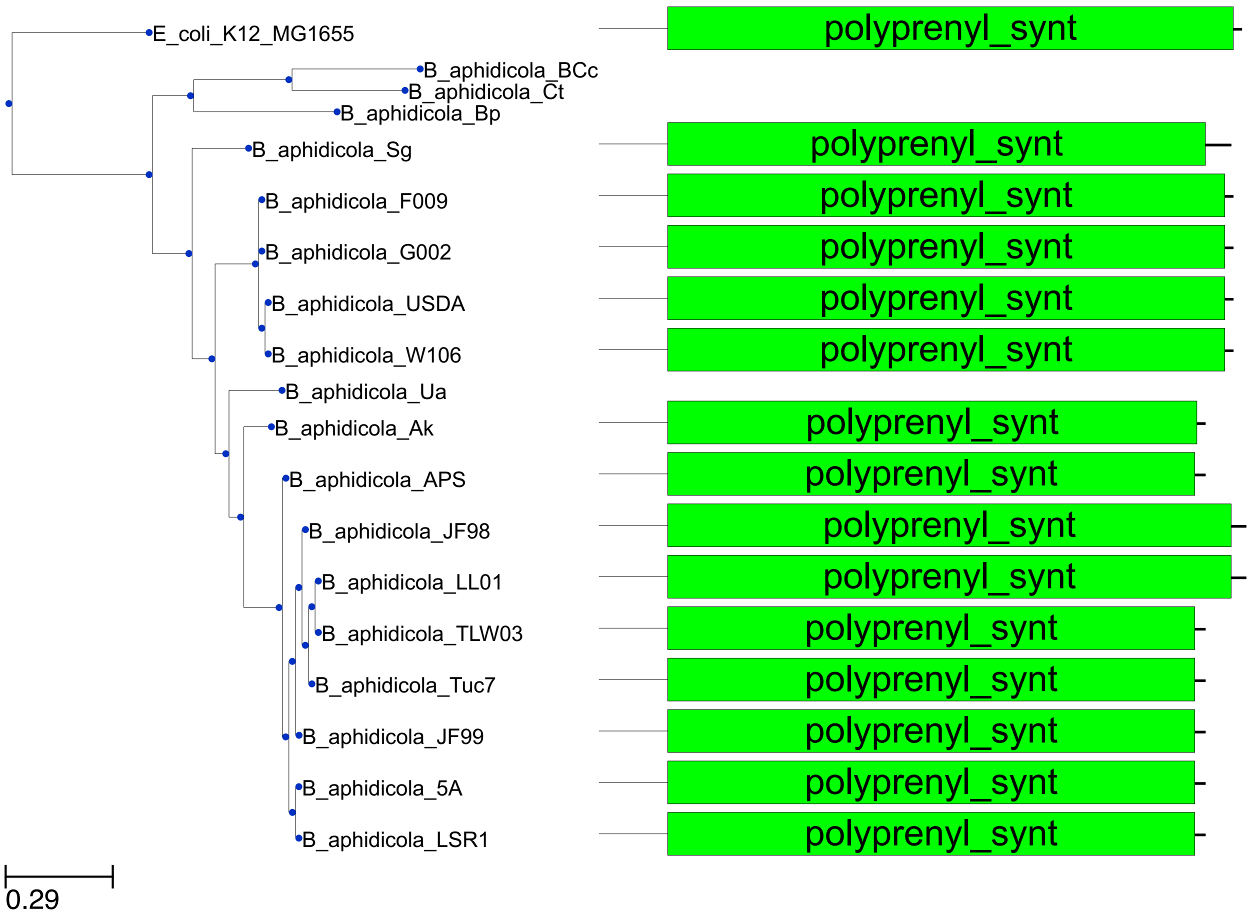

ispA
NP_414955.1
geranyltranstransferase

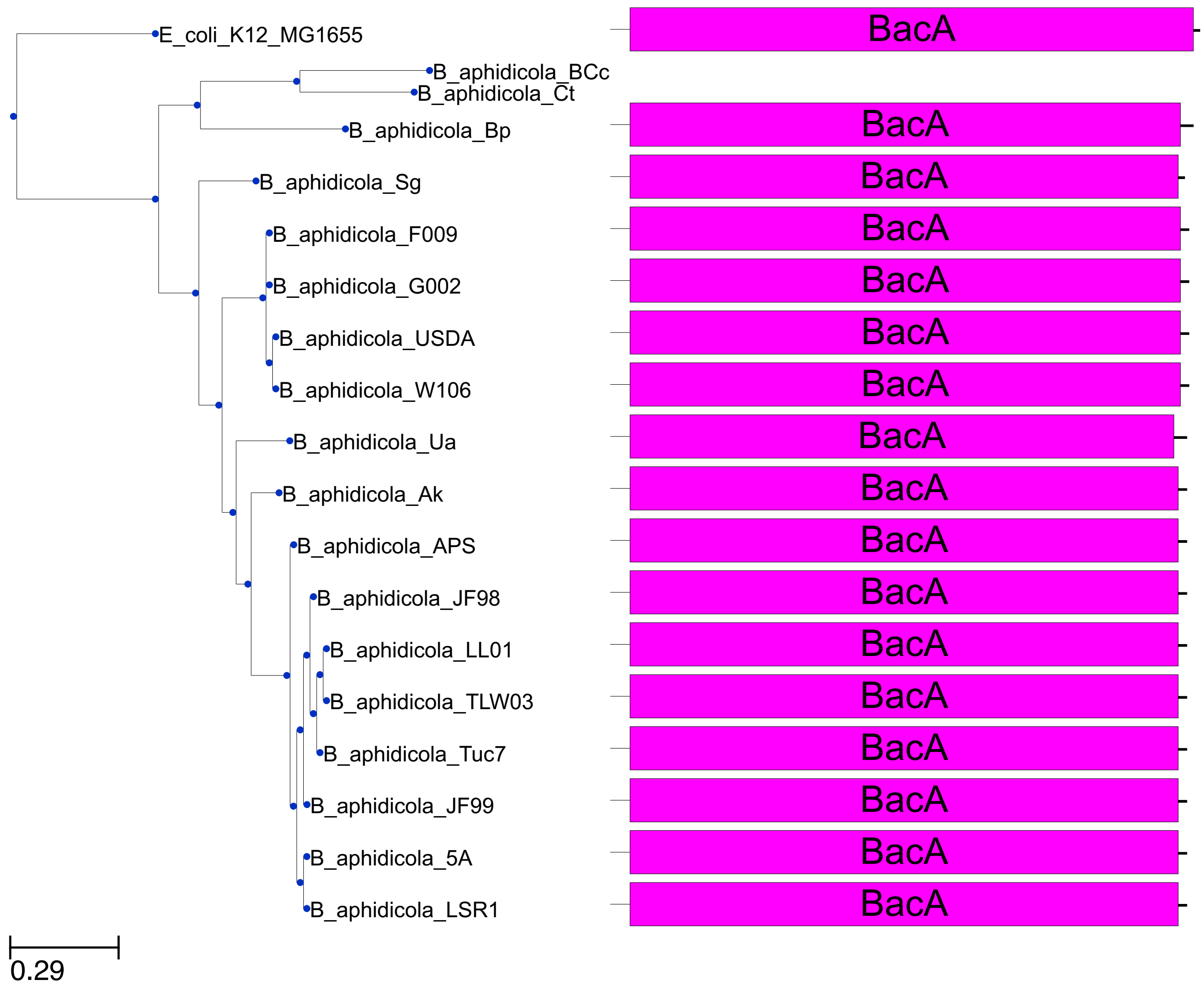

bacA
NP_417529.1
undecaprenyl pyrophosphate phosphatase

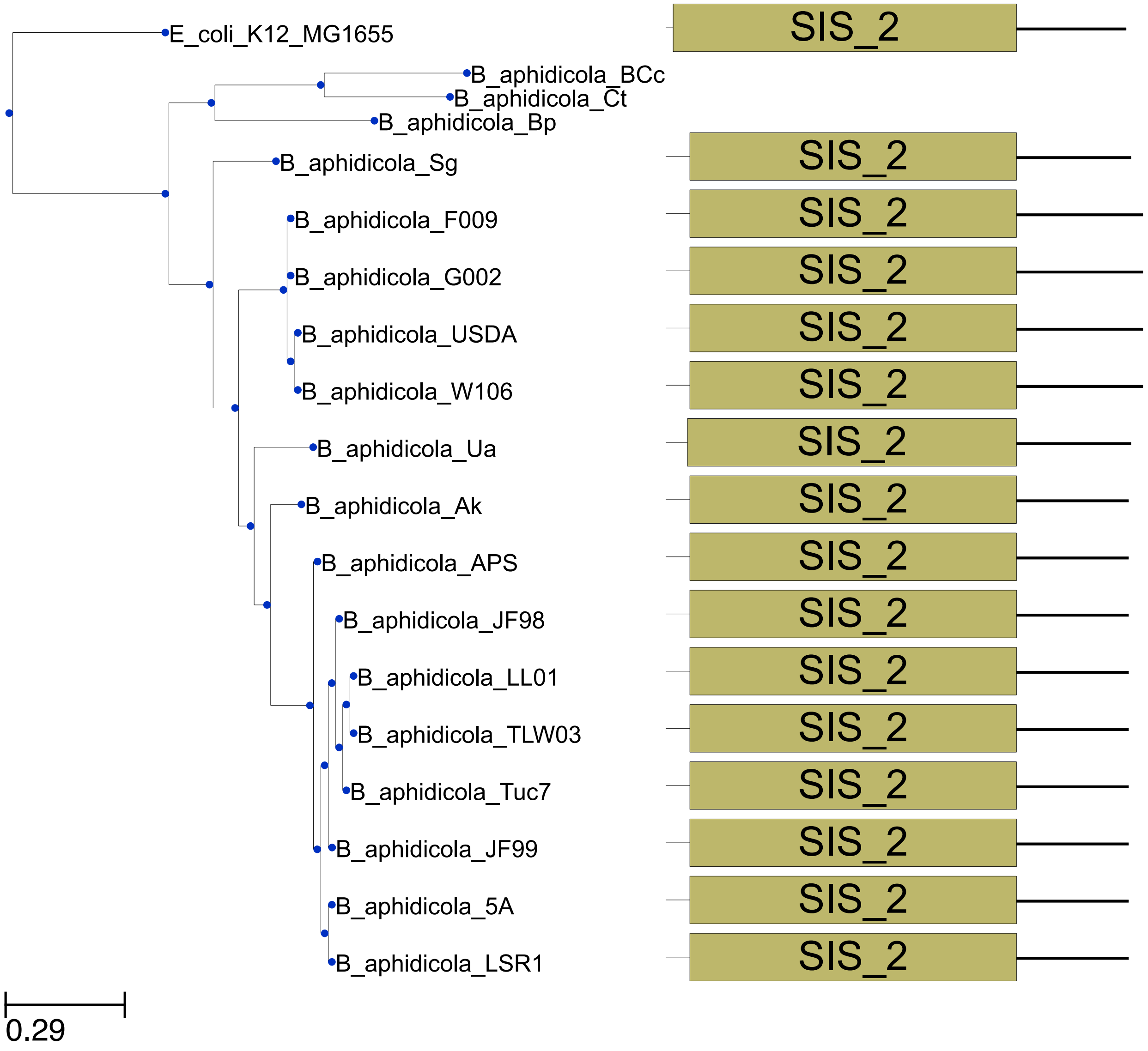

lpcA
NP_414757.1
D-sedoheptulose 7-phosphate isomerase

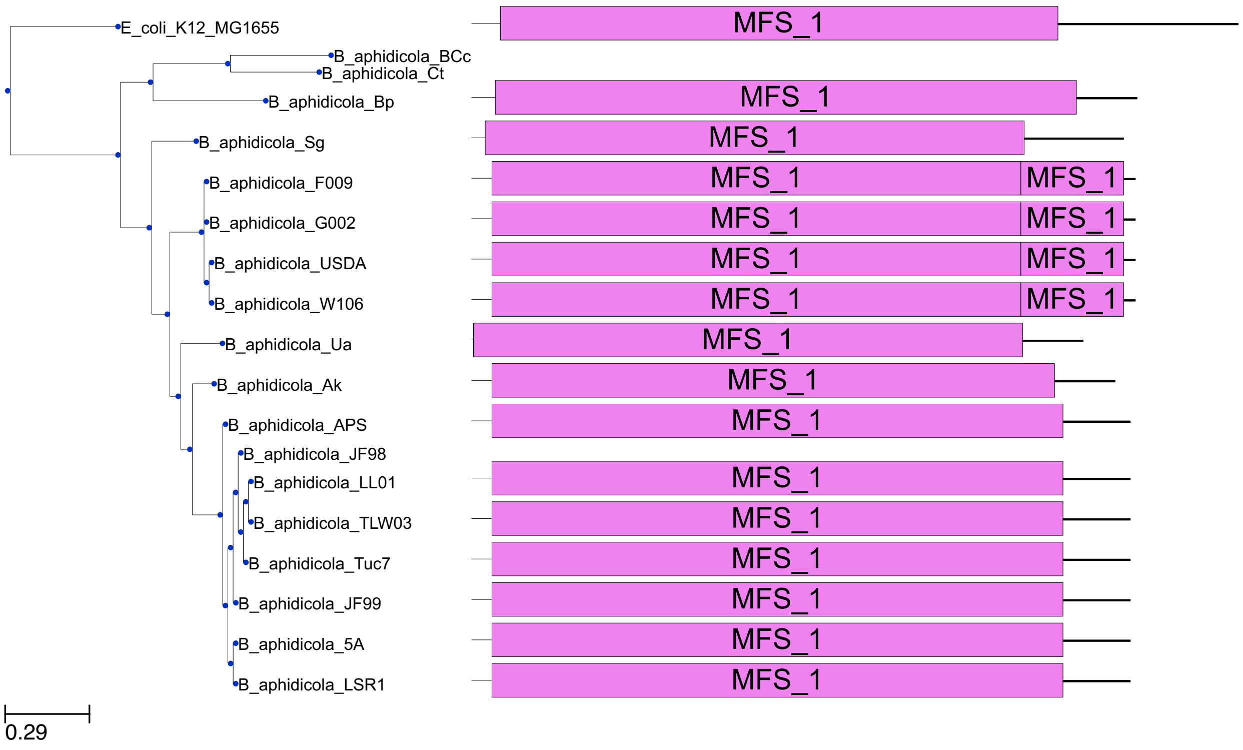

yajR
NP_414961.4
putative transporter

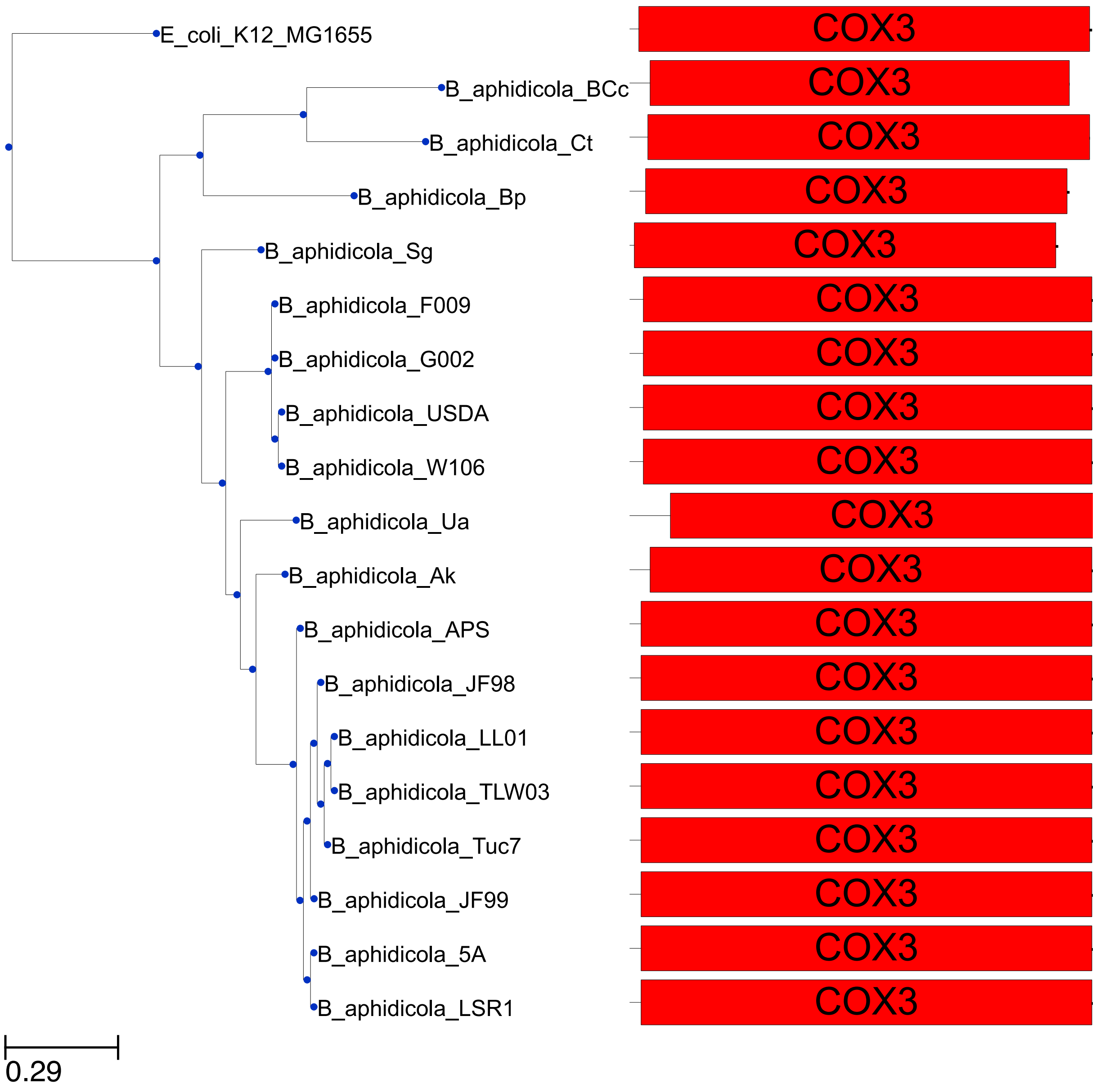

cyoC
NP_414964.1
cytochrome o ubiquinol oxidase subunit III

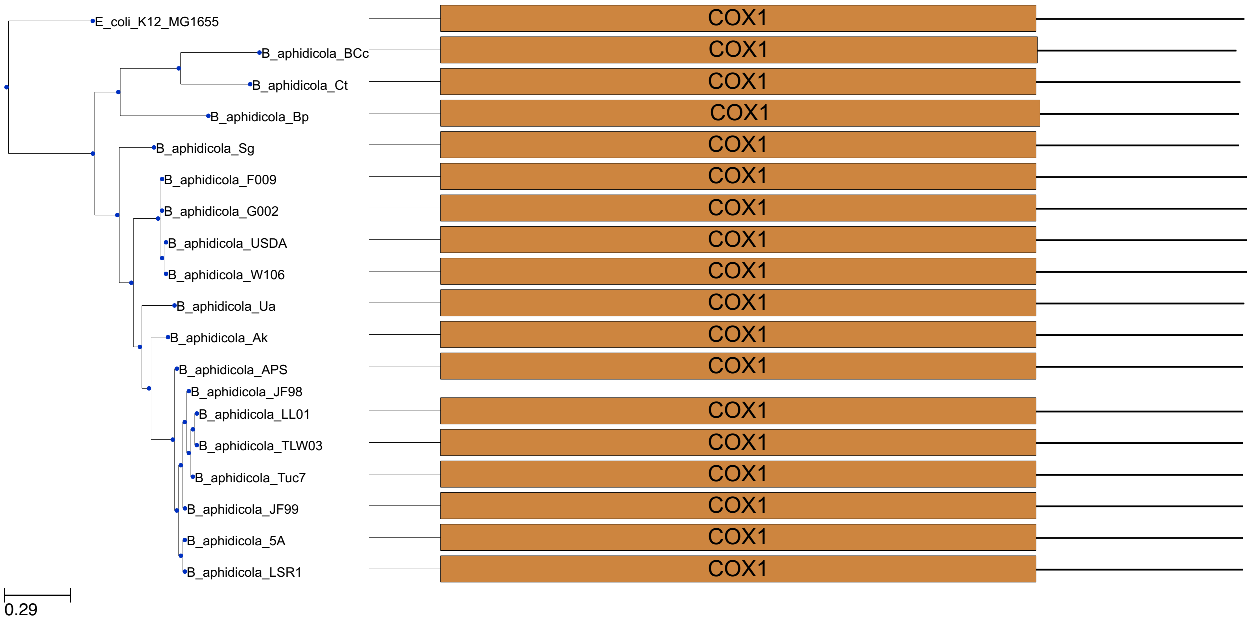

cyoB
NP_414965.1
cytochrome o ubiquinol oxidase subunit I

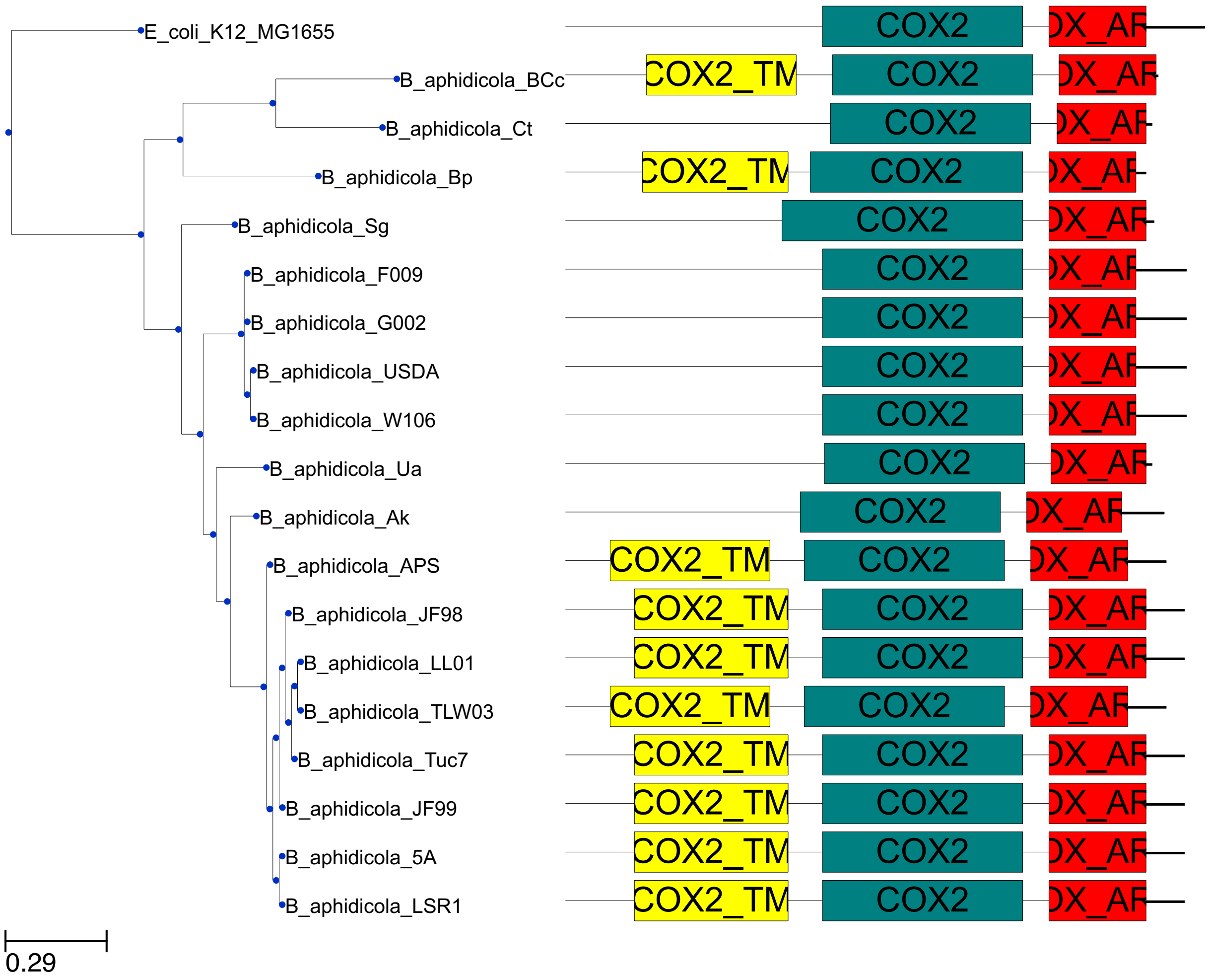

cyoA
NP_414966.1
cytochrome o ubiquinol oxidase subunit II

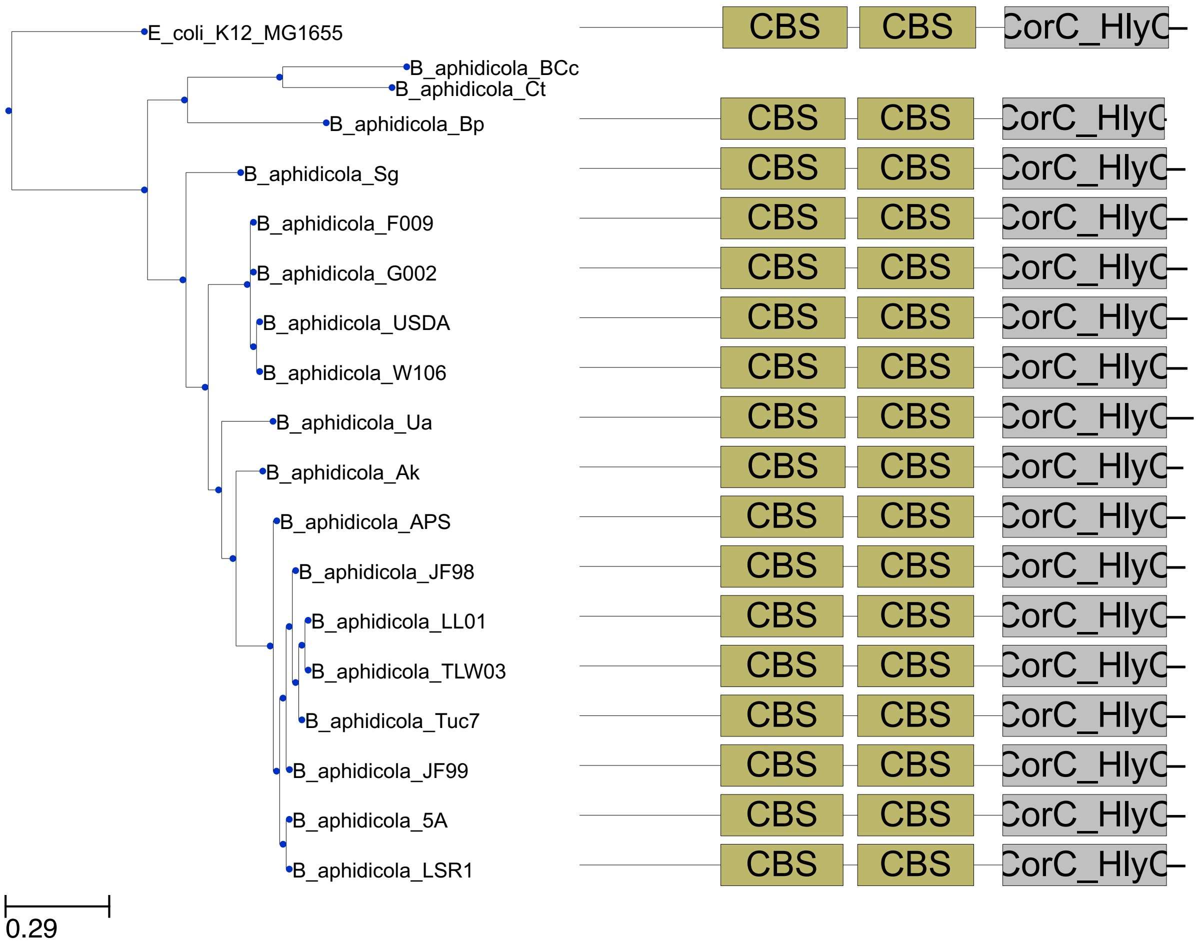

ybeX
NP_415191.1
putative ion transport

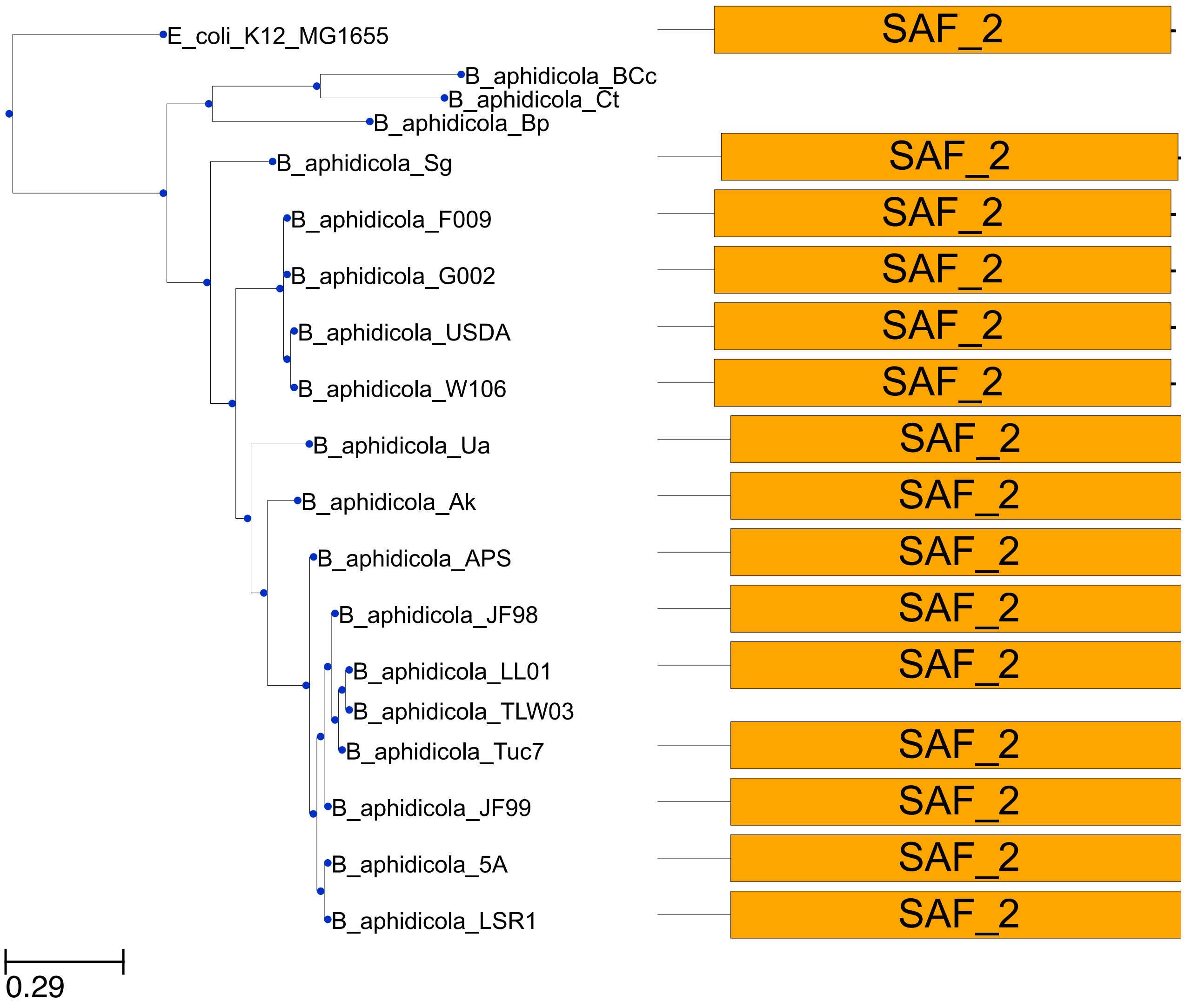

flgA
NP_415590.1
assembly protein for flagellar basal-body periplasmic P ring

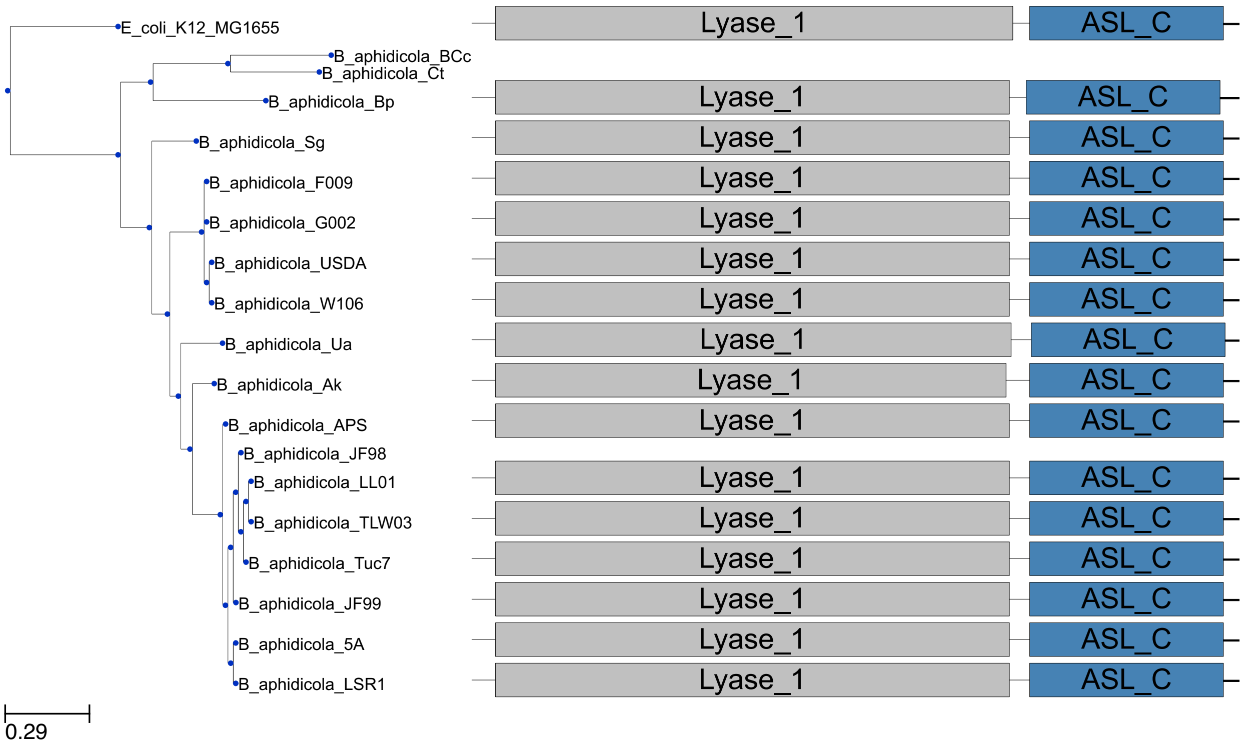

purB
NP_415649.1
adenylosuccinate lyase

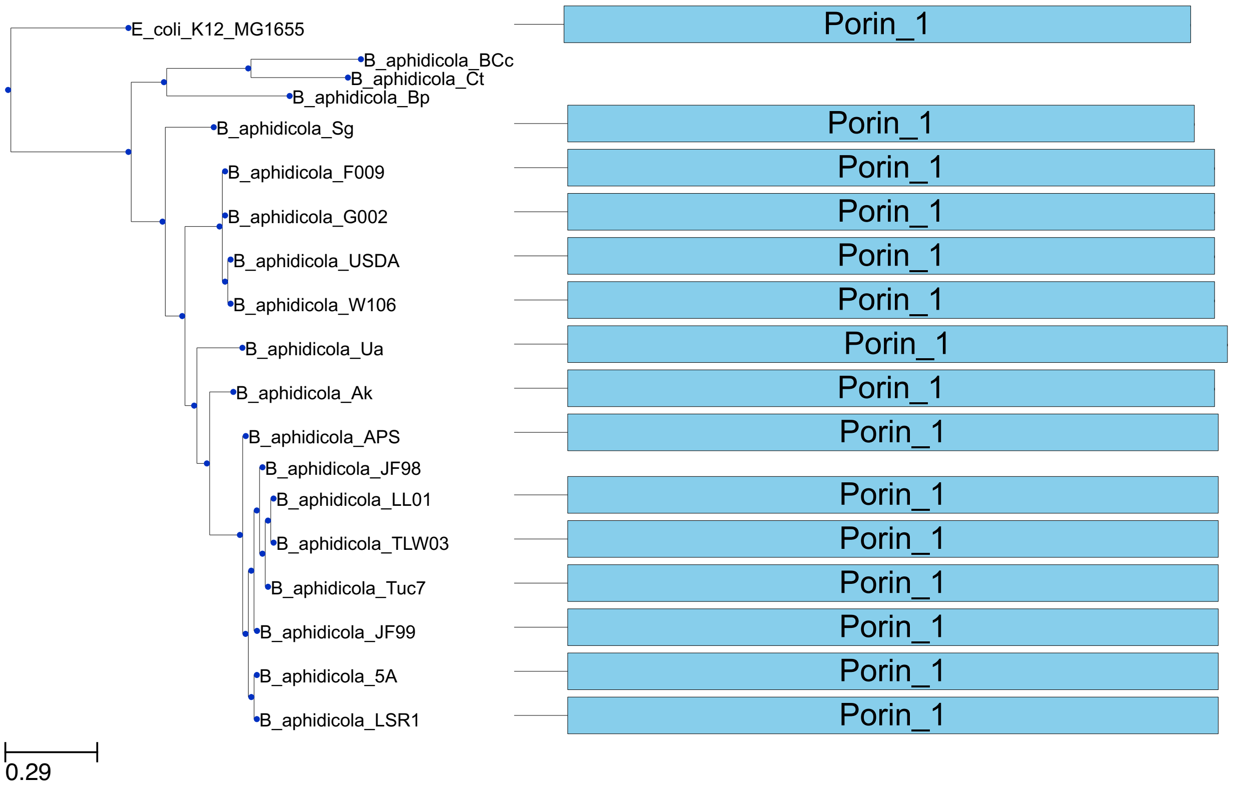

ompN

NP_415895.1

"outer membrane pore protein N, non-specific"

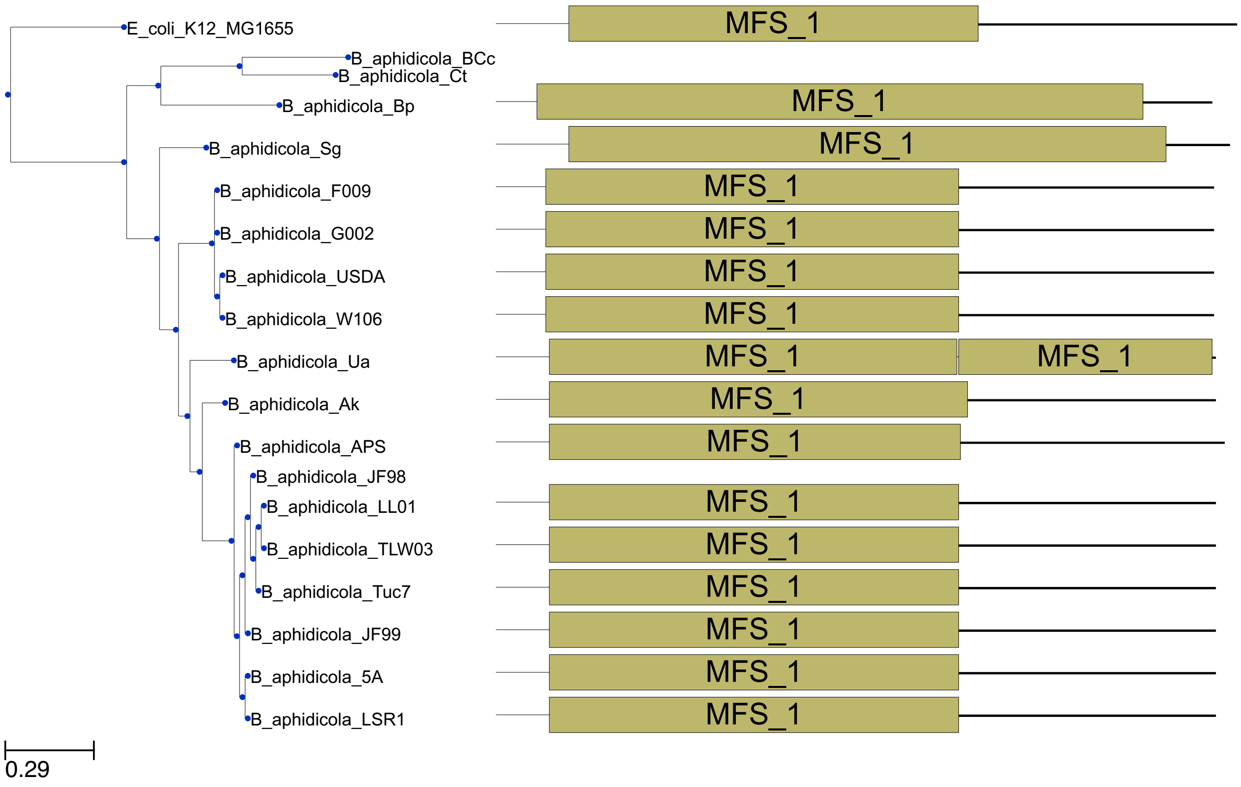

ynfM
NP_416113.1
putative arabinose efflux transporter

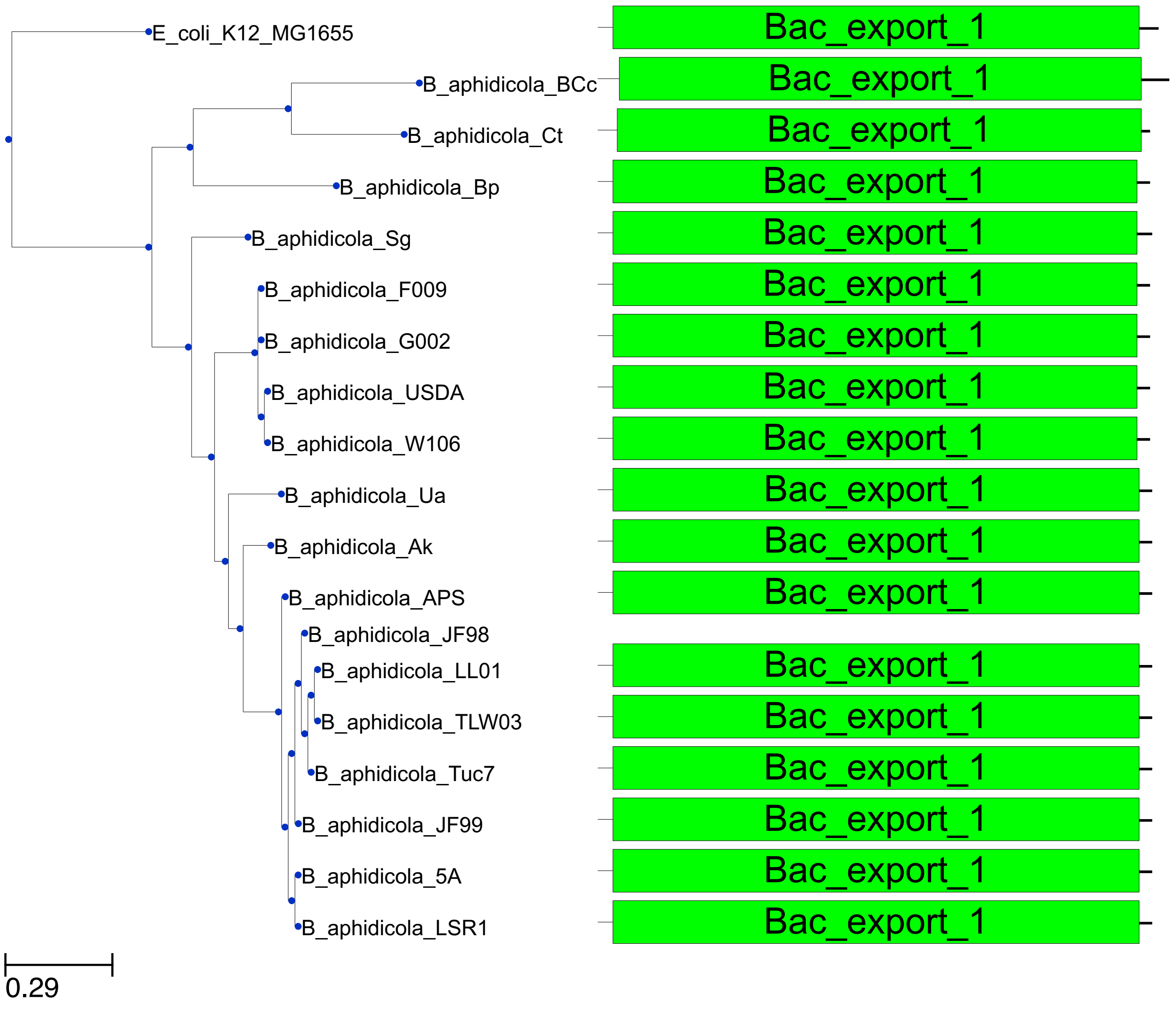

fliR
NP_416460.1
flagellar export pore protein

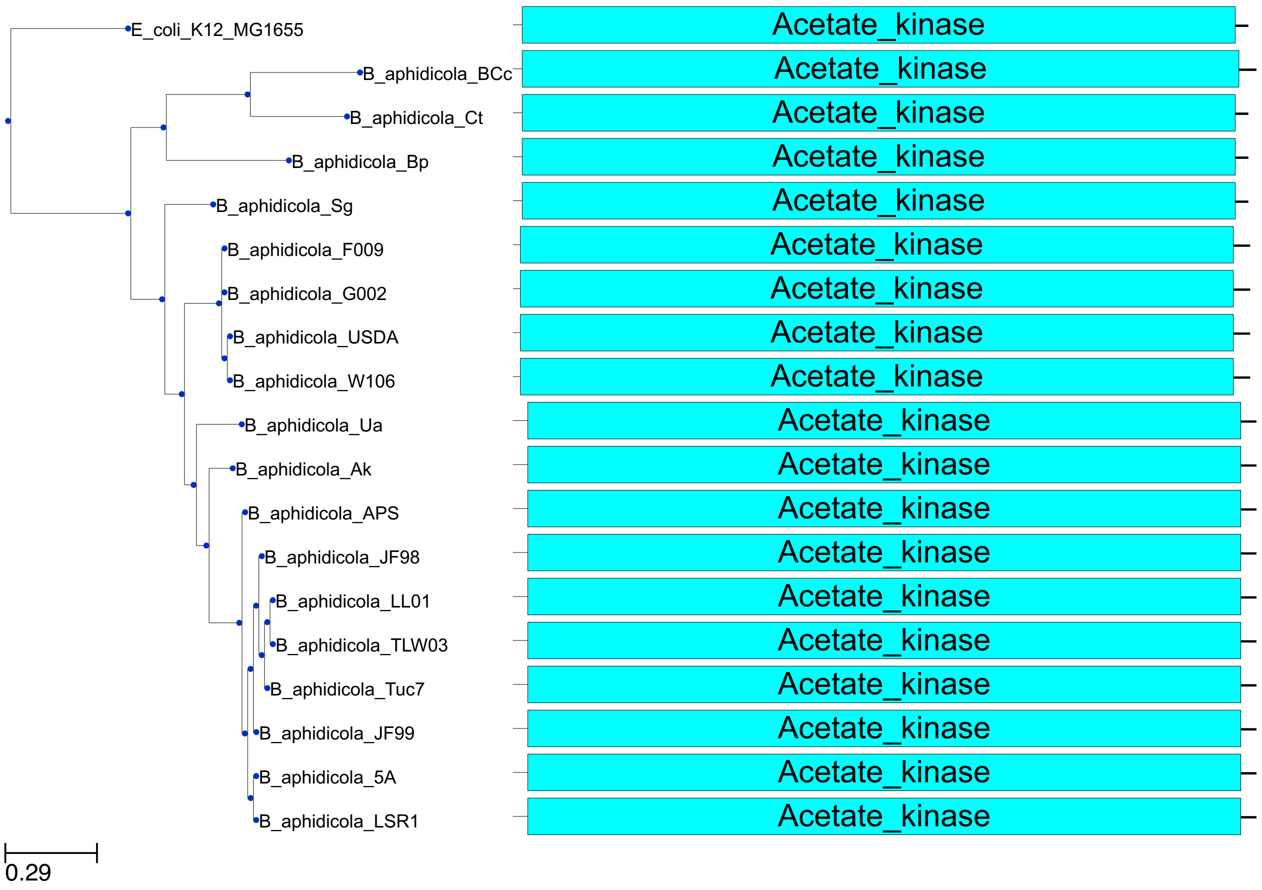

ackA
NP_416799.1
acetate kinase A and propionate kinase 2

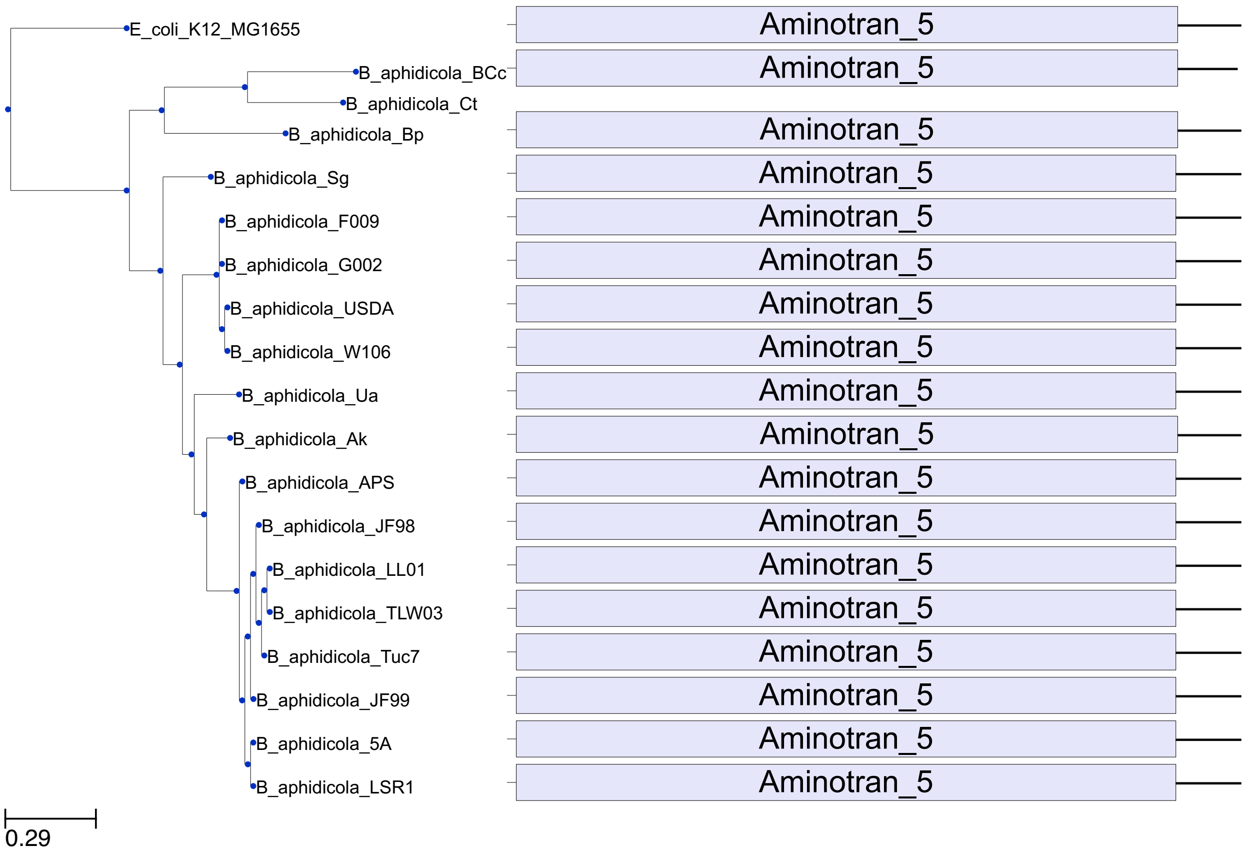

iscS
YP_026169.1
"cysteine desulfurase (tRNA sulfurtransferase), PLP-dependent"

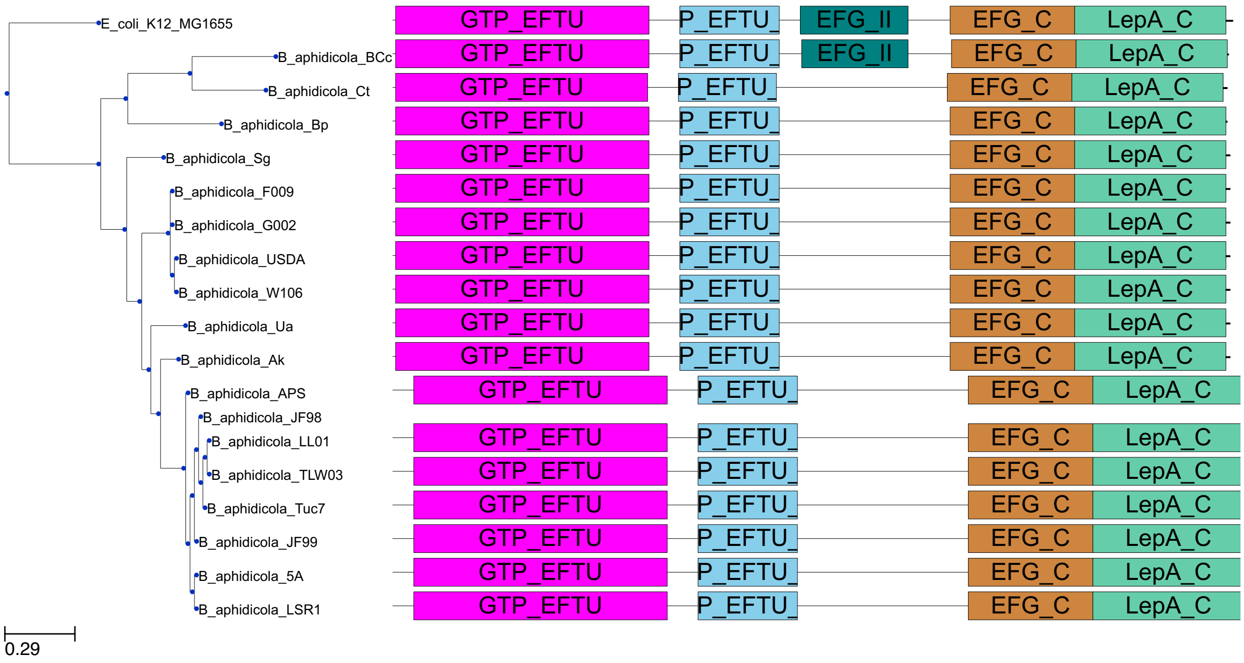

lepA

NP_417064.1

"back-translocating elongation factor EF4, GTPase"

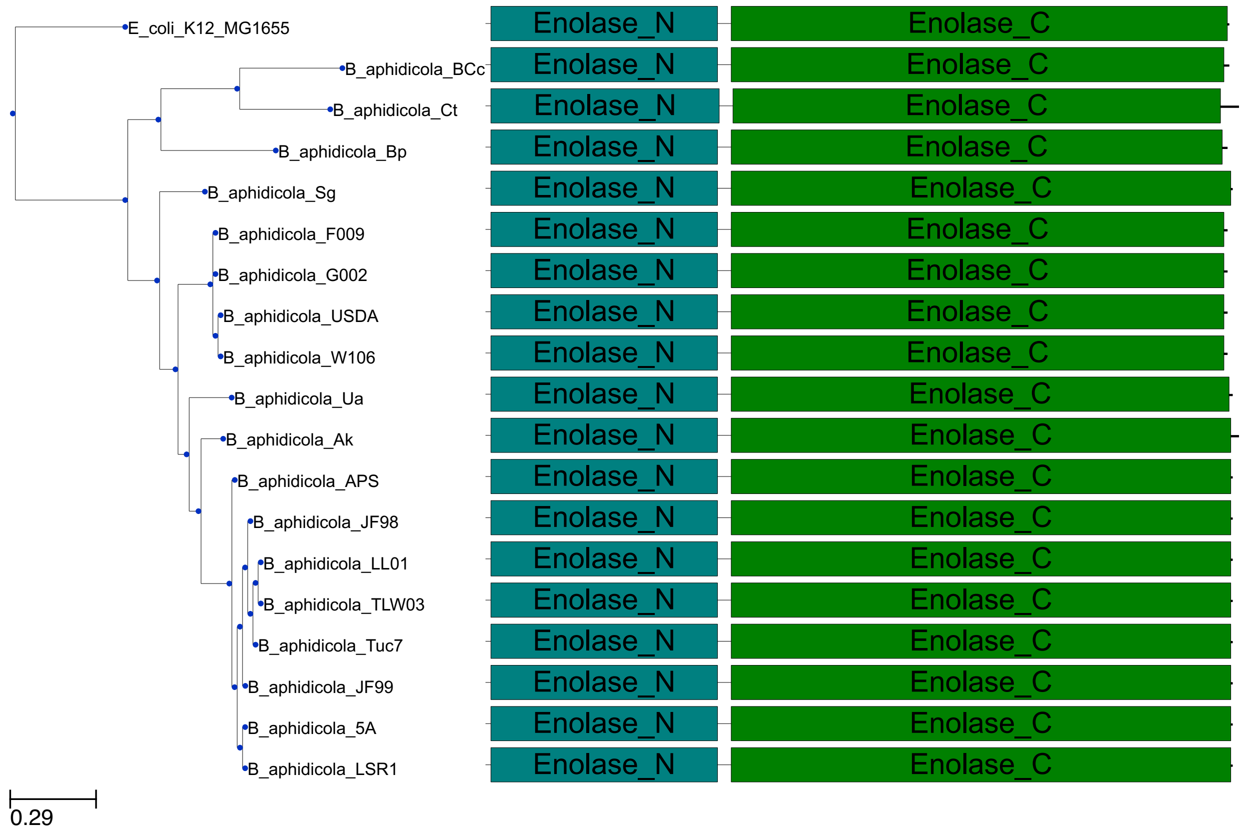

eno
NP_417259.1
enolase

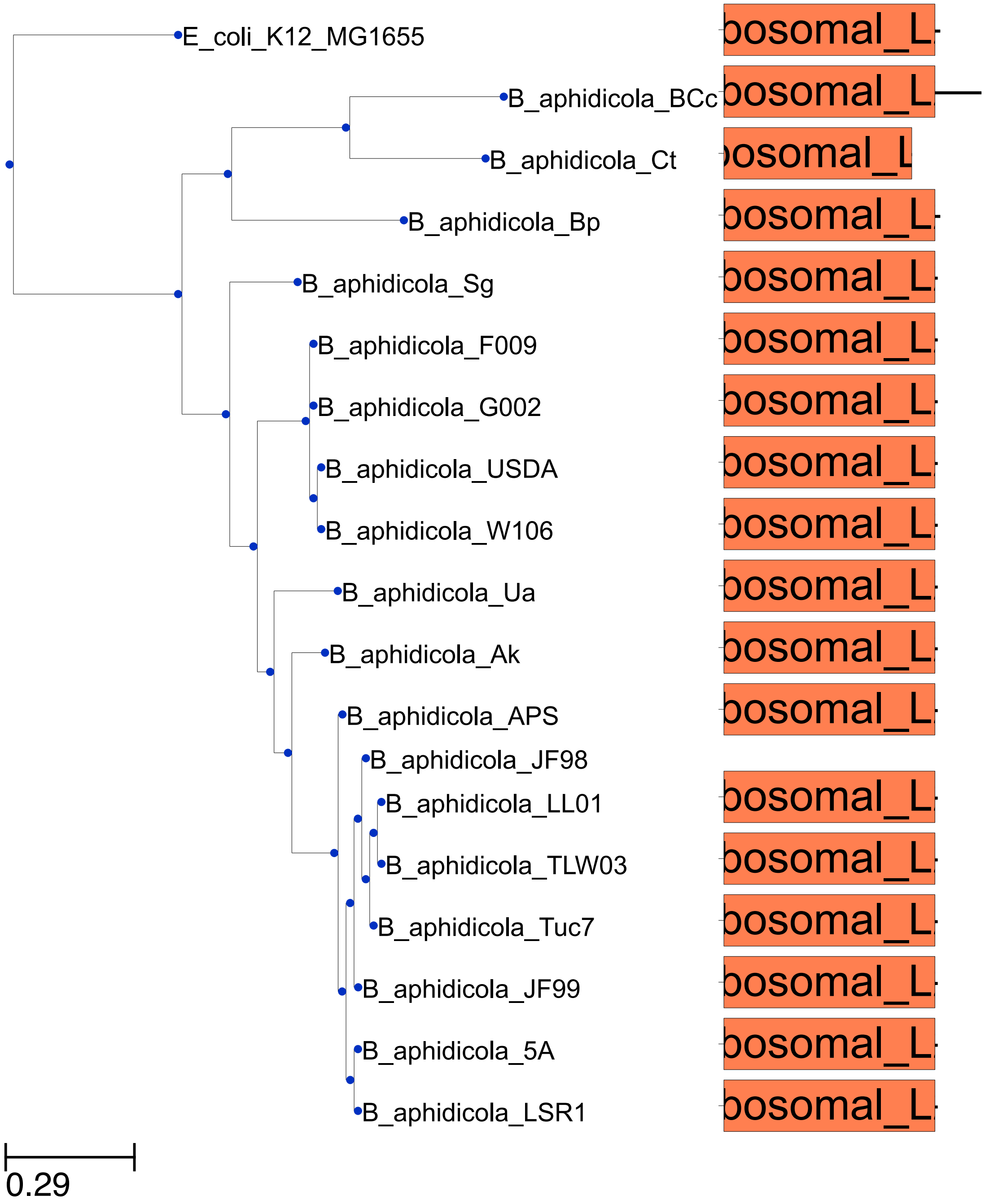

rpmA
NP_417652.1
50S ribosomal subunit protein L27

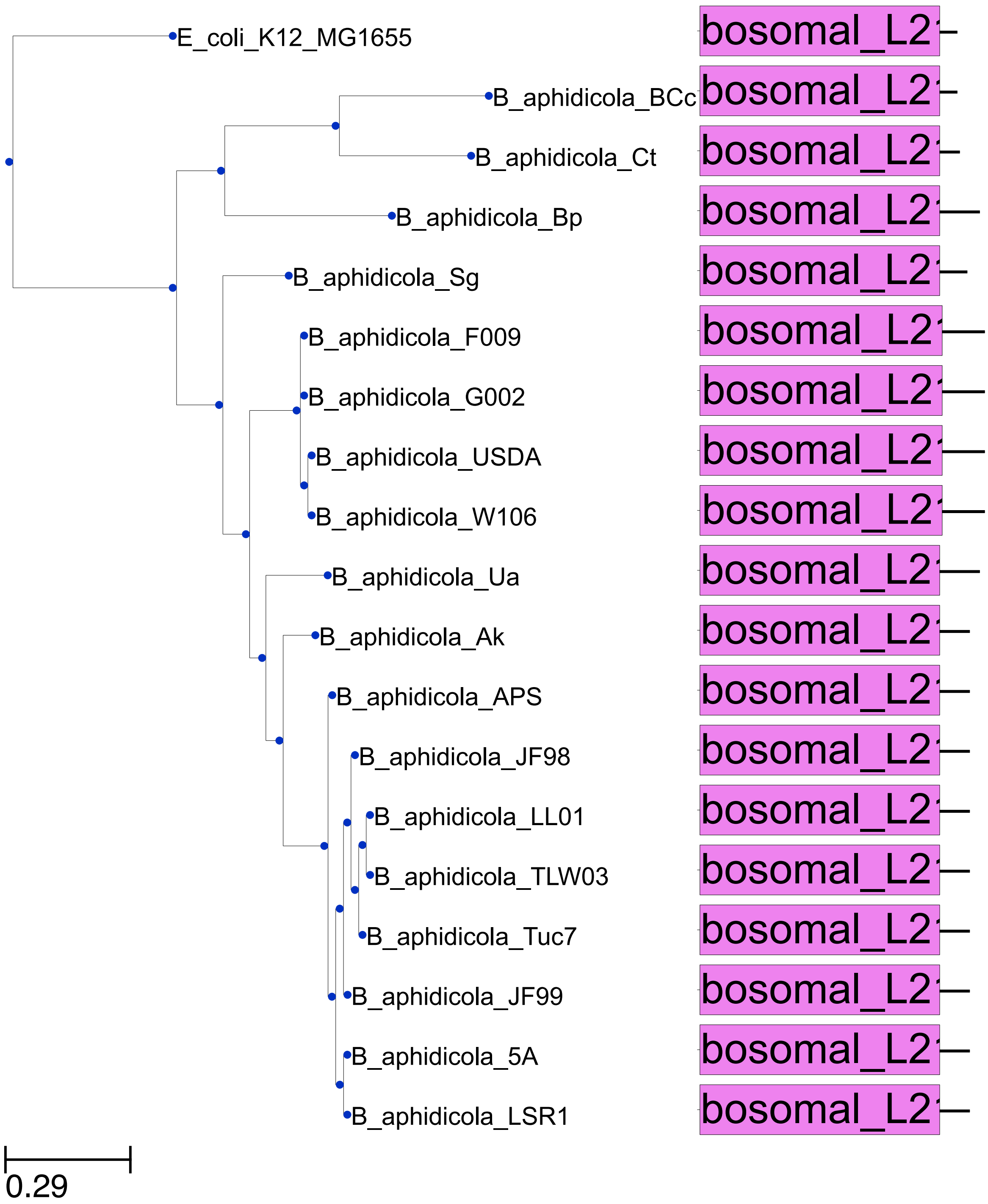

rplU
NP_417653.1
50S ribosomal subunit protein L21

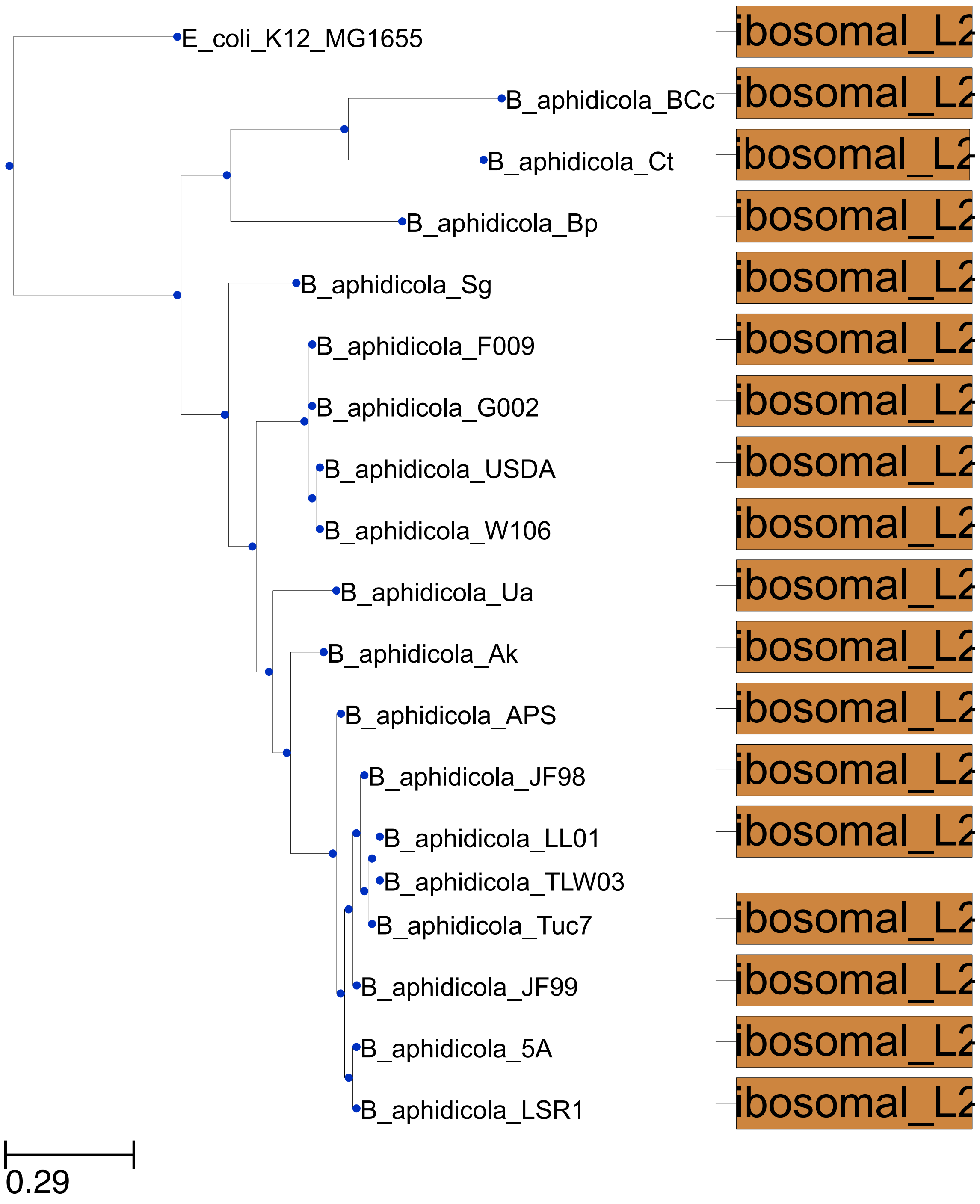

rplW
NP_417777.1
50S ribosomal subunit protein L23

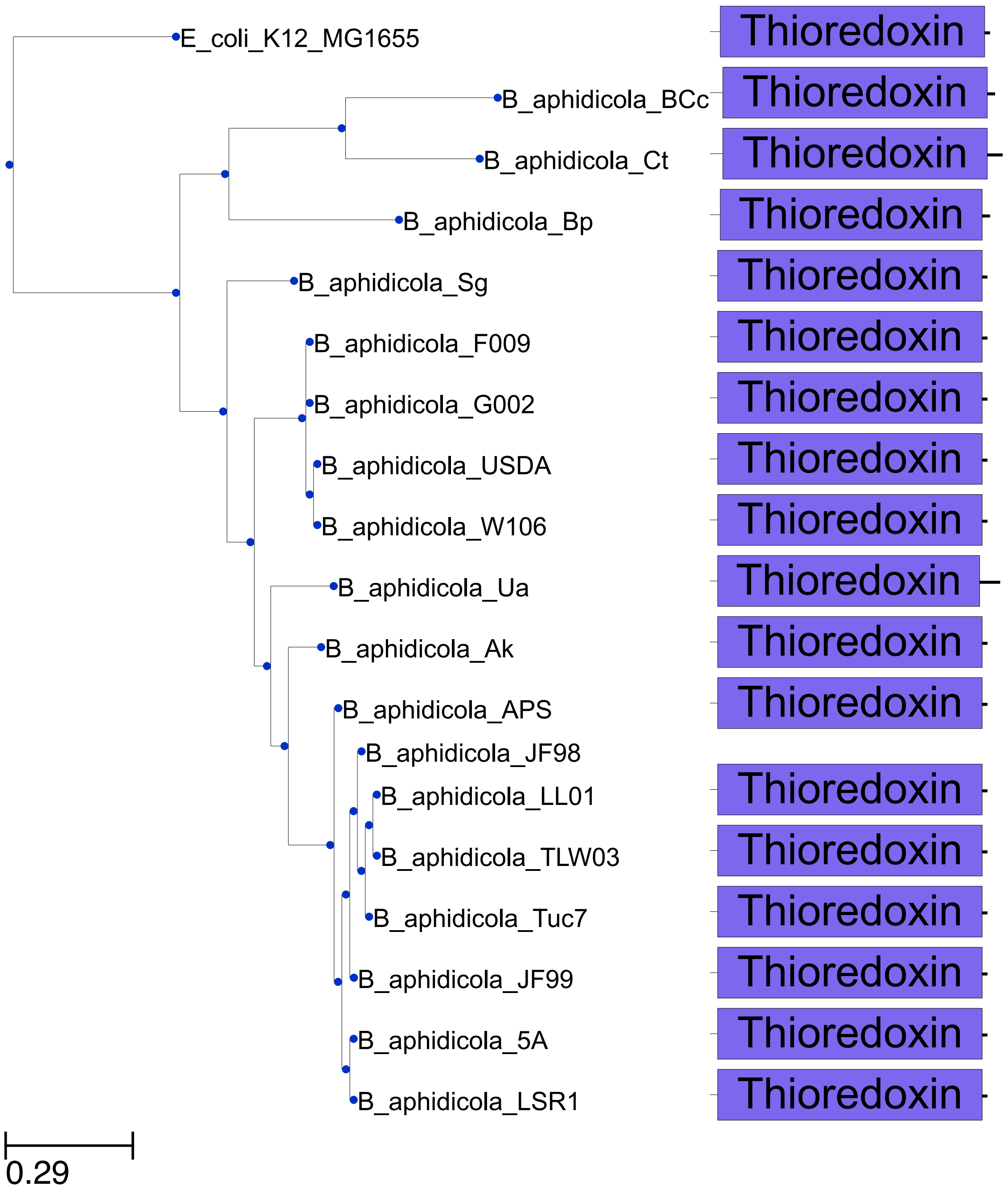

trxA
NP_418228.2
thioredoxin 1

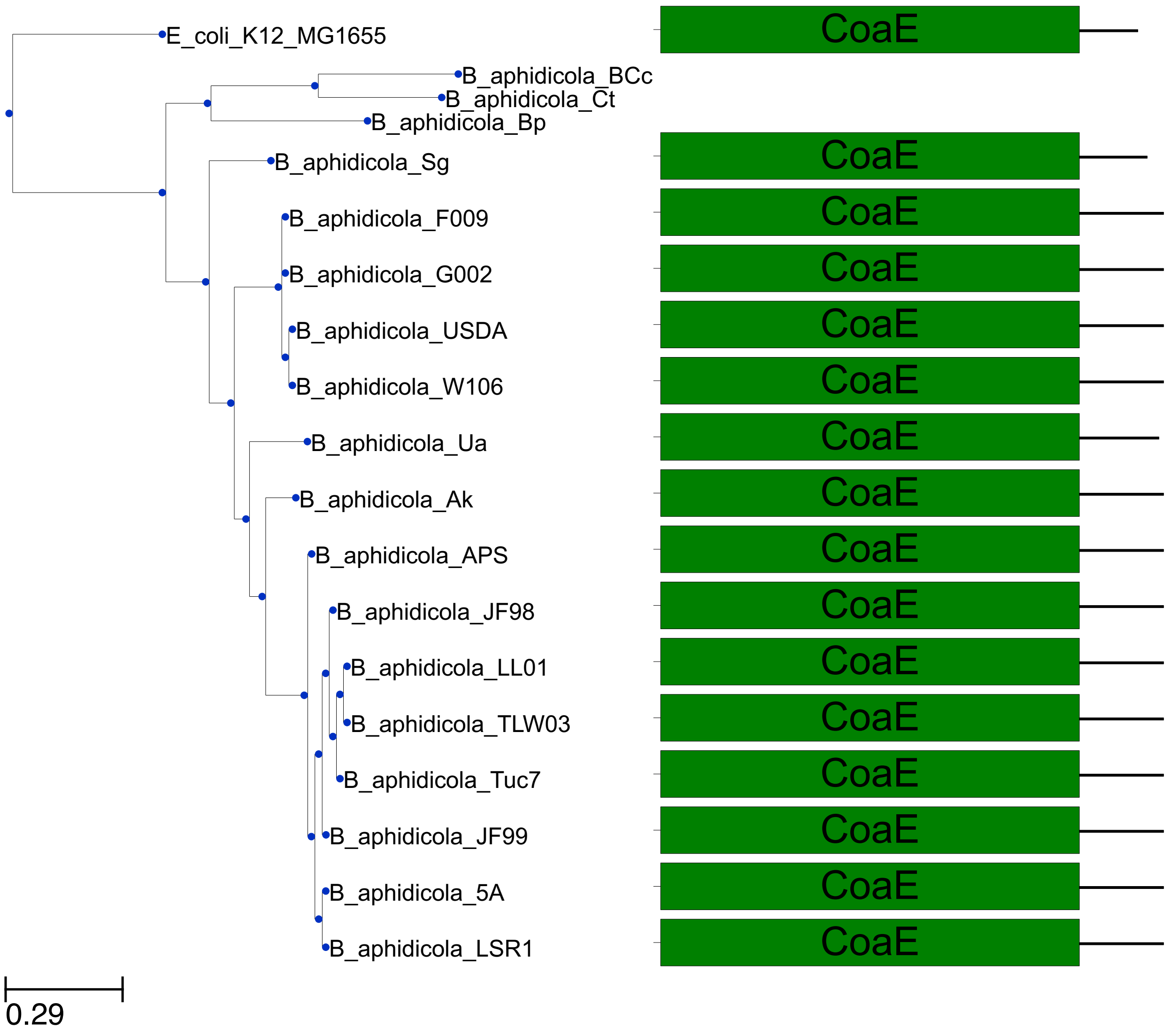

coaE
NP_414645.1
dephospho-CoA kinase

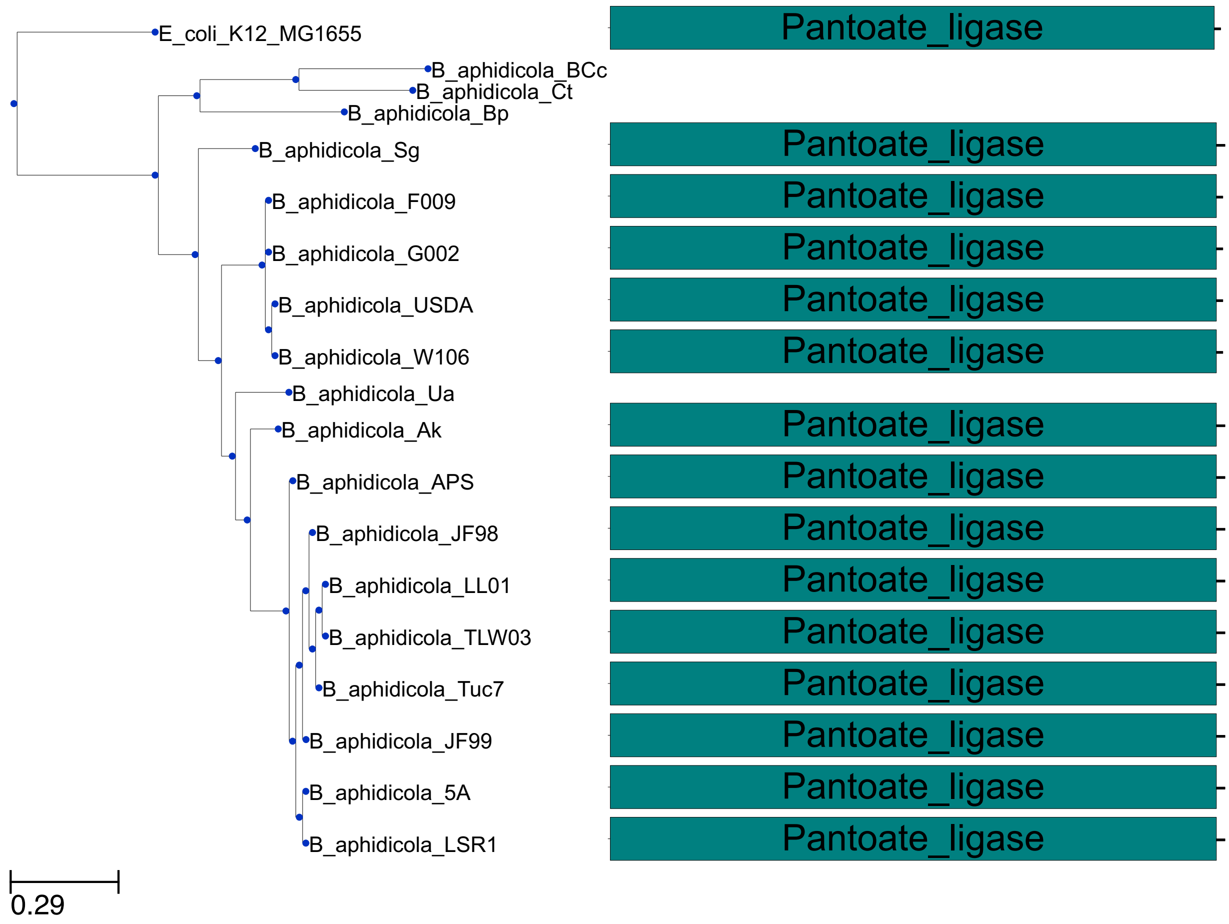

panC
NP_414675.1
pantothenate synthetase

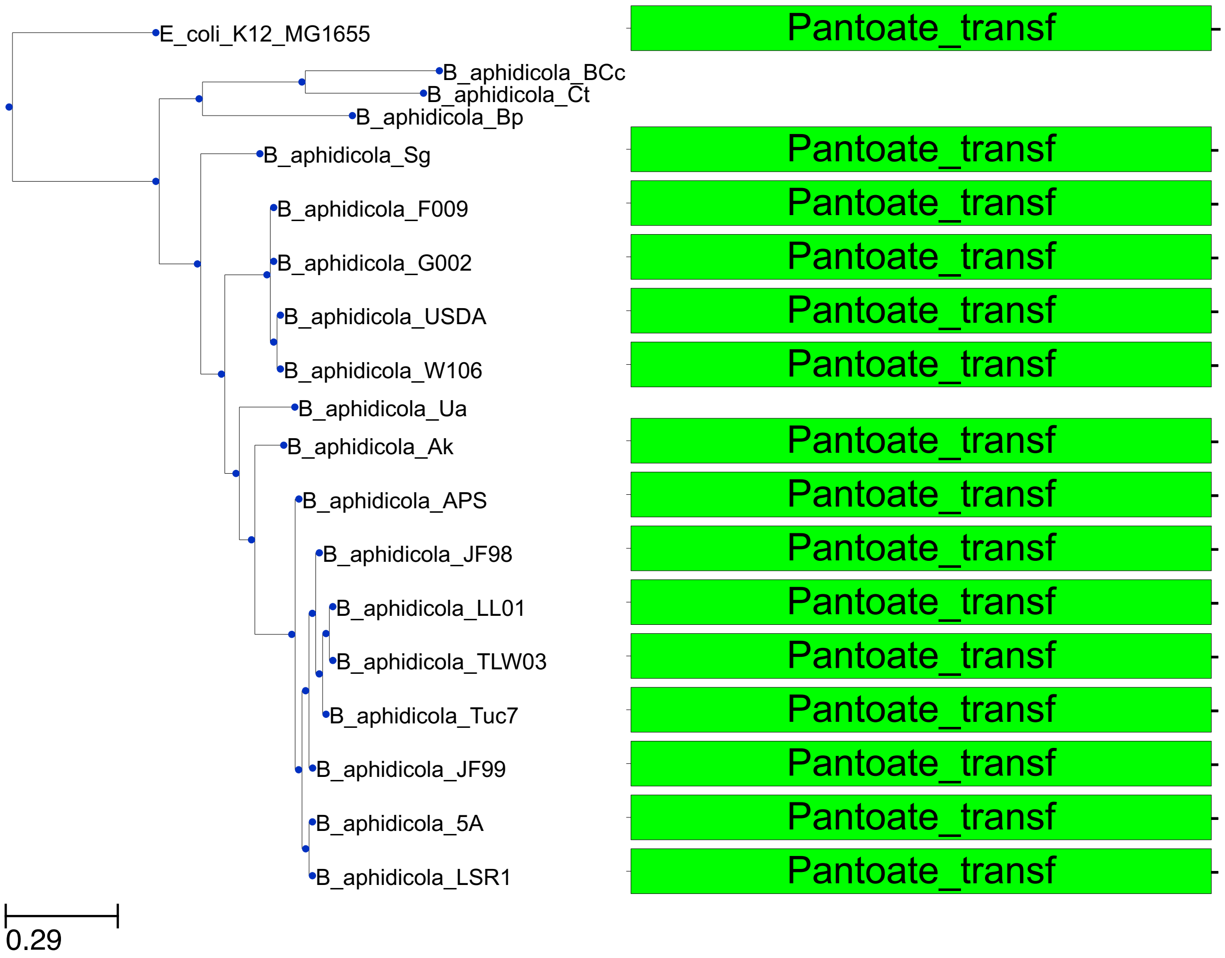

panB
NP_414676.1
3-methyl-2-oxobutanoate hydroxymethyltransferase

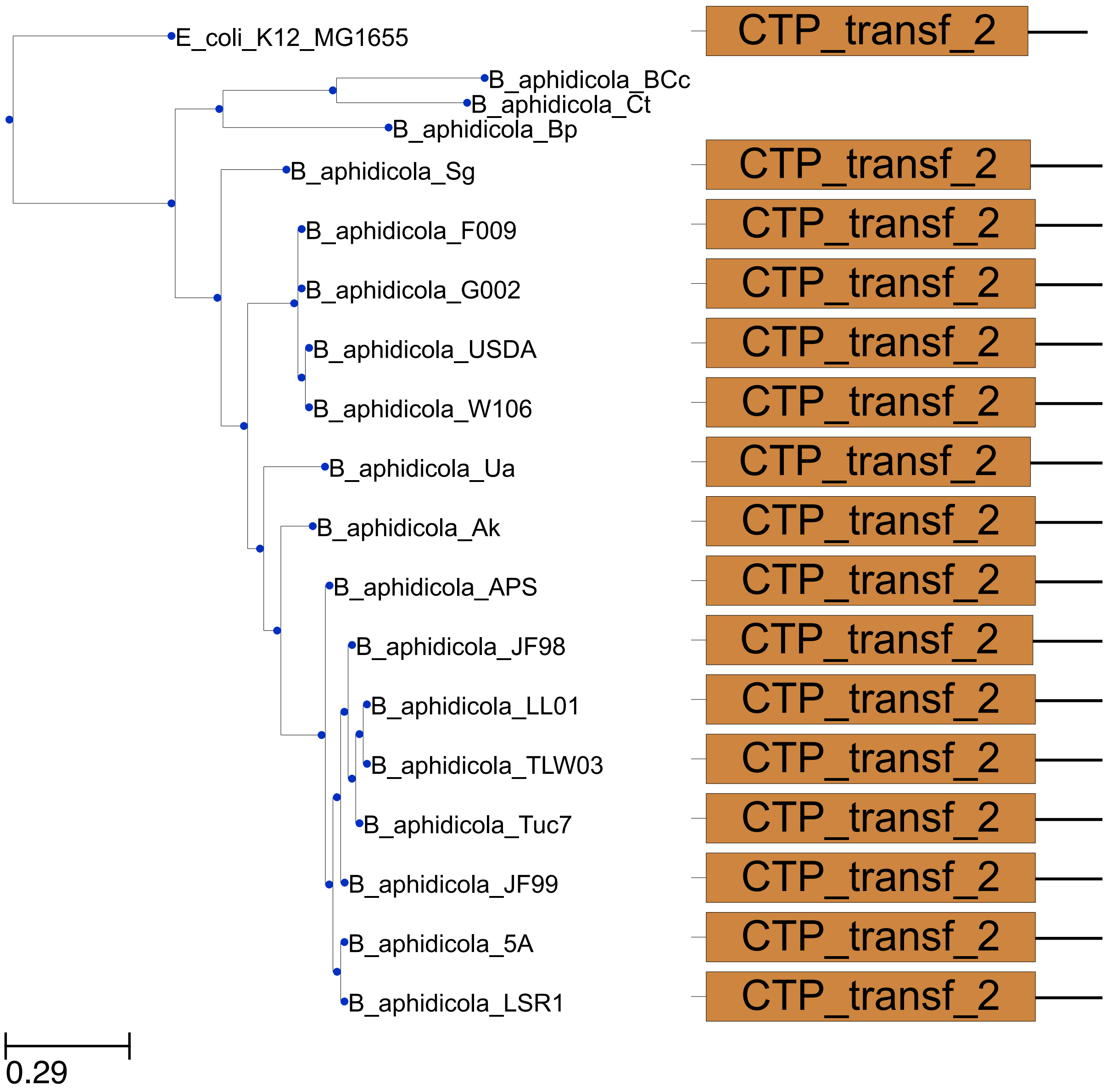

coaD
NP_418091.1
pantetheine-phosphate adenylyltransferase

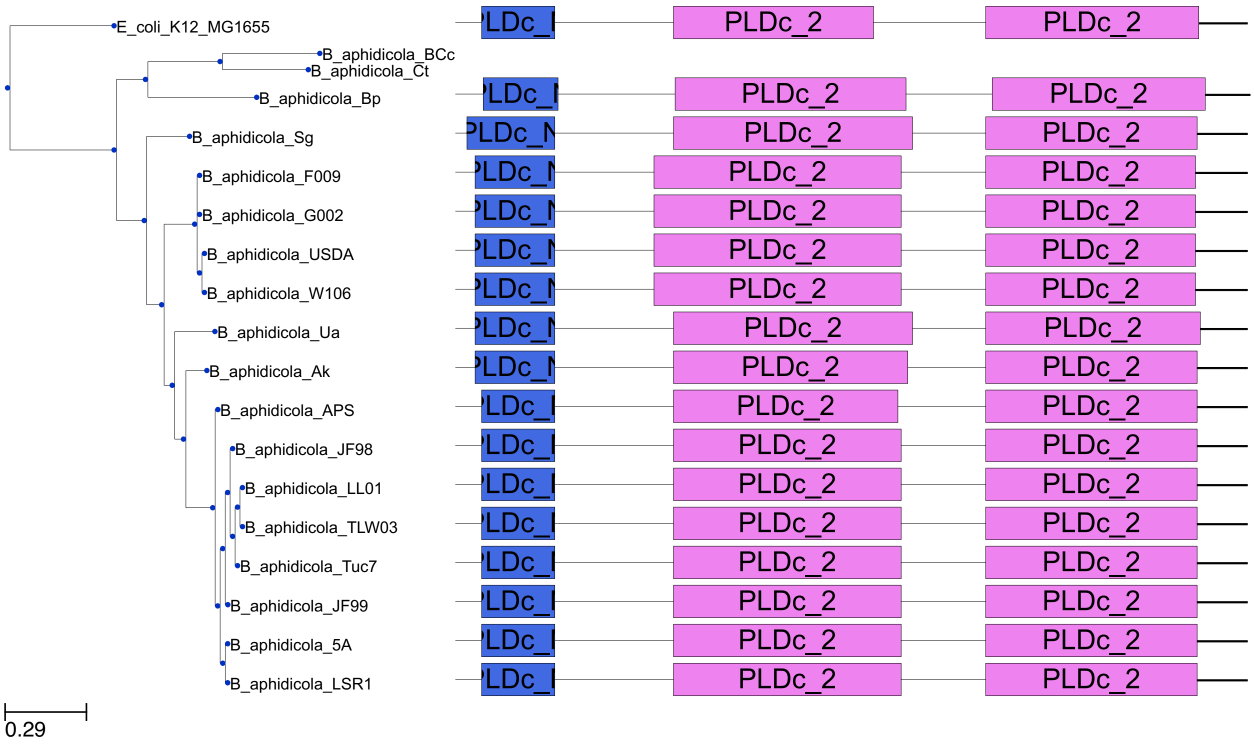

cls

NP_415765.1

cardiolipin synthase 1

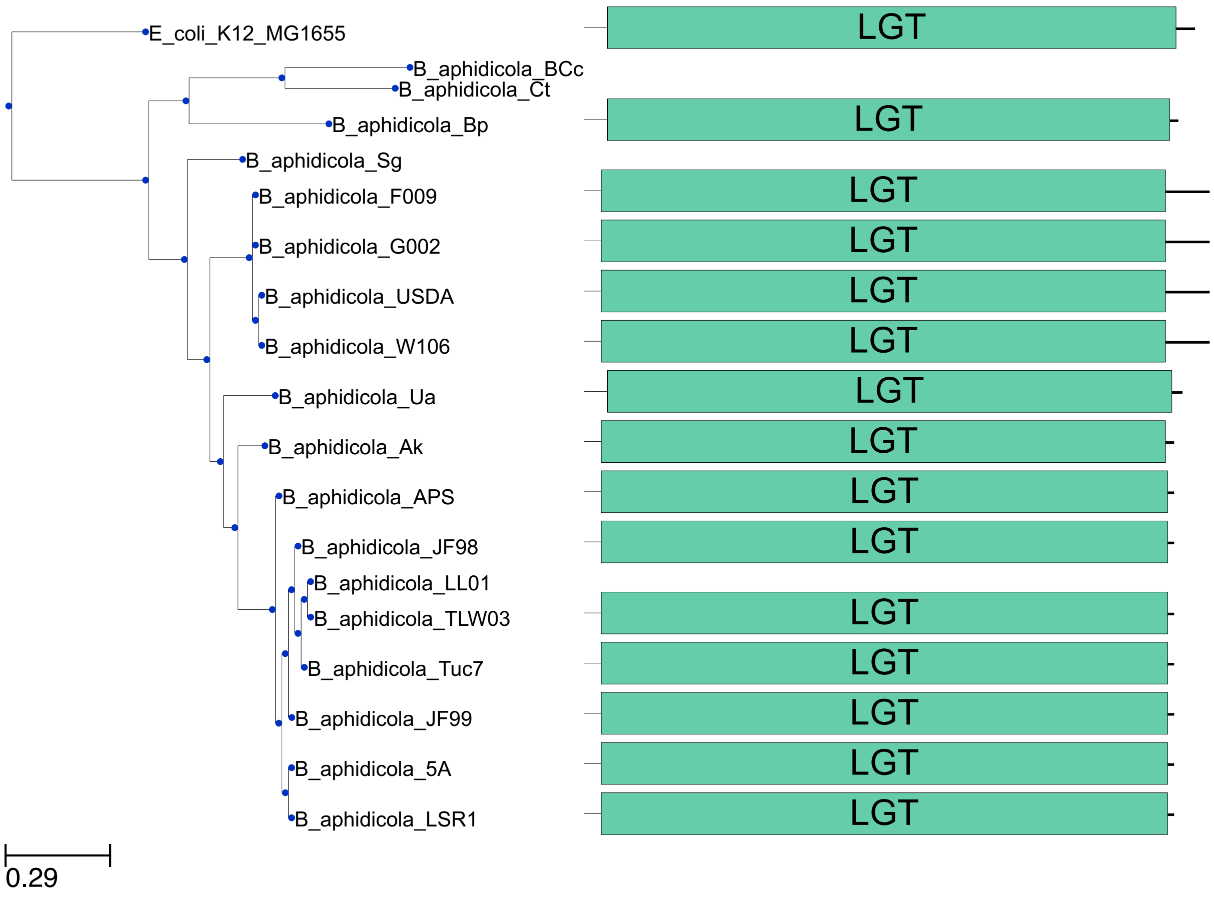

lgt
NP_417305.1
phosphatidylglycerol-prolipoprotein diacylglyceryl transferase

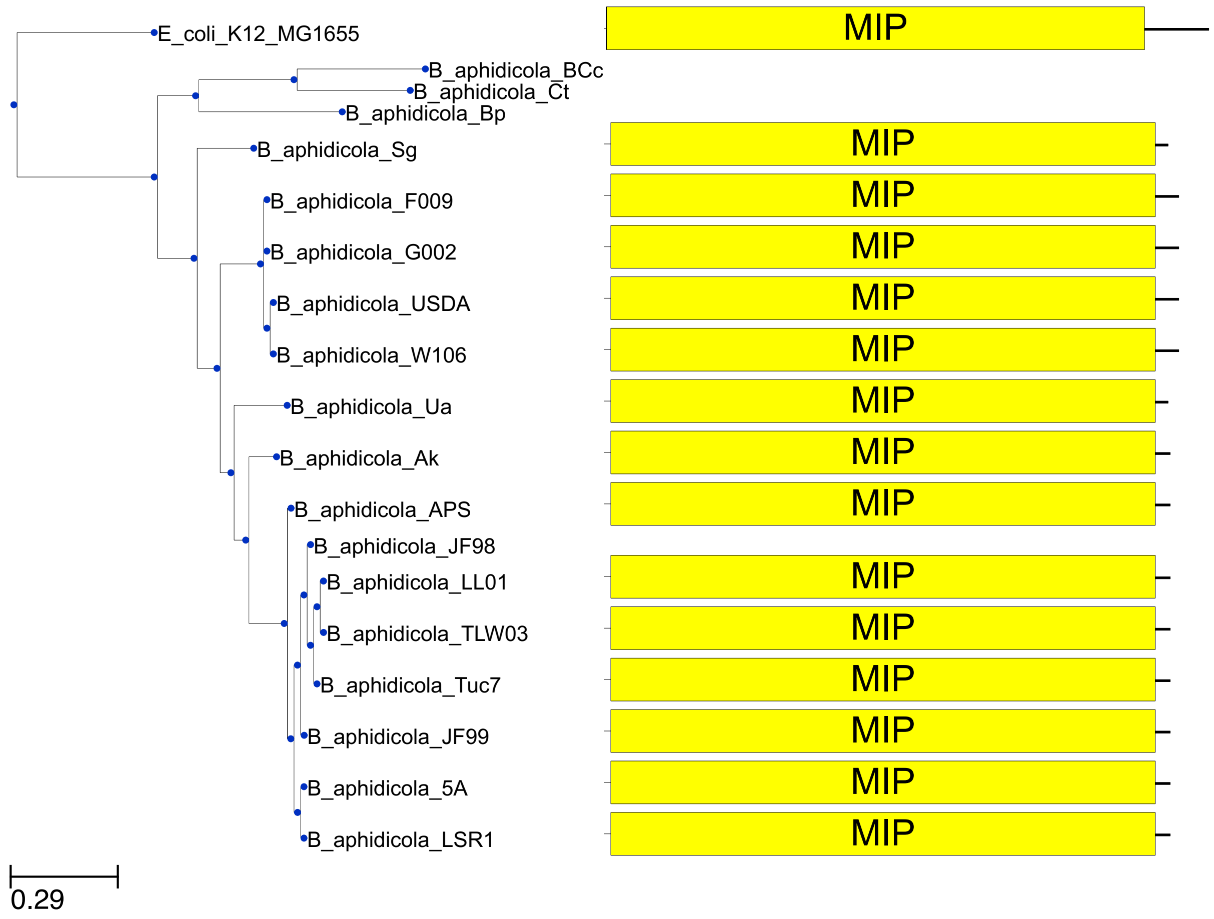

glpF
NP_418362.1
glycerol facilitator

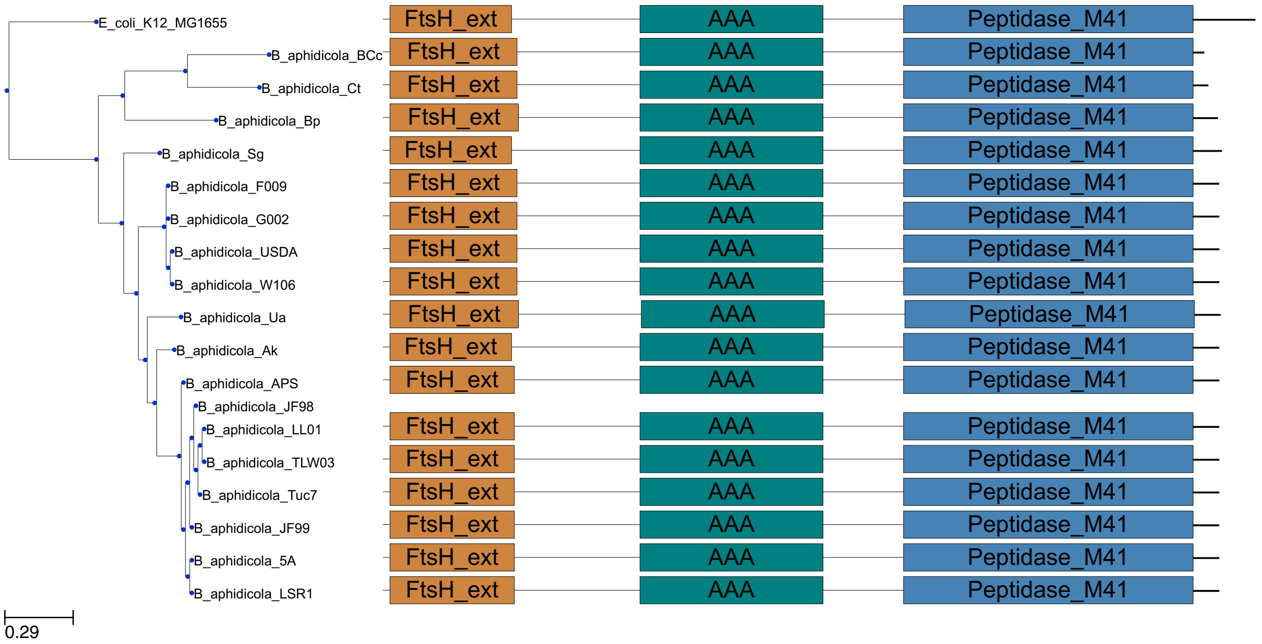

ftsH

NP_417645.1

"protease, ATP-dependent zinc-metallo"

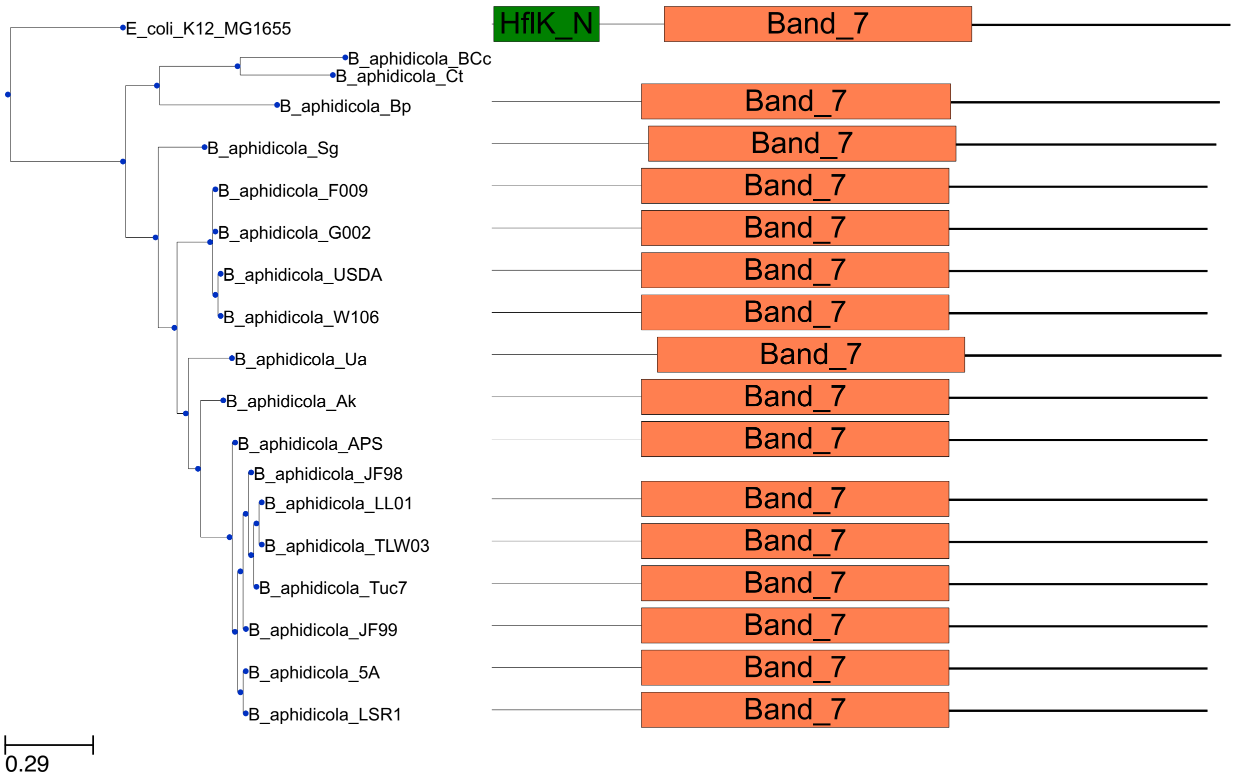

hflK
NP_418595.1
modulator for HflB protease specific for phage lambda cII repressor

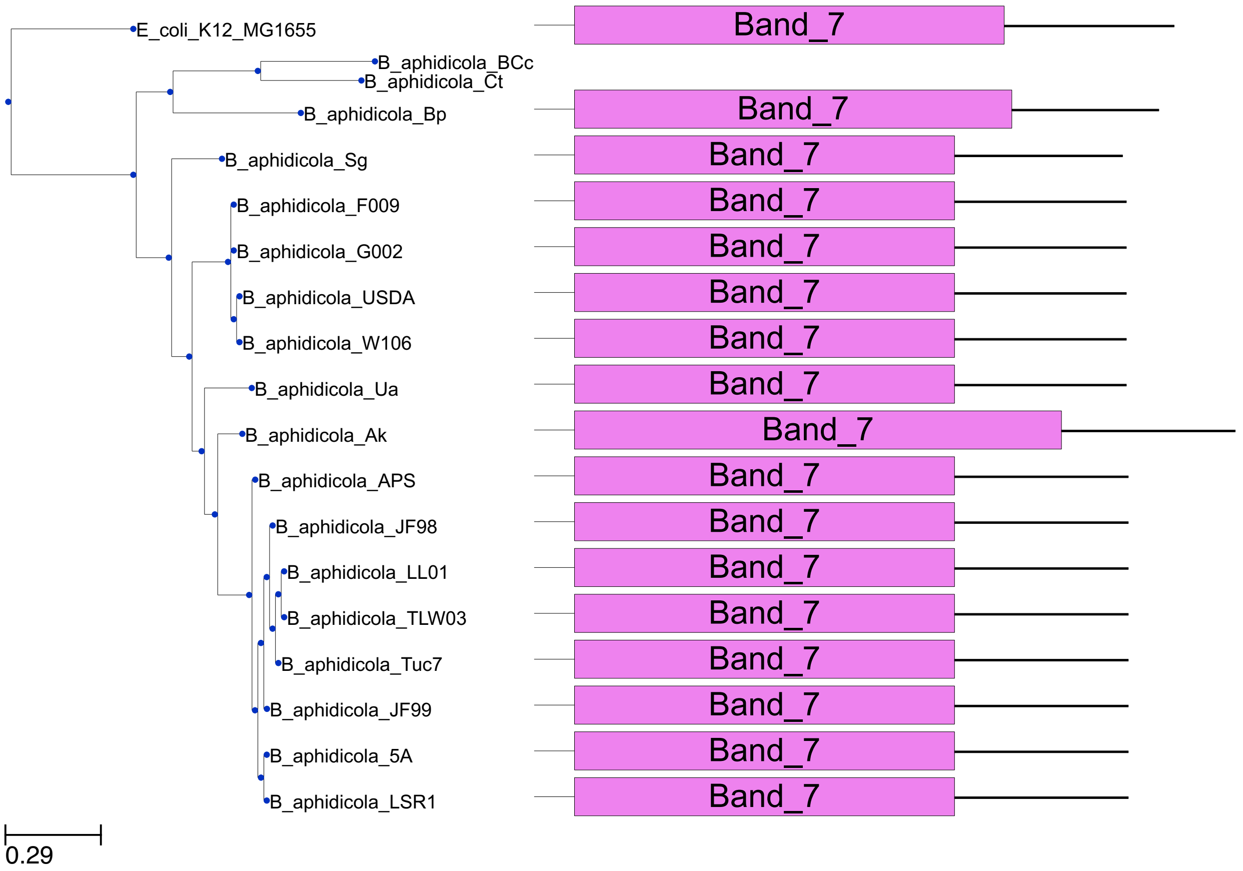

hflC
NP_418596.1
HflB protease modulator specific for phage lambda cII repressor

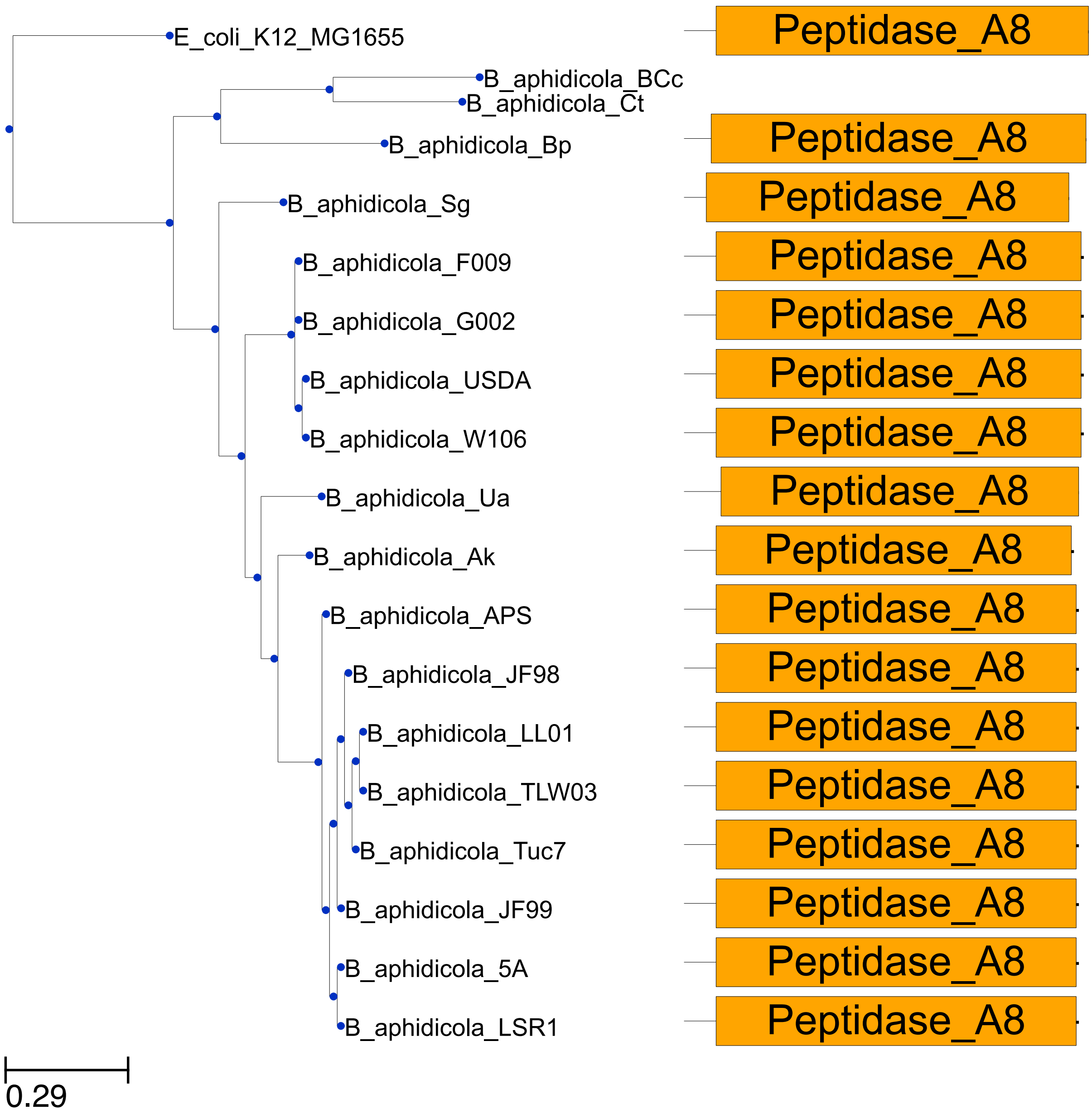

lspA
NP_414568.1
prolipoprotein signal peptidase (signal peptidase II)

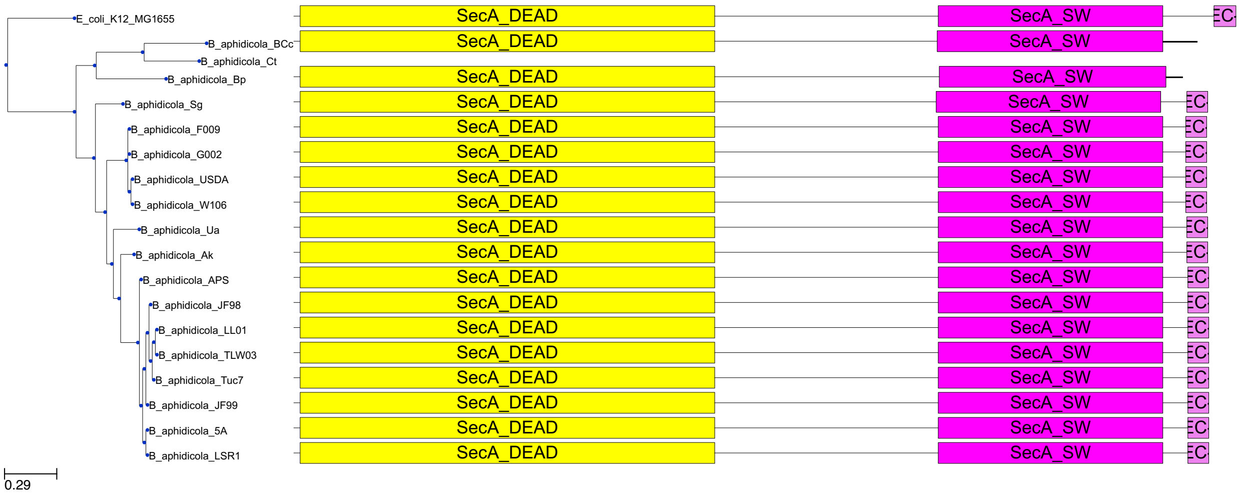

secA
NP_414640.1
"preprotein translocase subunit, ATPase"

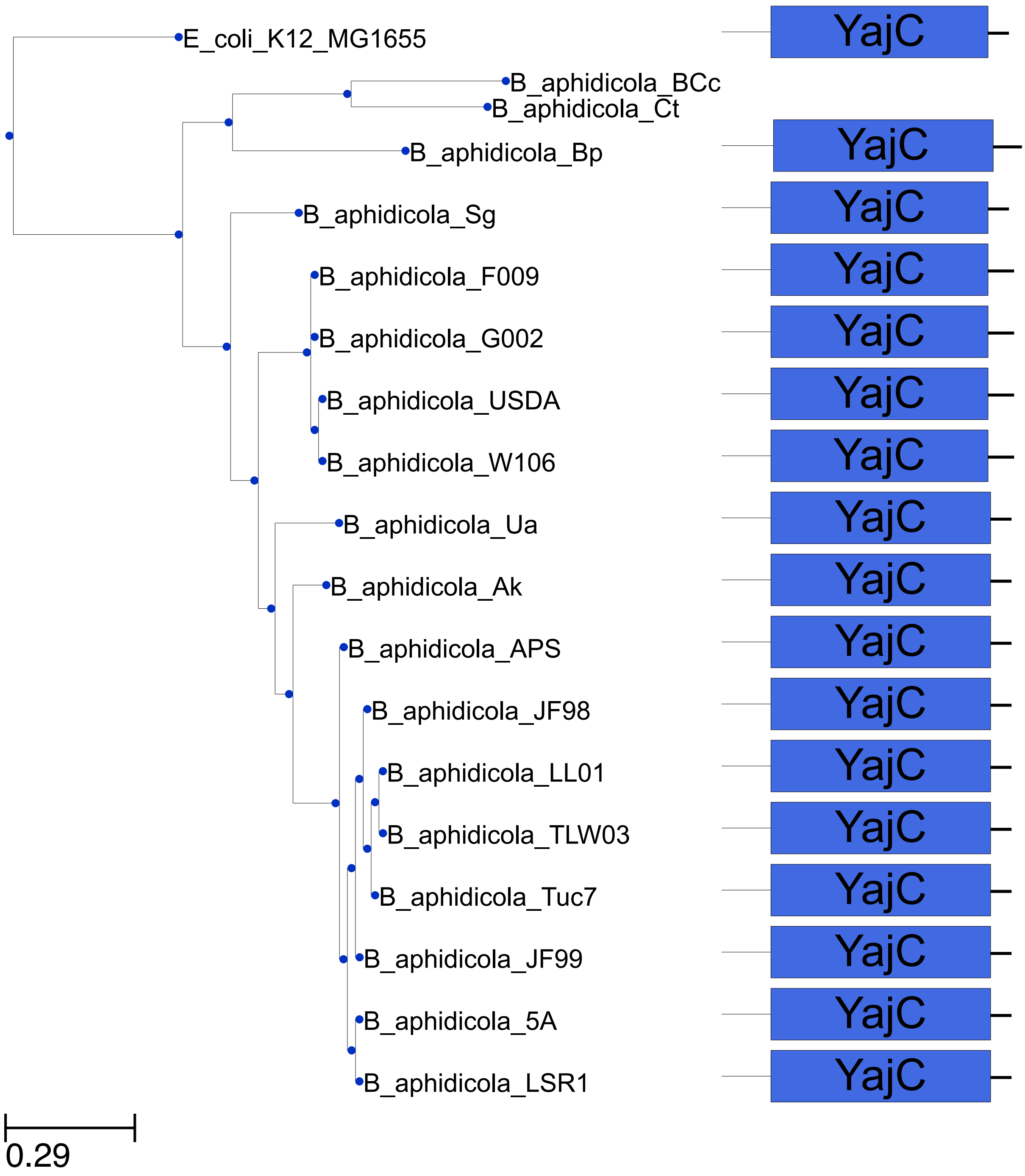

yajC
NP_414941.1
SecYEG protein translocase auxillary subunit

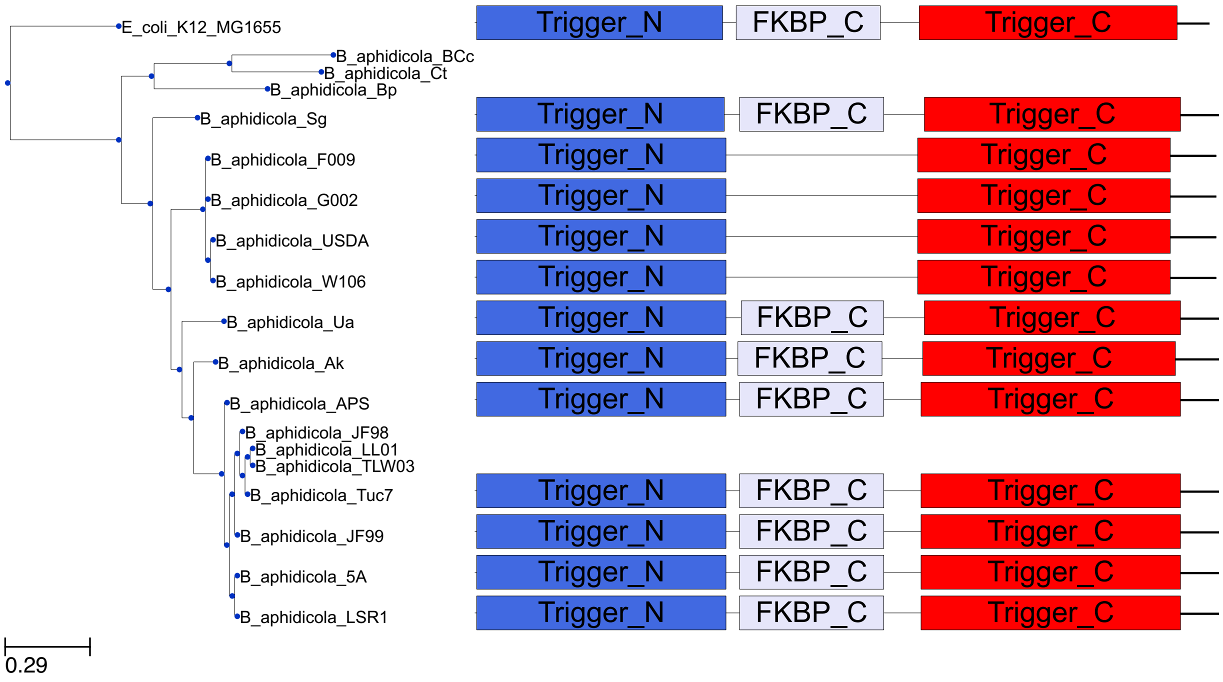

tig

NP_414970.1

peptidyl-prolyl cis/trans isomerase (trigger factor)

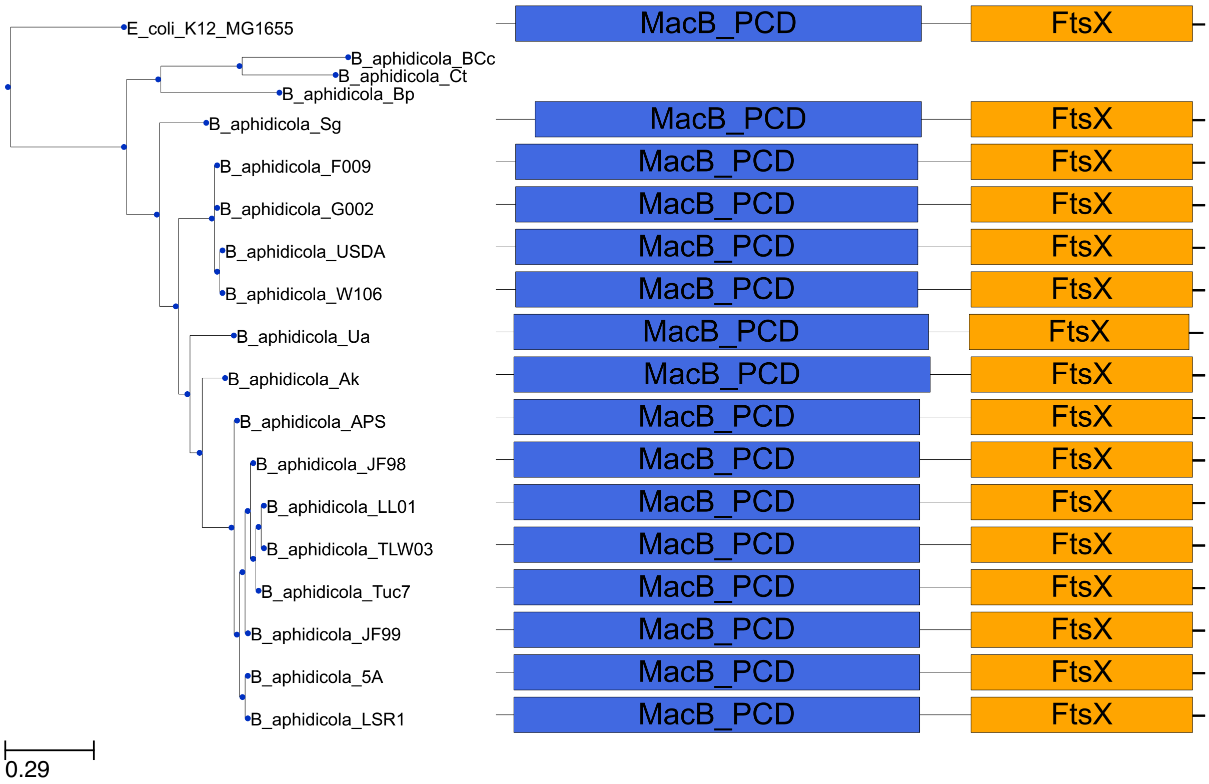

lolC

NP_415634.1

lipoprotein-releasing system transmembrane protein

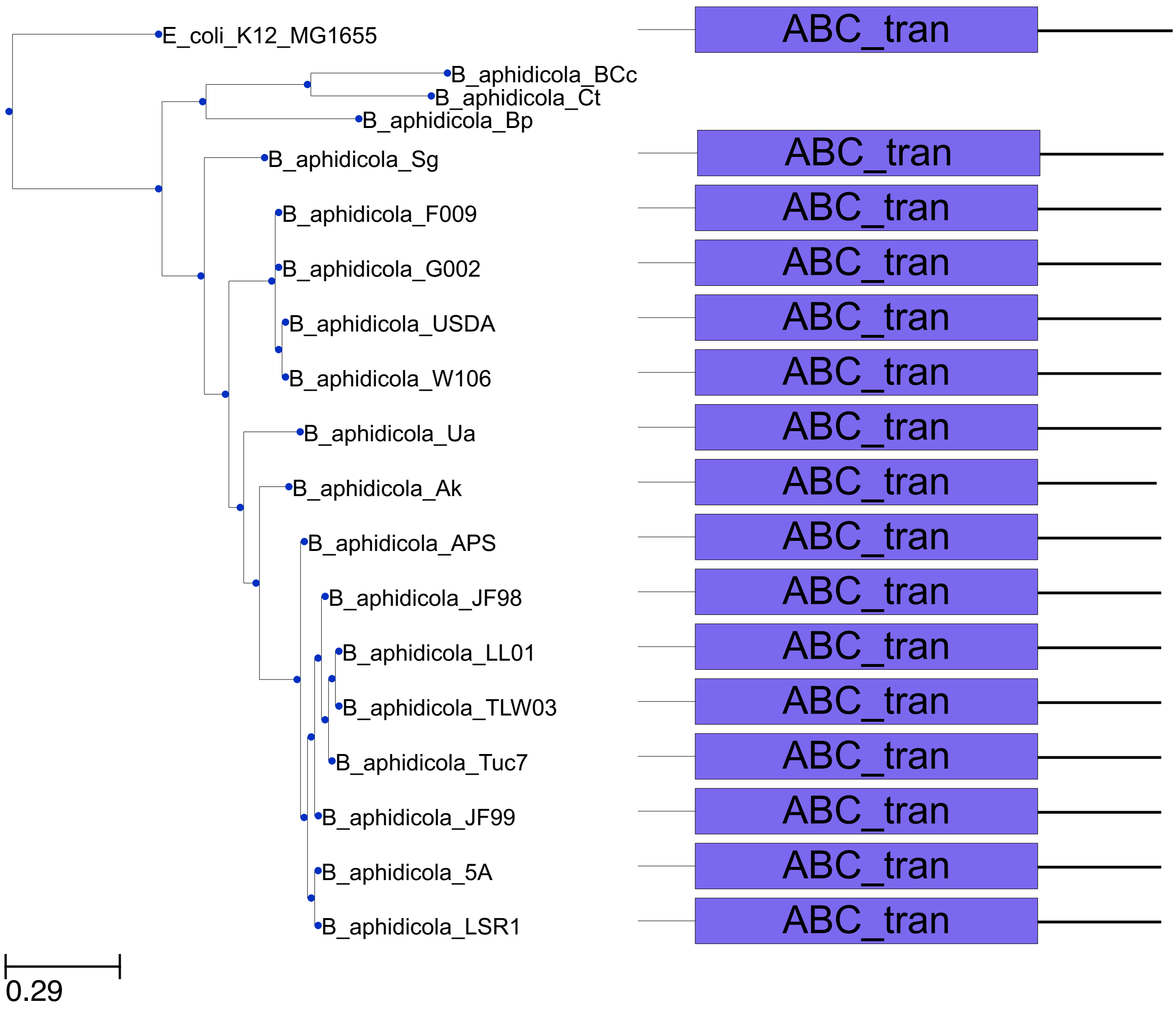

lolD

NP_415635.4

outer membrane-specific lipoprotein transporter subunit

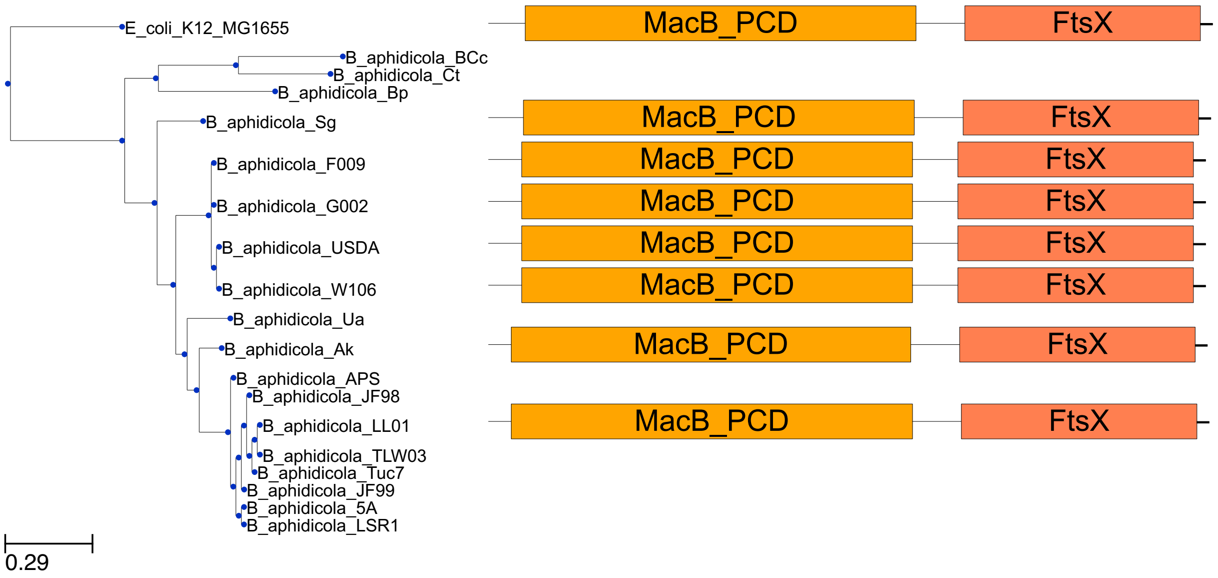

lolE

NP_415636.1

lipoprotein-releasing system transmembrane protein

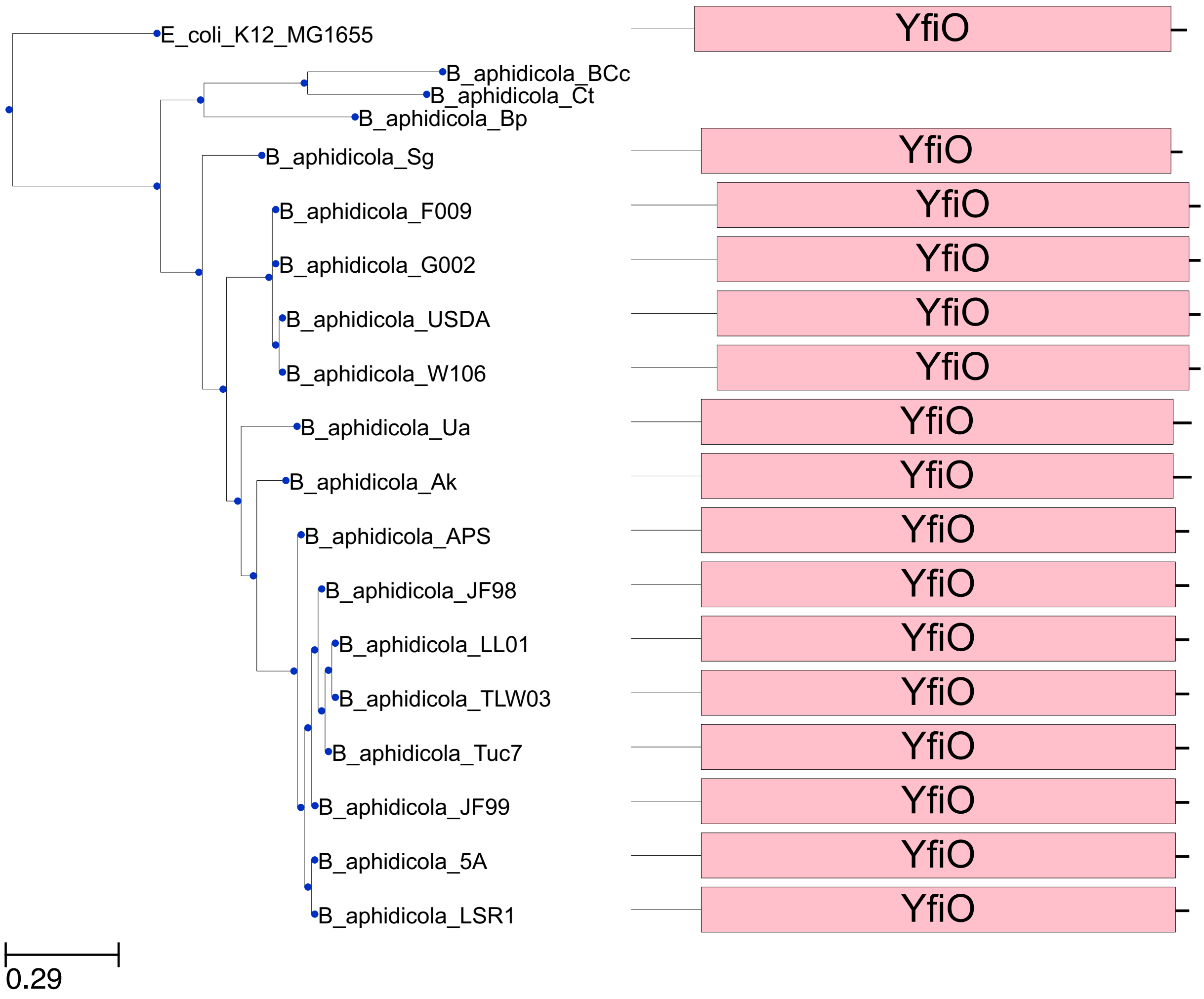

yfiO
NP_417086.1
BamABCDE complex OM biogenesis lipoprotein

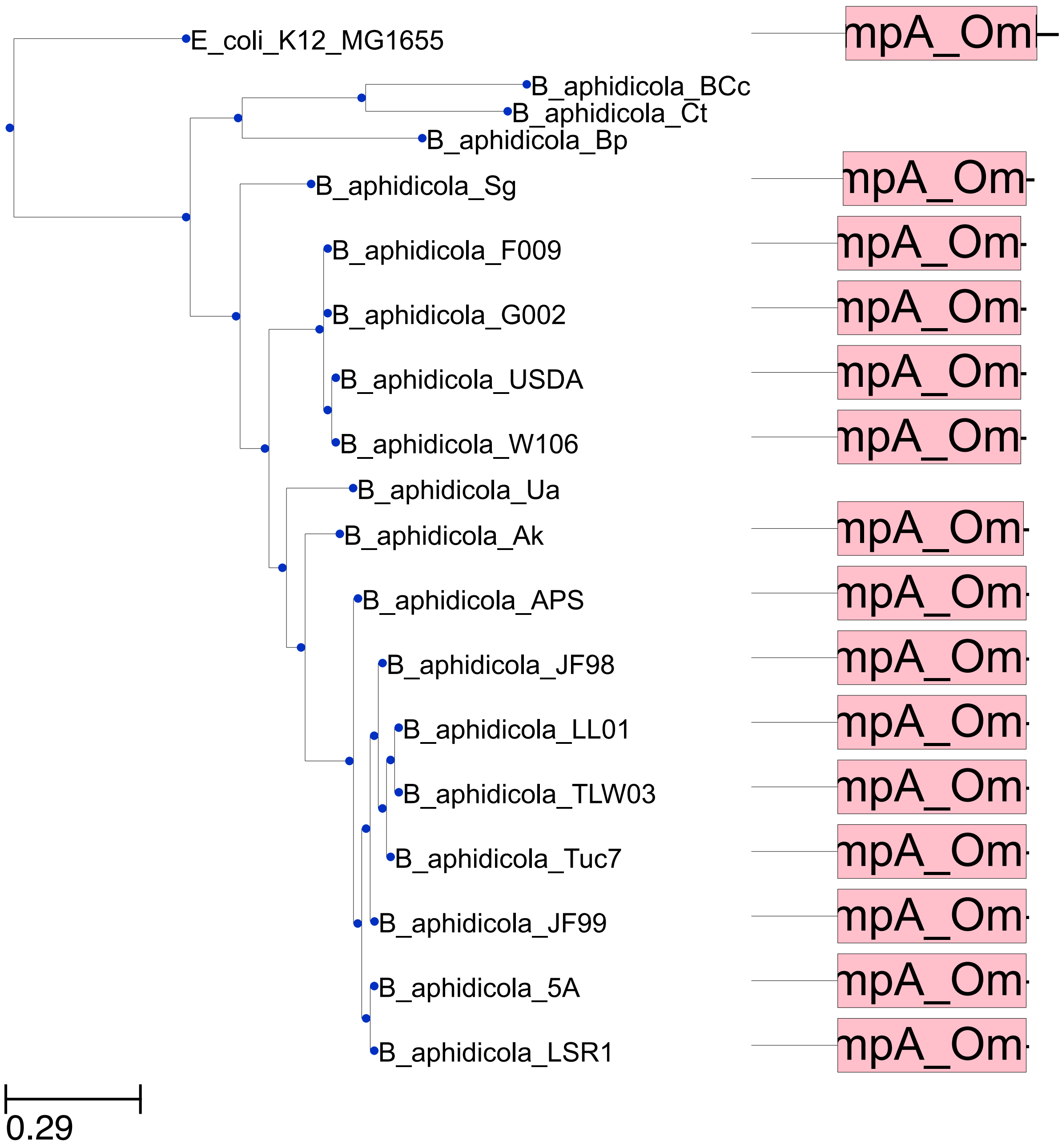

smpA
NP_417107.2
lipoprotein component of BamABCDE OM biogenesis complex

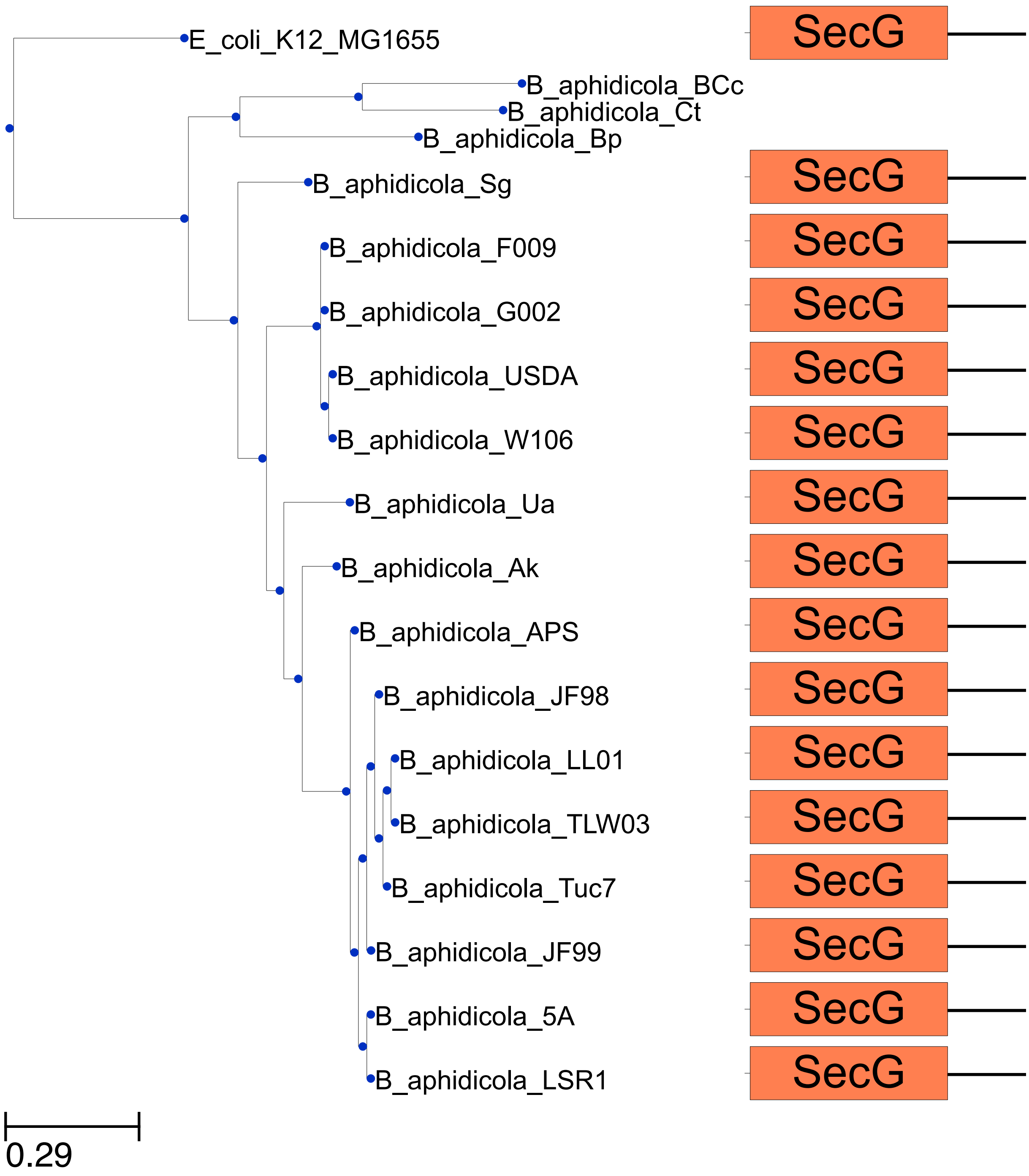

secG
NP_417642.1
preprotein translocase membrane subunit

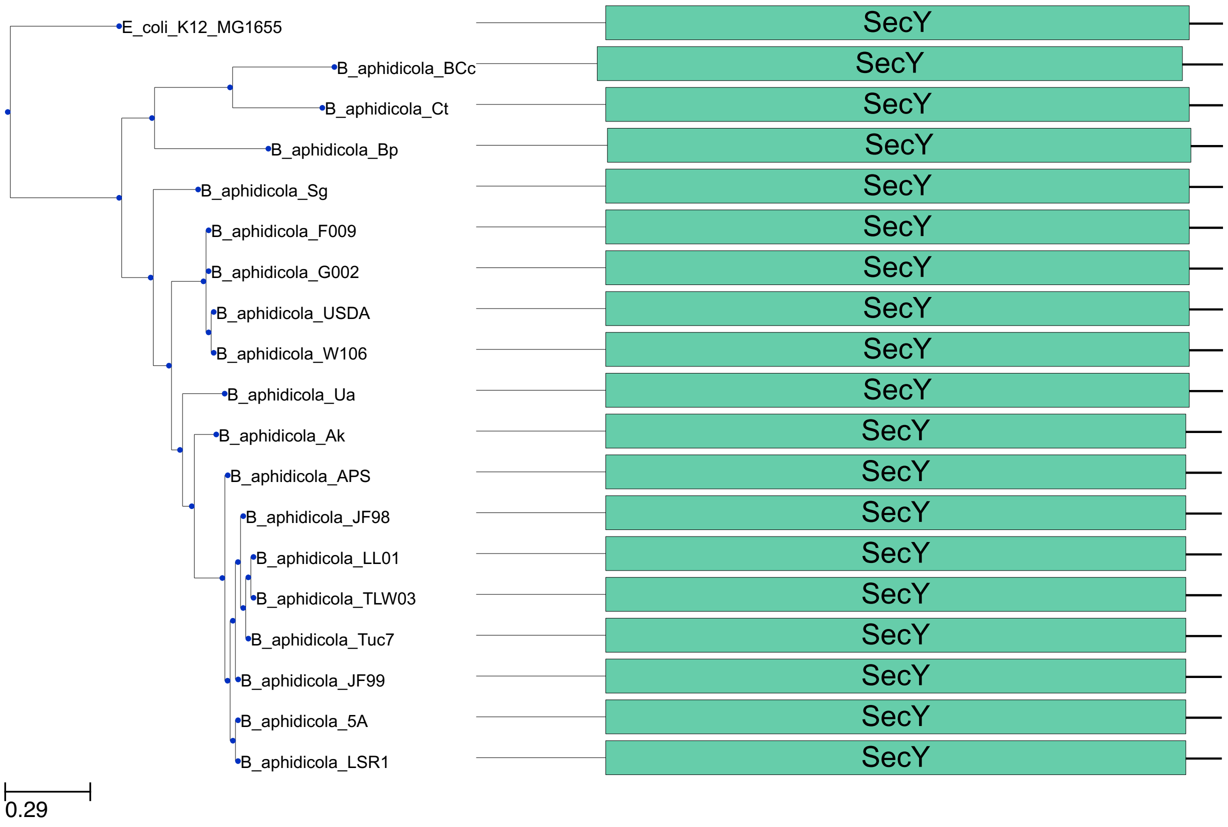

secY

NP_417759.1

preprotein translocase membrane subunit

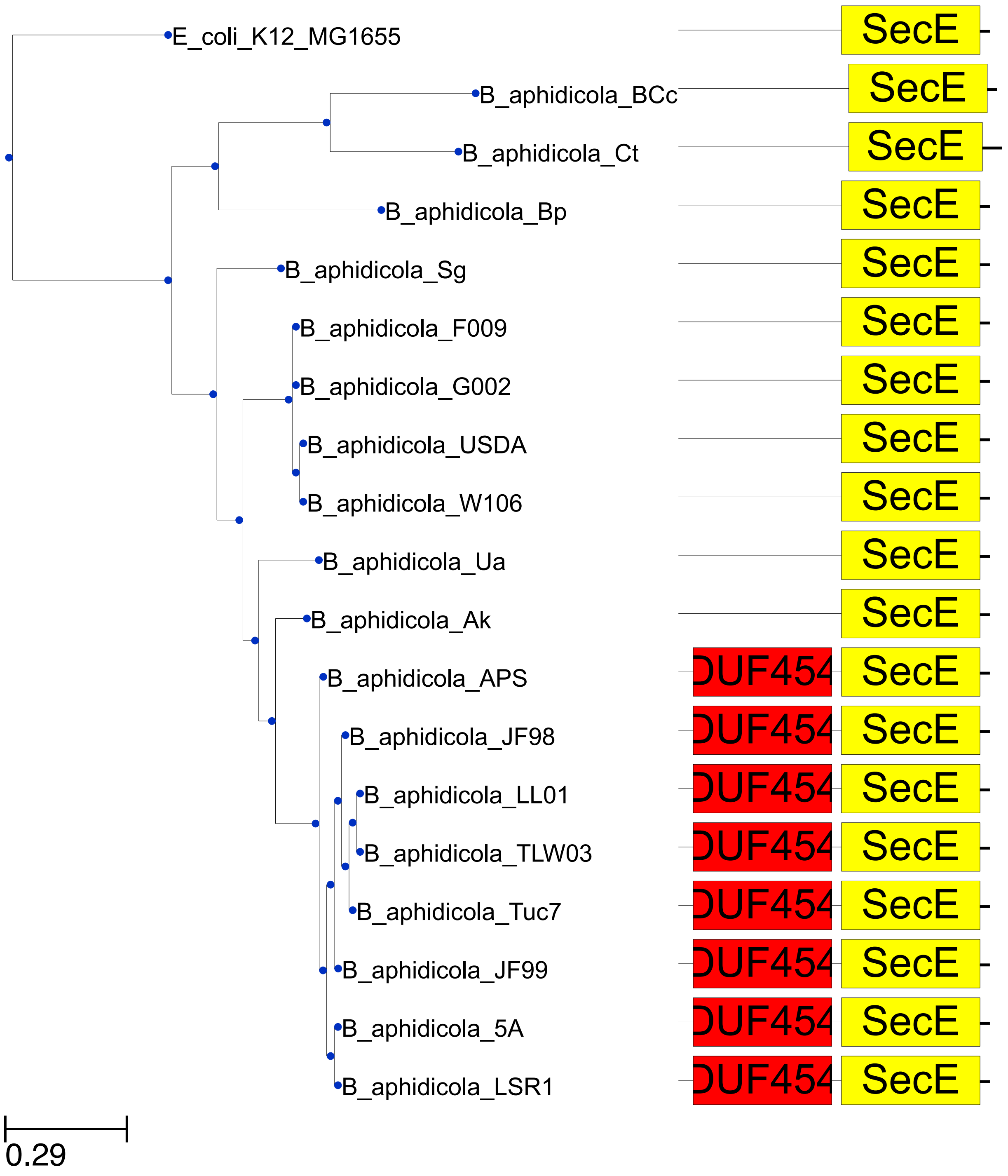

secE
NP_418408.1
preprotein translocase membrane subunit

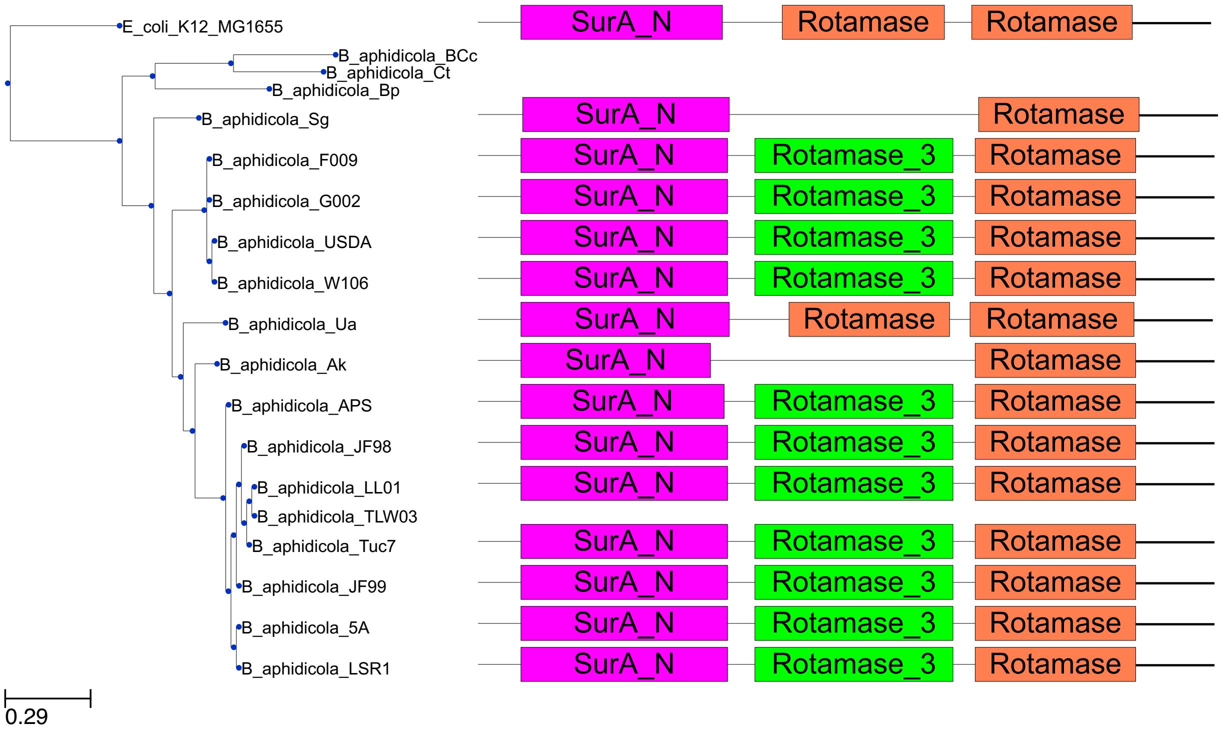

surA

NP_414595.1

peptidyl-prolyl cis-trans isomerase (PPIase)

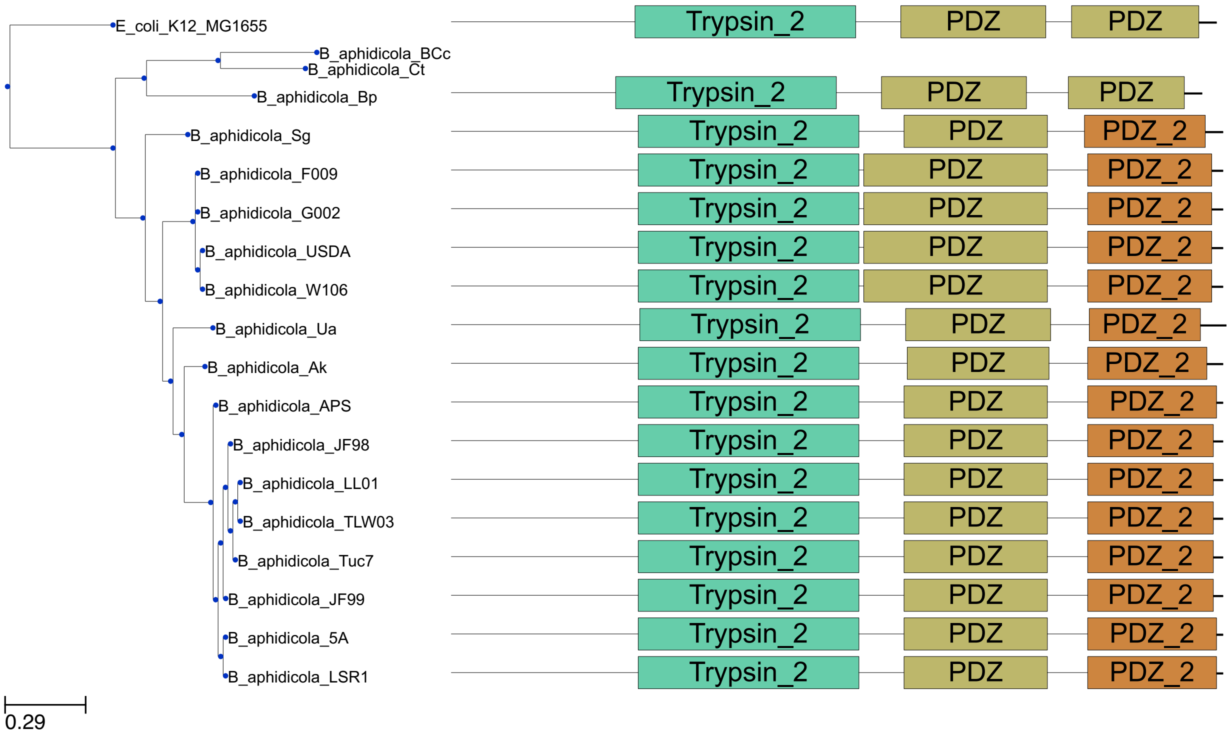

degP

NP_414703.1

"serine endoprotease (protease Do), membrane-associated"

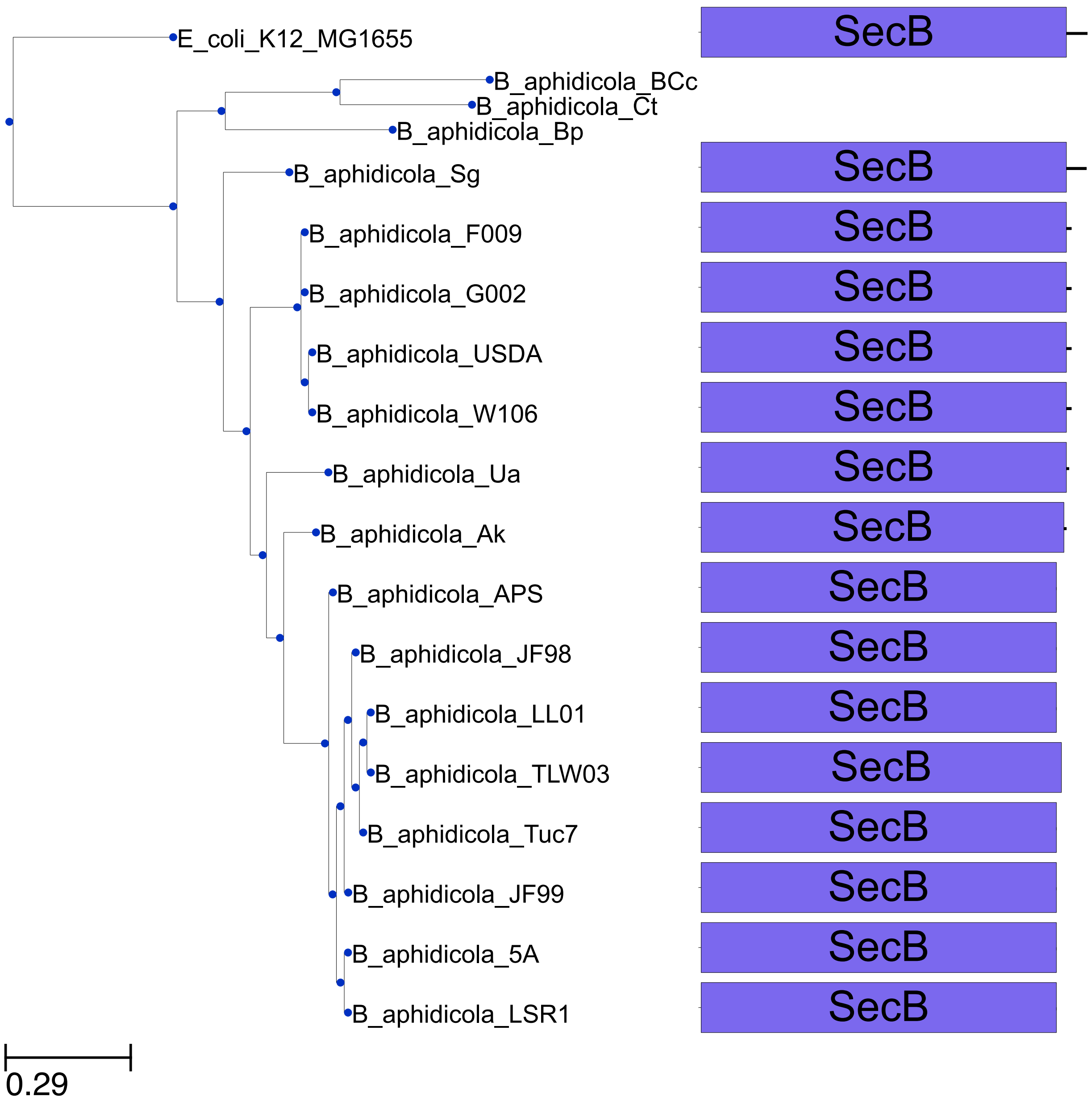

secB
NP_418066.1
protein export chaperone

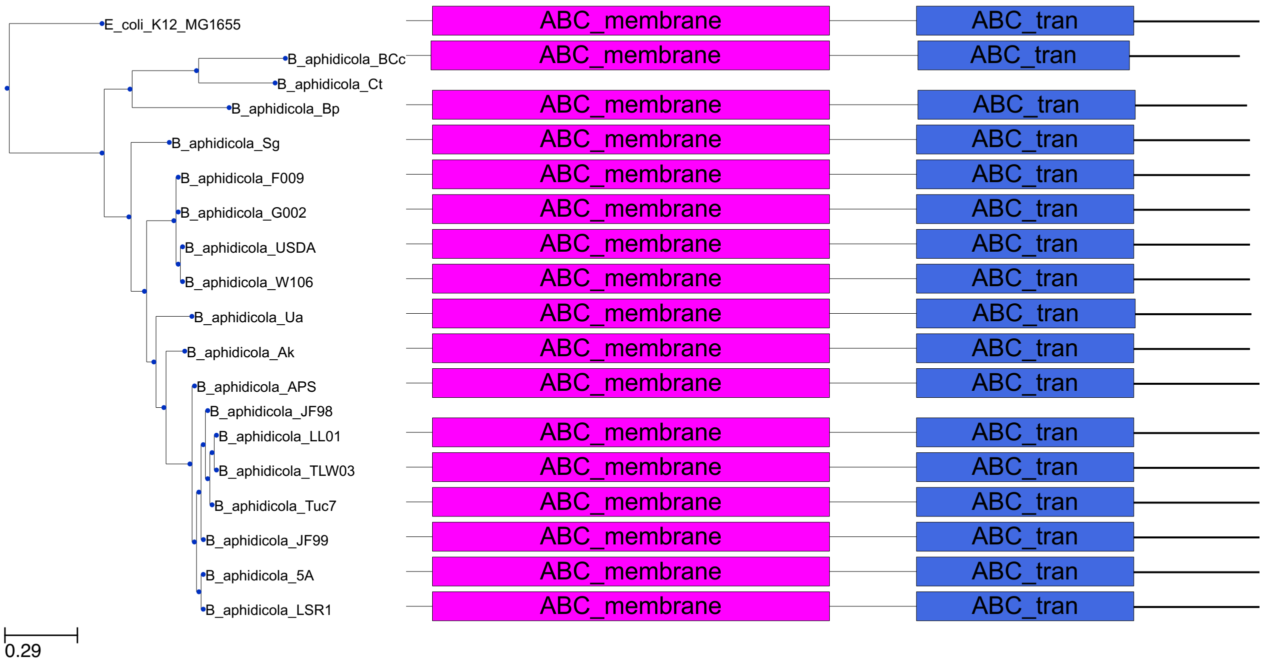

mdlA

NP_414982.1

putative multidrug ABC transporter ATPase

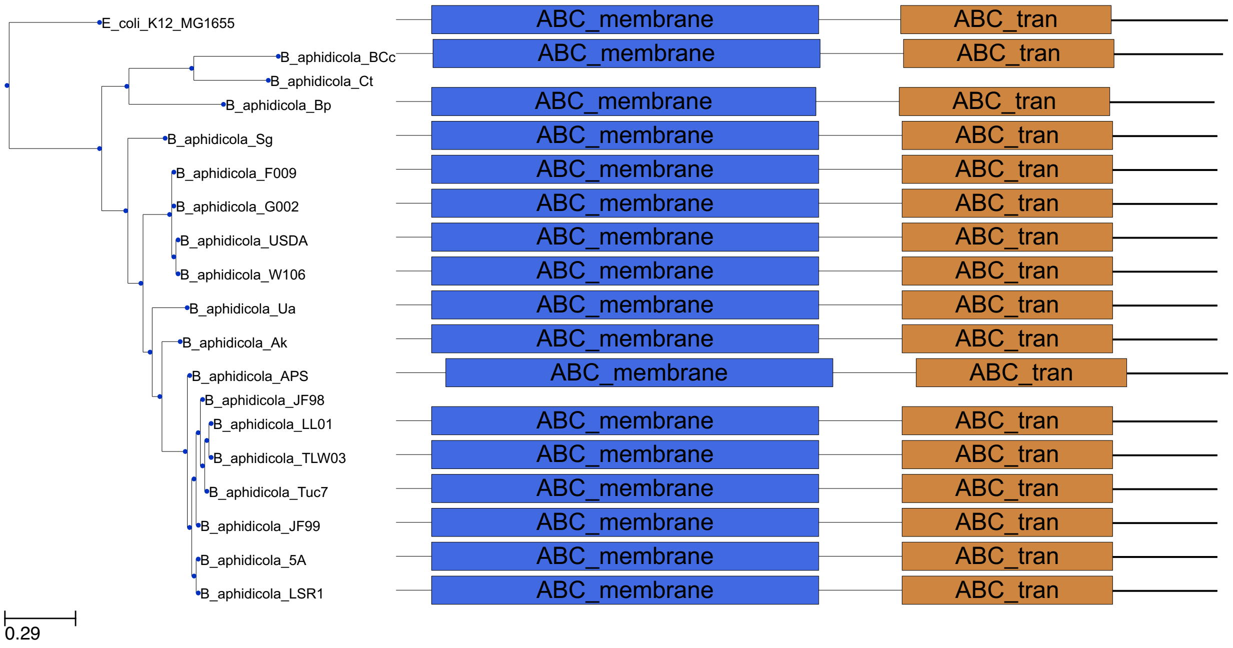

mdlB
NP_414983.1
putative multidrug ABC transporter ATPase

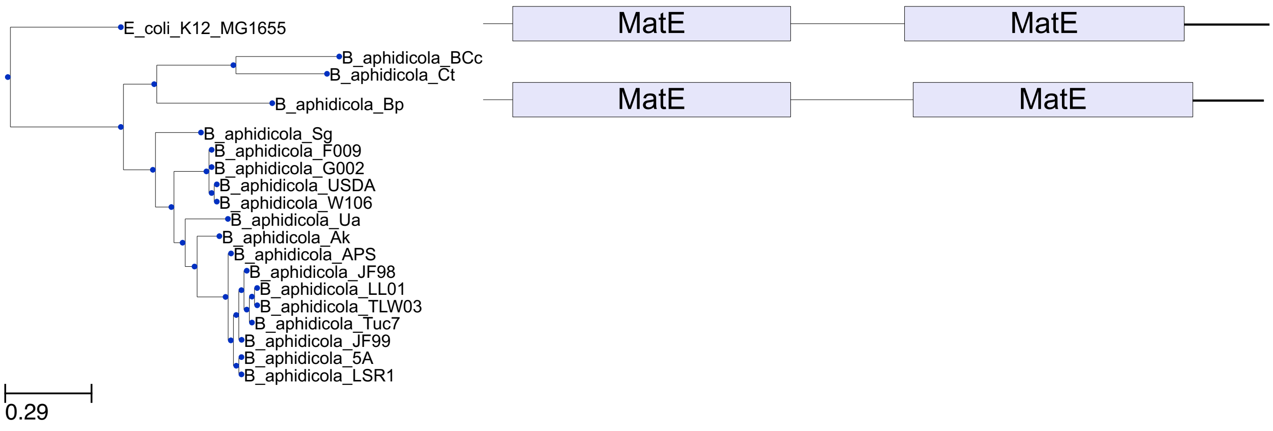

mdtK
YP_025307.1
multidrug efflux system transporter

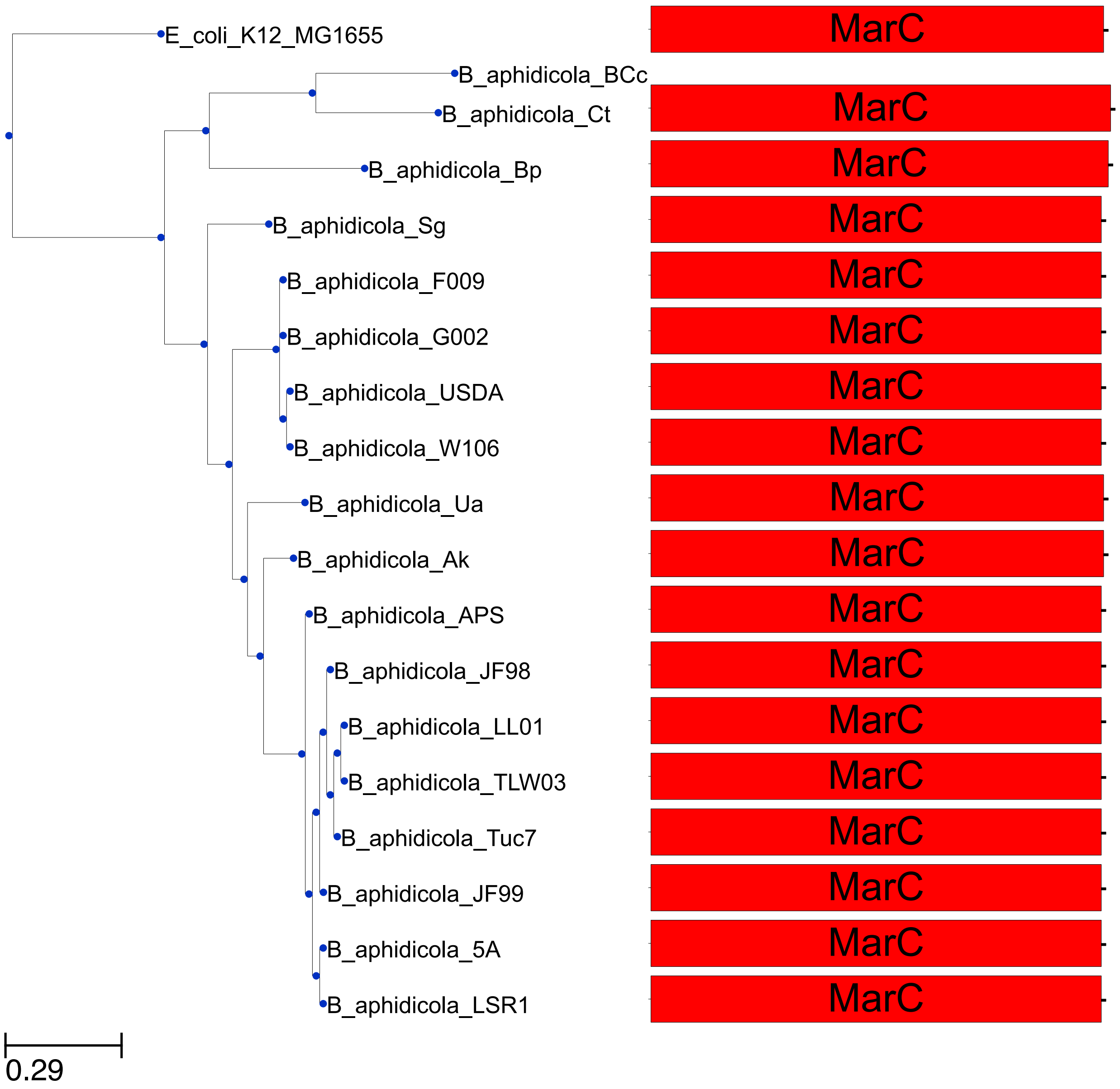

yhgN
NP_417892.1
UPF0056 family inner membrane protein
