# Supplementary material for: Testing the Domino Theory of Gene Loss in Buchnera aphidicola: The Relevance of Epistatic Interactions
Source: Life (Basel). 2018 May 29;8(2):17. doi: 10.3390/life8020017 (PMC6027505; doi:10.3390/life8020017)
Supplement: Supplementary file 1 [file life-08-00017-s001.zip › Martinez-Cano-etal-SuppFiles/Supplementary_material_2.pdf]

a)

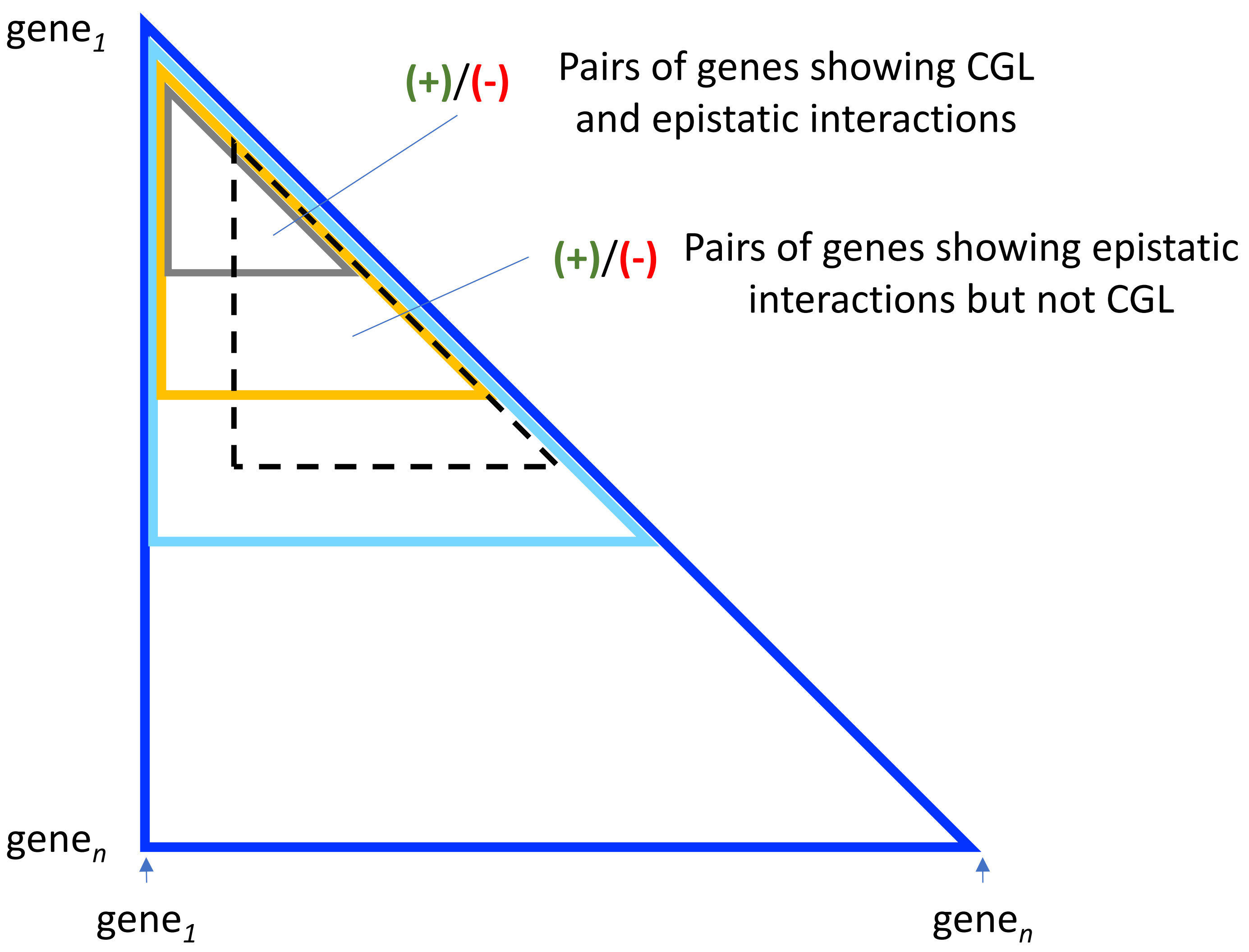


b)

| | FDR < 0.1 | | | | FDR < 0.45 | | | |
|---|---|---|---|---|---|---|---|---|
| | RM epistasis | | MM epistasis | | RM epistasis | | MM epistasis | |
| | (+) | (-) | (+) | (-) | (+) | (-) | (+) | (-) |
| CGL | 4 (1:1:2) | 0 (0:0:0) | 9 (0:5:4) | 4 (2:2:0) | 42 (12:21:9) | 13 (1:9:3) | 46 (5:23:18) | 48 (19:23:6) |
| No CGL | 403 (147:198:58) | 180 (34:94:52) | 288 (66:157:65) | 505 (194:253:58) | 365 (136:178:51) | 167 (33:85:49) | 251 (61:139:51) | 461 (177:232:52) |

(Essntial-Essential:Essential-Non-essential:Non-essential-Non-essential)

c)

| | E vs E pairs | Non-E vs Non-E pairs |
|---|---|---|
| Rich medium | (147+1)/34 = 4.4 | (58+2)/52 = 1.2 |
| Minimal medium | 66/(194+2) =0.3 | (65+4)/58 = 1.2 |

Essential genes showed more positive interactions in rich medium (148/34 ≈ 4.4) than non-essential genes (60/52 ≈ 1.2); and in minimal medium, essential genes showed more negative interactions (66/196 ≈ 0.3) than non-essential genes (69/58 ≈ 1.2).

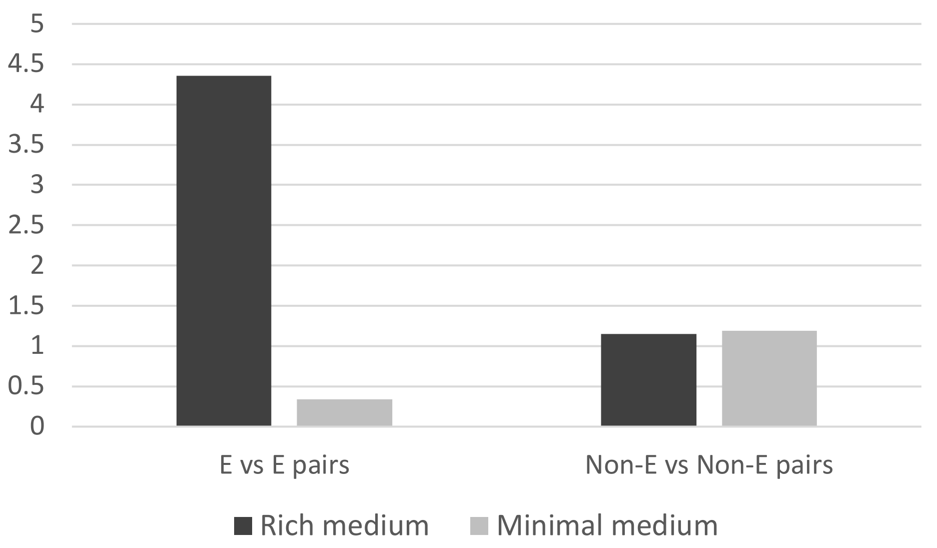


*E. coli*

Genes studied by Babu et al. (2011)

*E. coli* genes studied here with orthologs in *Buchnera*

Negative epistasis

Positive epistasis
